# Classification of Developmental Toxicants in a Human iPSC Transcriptomics-Based Test

**DOI:** 10.1021/acs.chemrestox.1c00392

**Published:** 2022-04-13

**Authors:** Anna Cherianidou, Florian Seidel, Franziska Kappenberg, Nadine Dreser, Jonathan Blum, Tanja Waldmann, Nils Blüthgen, Johannes Meisig, Katrin Madjar, Margit Henry, Tamara Rotshteyn, Rosemarie Marchan, Karolina Edlund, Marcel Leist, Jörg Rahnenführer, Agapios Sachinidis, Jan G. Hengstler

**Affiliations:** † Faculty of Medicine and University Hospital Cologne, Center for Physiology, Working Group Sachinidis, University of Cologne, Robert-Koch-Str. 39, 50931 Cologne, Germany; ‡ Leibniz Research Centre for Working Environment and Human Factors at the Technical University of Dortmund (IfADo), Ardeystrasse 67, 44139 Dortmund, Germany; § Department of Statistics, TU Dortmund University, Vogelpothsweg 87, 44227 Dortmund, Germany; ∥ In Vitro Toxicology and Biomedicine, Department of Biology, University of Konstanz, Universitätsstr. 10, P.O. Box M657, 78457 Konstanz, Germany; ⊥ Department of Advanced Cell Systems, trenzyme GmbH, Byk-Gulden-Str. 2, 78467 Konstanz, Germany; # Institute of Pathology, Charité-Universitätsmedizin Berlin, Chariteplatz 1, 10117 Berlin, Germany; ¶ IRI Life Sciences, Humboldt Universität zu Berlin, Philippstraße 13, Haus 18, 10115 Berlin, Germany; ∇ Center for Molecular Medicine Cologne (CMMC), University of Cologne, 50931 Cologne, Germany

## Abstract

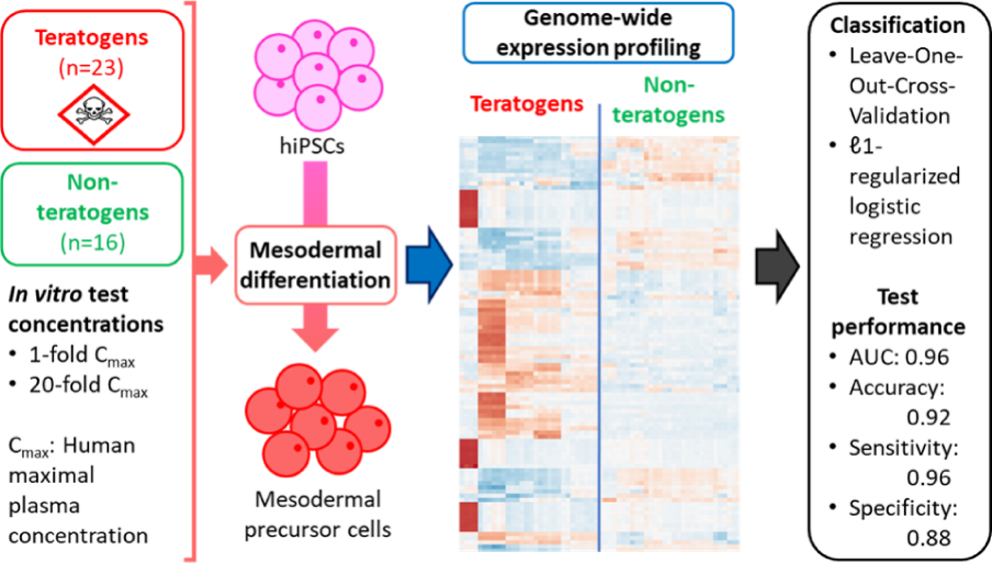

Despite the progress made in developmental toxicology, there is a great need for in vitro tests that identify developmental toxicants in relation to human oral doses and blood concentrations. In the present study, we established the hiPSC-based UKK2 in vitro test and analyzed genome-wide expression profiles of 23 known teratogens and 16 non-teratogens. Compounds were analyzed at the maximal plasma concentration (*C*_max_) and at 20-fold *C*_max_ for a 24 h incubation period in three independent experiments. Based on the 1000 probe sets with the highest variance and including information on cytotoxicity, penalized logistic regression with leave-one-out cross-validation was used to classify the compounds as test-positive or test-negative, reaching an area under the curve (AUC), accuracy, sensitivity, and specificity of 0.96, 0.92, 0.96, and 0.88, respectively. Omitting the cytotoxicity information reduced the test performance to an AUC of 0.94, an accuracy of 0.79, and a sensitivity of 0.74. A second method, which used the number of significantly deregulated probe sets to classify the compounds, resulted in a specificity of 1; however, the AUC (0.90), accuracy (0.90), and sensitivity (0.83) were inferior compared to those of the logistic regression-based procedure. Finally, no increased performance was achieved when the high test concentrations (20-fold *C*_max_) were used, in comparison to testing within the realistic clinical range (1-fold *C*_max_). In conclusion, although further optimization is required, for example, by including additional readouts and cell systems that model different developmental processes, the UKK2-test in its present form can support the early discovery-phase detection of human developmental toxicants.

## Introduction

Developmental toxicity testing aims to analyze disturbances during embryo-fetal development. Its importance became apparent after the thalidomide-induced disaster in the late 1950s, which could not be foreseen by the risk assessment strategies at that time.^1^ Modern guidelines for toxicity and developmental toxicity testing in regulatory risk assessment are complex, and while they do provide better prediction, they are associated with high costs and high numbers of animals for in vivo testing, especially in the context of the European Union regulation for the Registration, Evaluation, Authorisation and Restriction of Chemicals (REACH).^2^ In order to reduce the number of in vivo tests and animals used, several alternative in vitro technologies have been developed in the last few decades. For developmental toxicity testing in particular, pluripotent stem cells (PSCs),^3,4^ neural cells,^5,6^ and zebrafish^7^ have all been utilized as test organisms. However, none has yet been approved for regulatory risk assessment. A recent, novel approach using transcriptomics and human PSCs has demonstrated that compounds acting via a common mechanism, for example, histone deacetylase (HDAC) inhibitors or mercurials, can be differentiated via specific patterns of gene expression changes.^8−10^ A correlation was also recently observed between the expression of specific marker genes and disturbed neural rosette formation by PSC-derived neural progenitor cells.^11^ The transition from adaptive to cytotoxic responses was shown to be accompanied by changes in the expression of distinct groups of genes.^12^ Additionally, the identification of genomic biomarkers and early toxicity signatures was demonstrated using human-induced PSC (hiPSC)-derived cardiomyocytes for assessing the cardiotoxic potential.^13−15^

One of the major challenges of in vitro test development is to find a correlation between in vivo doses of test compounds that cause an increased risk of developmental toxicity and in vitro concentrations that lead to positive or negative test results. One strategy is to test concentrations in vitro that are related to the maximal blood concentration (*C*_max_) in vivo, which arises from a specific dose of interest, such as a therapeutic drug dose or from the uptake of an environmental compound. A further challenge is that in vitro testing of the *C*_max_ does not always optimally differentiate toxic from non-toxic compounds.^16^ For example, it has been reported that the best classification for hepato- and nephrotoxicities was obtained using in vitro concentrations that were higher than the *C*_max_.^17^

The goal of the present study was to analyze if transcriptomic analysis of hiPSCs that were differentiated according to an in vitro cardiomyogenic protocol^18,19^ by phasic activation of the Wnt-pathway and simultaneously exposed to test compounds will allow us to discriminate a set of teratogenic from non-teratogenic compounds; this procedure was further named UKK2. Based on genome-wide expression profiles, we asked if a penalized logistic regression-based classification method that used the 1000 probe sets with the highest variance is superior to a procedure that simply considers the number of significantly deregulated probe sets.

Moreover, we addressed whether teratogenic and non-teratogenic compounds can be better distinguished at the 1-fold *C*_max_ or 20-fold *C*_max_. Finally, we explored how to deal with cytotoxic test compound concentrations by examining if a compound could be classified as positive when the 1-fold or 20-fold *C*_max_ is cytotoxic. In the present study, we report that a penalized logistic regression-based method with leave-one-out cross-validation classified the analyzed set of known teratogenic and non-teratogenic compounds with an area-under-the-curve (AUC), accuracy, sensitivity, and specificity of 0.96, 0.92, 0.96, and 0.88, respectively, when gene expression and cytotoxicity were considered at 1-fold *C*_max_.

## Materials and Methods

### Test Compounds

The following test compounds were purchased from Sigma-Aldrich (St. Louis, Missouri, USA): 3,3′,5-triiodo-l-thyronine sodium salt (T6397), acitretin (PHR1523), ampicillin anhydrous (A9393), ascorbic acid (A0278), atorvastatin calcium (PHR1422), buspirone hydrochloride (B7148), carbamazepine (C4024), chlorpheniramine maleate salt (C3025), dextromethorphan HBr (PHR1018), doxorubicin hydrochloride (D2975000), doxylamine succinate (D3775), famotidine (F6889), folic acid (F7876), isotretinoin (PHR1188), leflunomide (PHR1378), levothyroxine (PHR1613), lithium chloride (L4408), magnesium chloride anhydrous (8147330500), methicillin sodium salt monohydrate (1410002), methotrexate (PHR1396), methylmercury(II)-chloride (33368), paroxetine hydrochloride (PHR1804), ranitidine hydrochloride (R101), retinol (17772), sucralose (PHR1342), thalidomide (T144), trichostatin A (T1952), and valproic acid (PHR1061). The following test chemicals were obtained from Biomol (Hamburg, Germany): actinomycin D (BVT-0089), entinostat/MS-275 (Cay13284), panobinostat (Cay13280), vinblastine sulfate salt (Cay11762), and vorinostat/SAHA (Cay10009929). Favipiravir (HY14768), teriflunomide/A-771726 (HY15405), and vismodegib (HY10440) were purchased from Hycultec (Beutelsbach, Germany). From Santa Cruz Biotechnology, Inc (Dallas, Texas, USA), 5,5-diphenylhydantoin sodium salt (sc-214337) and diphenhydramine hydrochloride (sc-204729) were obtained. The compounds were dissolved and stored at concentrations that were 20,000-fold *C*_max_ in 100% DMSO or alternatively in distilled water, if soluble.

### Human-Induced Pluripotent Stem Cells

The hiPSC line SBAD2 was obtained from Prof. Marcel Leist (University of Konstanz), which was originally procured for the StemBANCC project (http://stembancc.org).^20^ The identity of the obtained SBAD2 hiPSCs was confirmed by short tandem repeat profiling performed at the Leibniz-Institute DSMZ (German Collection of Microorganisms and Cell Cultures). For the UKK2 test system, the cells were cultured and maintained in StemMACS iPS-Brew XF medium (Miltenyi Biotec, Germany) on plates coated with Matrigel (Corning GmbH, Kaiserlautern, Germany), as previously described.^21^

### Differentiation of hiPSCs to Germ Layer Cell Types (UKK2 Test System)

Undifferentiated hiPSC cells were dissociated with CTS TrypLE Select Enzyme (Thermo Fisher Scientific, Germany), seeded at a density of 600,000 cells per well on Matrigel-coated 6-well plates in StemMACS iPS-Brew XF medium, and supplemented with 10 μM ROCK inhibitor Y-27632 (Calbiochem, Merck KGaA, Darmstadt, Germany). On the following day, the medium was changed to StemMACS iPS-Brew XF medium without the ROCK inhibitor. On day 0, differentiation was induced by adding 10 μM of the Wnt activator, CHIR99021 (R&D Systems, Minneapolis, USA) in RPMI 1640 GlutaMAX medium (Thermo Fisher Scientific, Germany) with the B-27 Supplement, and without insulin (Thermo Fisher Scientific, Germany). At the same time, the cells were incubated (5% CO_2_, 37 °C) with the test compounds at 1-fold *C*_max_ and 1.67-, 10-, or 20-fold *C*_max_ concentration, as well as the vehicle alone (0.1% DMSO). The compounds leflunomide and teriflunomide were tested at a DMSO concentration of 0.5% and compared to a 0.5% DMSO vehicle control. After 24 h, the cells were collected for RNA extraction. A test compound concentration was considered as cytotoxic if upon microscopic inspection no adherent cells were visible or if the harvested amount of RNA was below 2 μg per well of the 6-well plate. For each tested condition, three biological replicates were generated, except for isotretinoin at 1-fold *C*_max_ and thalidomide at 1-fold- and 20-fold *C*_max_, where six biological replicates were generated.

### RNA Isolation

The cells were homogenized with the TRIzol lysis reagent (Thermo Fisher Scientific, Germany), and total RNA was extracted and purified using the RNeasy Mini Kit (Qiagen, Germany), according to the manufacturer’s instructions. Usual amounts of harvested RNA per well of the 6-well plate under control conditions were 33 μg. Concentration and purity of the isolated RNA were evaluated using a Nanodrop ND-1000 spectrophotometer (Thermo Fisher Scientific, Germany). The extracted RNA was then further processed for microarray gene expression studies using reagents and instruments from Affymetrix.

### Affymetrix Microarray Studies

For microarray gene expression studies, 100 ng of total RNA was used. The samples were amplified and labeled with biotin using GeneChip 3′ IVT Express Kit per the manufacturer’s instructions (Affymetrix, High Wycombe, UK). Then, samples were purified using magnetic beads and fragmented. 12.5 μg of fragmented RNA samples were hybridized onto Affymetrix Human Genome U133 Plus 2.0 arrays (Affymetrix, Santa Clara, CA, USA). The microarray hybridization step was performed in an Affymetrix GeneChip Hybridization Oven-645 for 16 h at 45 °C and 60 rpm. Washing and staining of the hybridized arrays were completed using the GeneChip HWS Kit (Affymetrix, High Wycombe, United Kingdom) and Affymetrix GeneChip Fluidics Station-450. Finally, the stained arrays were scanned with the Affymetrix Gene-Chip Scanner-3000-7G, and quality control was performed with Affymetrix GCOS software. The generated data files were used for further statistical analysis.

### Statistical Methods

All analyses were conducted using the statistical software R, version 4.0.5,^22^ with additional R-packages as indicated in the following sections. For each non-cytotoxic compound and concentration, three independent biological replicates were considered, except for 9-cis-retinoic acid at 20-fold *C*_max_, where one replicate was excluded from further analysis after preprocessing. Further exceptions were isotretinoin at 1-fold *C*_max_ and thalidomide at 1-fold and 20-fold *C*_max_, where six replicates were available.

### Preprocessing

Affymetrix microarray analysis was performed using HG-U 133 Plus 2.0 arrays, yielding CEL-files. Preprocessing of the data consisted of the three steps: background correction, normalization, and summarization using the frozen robust multiarray average (fRMA) algorithm, which yielded expression values for 54,675 probe sets (PS). The R-packages affy,^23^ frma,^24^ and hgu133plus2frmavecs^25^ were used.

Batch effects were avoided by normalizing with respect to the batch-wise control as follows: batch-wise mean values of the control samples were calculated for each gene, and these mean values were subtracted from the individual expression values of the non-control samples.

### Principal Component Analysis Plots

A principal component analysis (PCA) was carried out based on the normalized expression values, where the mean value of the corresponding control samples was subtracted. For each condition, that is, for each combination of compound and concentration separately, the PS-wise mean value across the samples was calculated.

### LIMMA Analysis

Differential expression was calculated using the R-package LIMMA.^26^ The complete set of PS was considered for an empirical Bayes adjustment of the variance estimates of single PS. This is a form of a moderated *t*-test, abbreviated here as “LIMMA *t*-test”. The resulting *p*-values were multiplicity-adjusted to control the false discovery rate (FDR) using the Benjamini–Hochberg procedure.^27^ As a result, for each compound, a gene list was obtained with the corresponding estimates for fold change (FC), log2 fold change, and *p*-values of the LIMMA *t*-test (unadjusted and FDR-adjusted). For isotretinoin at 1-fold *C*_max_ and thalidomide at 1-fold and 20-fold *C*_max_, two such lists were obtained, each based on three replicates.

### Classification Using the Number of Significant Probe Sets (SPS-Procedure)

An initial classification of the compounds was obtained by only considering the number of significant probe sets (SPS). A probe set was considered to be significant if both the FDR-adjusted *p*-value from the LIMMA *t*-test was smaller than 0.05 and the absolute value of the FC was larger than 2. For each compound at a specific concentration (further named “condition”), the number of SPS was determined and used for the classification procedure. Next, the number of SPS (further named “threshold”) was analyzed with respect to accuracy. For this purpose, all conditions with the number of SPS higher than the threshold were considered as test-positive; whereas the conditions with a lower number of SPS than the threshold were considered to be test-negative. Finally, the threshold with the highest accuracy was identified.

To assess the quality of the classification procedures, the following measures were calculated: sensitivity (true positive rate) is the number of true positives divided by the sum of true positives and false negatives. Specificity (true negative rate) is the number of true negatives divided by the sum of true negatives and false positives. Accuracy is the proportion of correctly classified conditions, that is, the sum of true positives and true negatives divided by the number of all conditions. The receiver operator characteristic (ROC)-based AUC was calculated as follows: for each possible cutoff used as a threshold, predictions were made for each of the conditions based on which sensitivity and specificity were calculated. The ROC-curve was obtained by plotting all pairs of (1-specificity) and sensitivity against each other. The AUC was determined as the area under this ROC-curve.

### Penalized Logistic Regression with Leave-One-Out Cross Validation (Top-1000-Procedure)

The second classification procedure used penalized logistic regression and was constructed based on the normalized gene expression values. A leave-one-out cross-validation approach was used, which was iterated over the 34 non-cytotoxic compounds, where in each iteration, all samples corresponding to one compound were left out of the dataset. For the remaining 33 compounds, the difference between test compound-exposed samples and corresponding controls was calculated and the empirical variance of the difference was determined for each PS. An -regularized logistic regression-based classifier was trained on the 1000 PS with the highest variance and evaluated on the compound that was left out, yielding a probability for each sample of the left-out compound. Probabilities corresponding to samples of the same concentration value were summarized by the mean value. The penalty parameter “lambda” in the -regularized logistic regression was optimized via 10-fold cross-validation to minimize the mean cross-validated error.

A threshold was chosen for the predicted probabilities, where all conditions with a probability higher than this threshold were considered as test-positive and all conditions with a probability lower than this threshold were considered as test-negative. The threshold was set to a predicted probability, where the accuracy was maximal. The measures sensitivity, specificity, accuracy, and AUC were calculated as explained above.

The R-package mlr^28^ was used as a framework for the classification tasks, together with the package glmnet^29^ for the calculation of the specific classifier.

### Venn Diagrams, Top Genes, GO Group Over-representation, and KEGG Pathway Enrichment Analyses

Venn diagrams were created to compare sets of SPS for non-teratogenic and teratogenic compounds, once based on all sets of SPS, once for SPS that were upregulated, and once for SPS that were downregulated.

For each element of the Venn diagrams, top lists of the corresponding probe sets and genes were determined. For each PS, the number of compounds that led to differential expression was determined. This was used as the first level for the ranking. The arithmetic mean of the log2 fold change (or the arithmetic mean of the absolute values of the log2 fold change, in case where all SPS were considered) of each SPS across all compounds, where it was differentially expressed, was calculated. This value was used as the second level for the ranking. For the translation of the top probe sets to the top genes, only the highest ranked probe set for each gene was considered and all lower ranked probe sets which represented the same gene were removed. Additionally, for the displayed top10-lists in Figures 5B and S2–S6B, only probe sets with the suffixes _at, _a_at, and _s_at were considered due to their high specificity.

For each element of a Venn diagram (i.e., the set of SPS that were significant for non-teratogens only, significant for teratogens only, and the overlap, i.e., significant for both non-teratogens and teratogens), over-representation analyses were conducted as follows: SPS were assigned to gene ontology (GO) groups according to their biological processes. Using Fisher’s exact test, it was statistically tested whether more PS in the respective groups were differentially expressed than expected at random. In the “elim” approach, this procedure was conducted bottom-up with respect to the GO group hierarchy, and PS that were already contained in a more specific GO group were not further considered in more general groups.^30^

The list of significant GO groups, where a group was called significant if the FDR-adjusted p-value of the “elim” method was smaller than 0.05, was analyzed with respect to their overlap using Venn diagrams.

Additionally, SPS were assigned to their respective Kyoto encyclopedia of genes and genomes (KEGG) pathway, and Fisher’s exact test was used to statistically examine whether more PS assigned to a specific pathway were differentially expressed than expected at random.

The GO analysis of the overall 1160 PS that were included in any of the 34 individual compound-specific top-1000-classifiers was performed as described before. Briefly, PS were assigned to GO groups according to their biological process. Using Fisher’s exact test, it was statistically tested whether more PS in the respective group were a part of the 1160 PS than expected at random. In the “elim” approach, this procedure was conducted bottom-up with respect to the GO group hierarchy, and PS that were already contained in a more specific GO group were not further considered in more general groups.

GO group analyses were conducted using the R package topGO,^31^ and KEGG pathway analyses were conducted using the R package clusterProfiler.^32^

## Results

### Selection of Test Compounds and Concentrations

We established the UKK2 test based on a published cardiomyocyte differentiation protocol^18,19^ using an exposure period with test compounds of 24 h. To study if transcriptomics distinguish between teratogens and non-teratogens, a set of test compounds was selected (Table 1). A first inclusion criterion was the availability of published information on whether the selected compound was teratogenic or non-teratogenic in humans and/or animals (Table S1). Information from the www.drugs.com database was also used, including the narrative sections, as well as the pregnancy risk categories A and B for non-teratogenic, and D and X for teratogenic compounds as defined by the U.S. Food and Drug Administration (FDA) and the Australian Therapeutic Goods Administration (TGA) (Table 1). The second inclusion criterion was the availability of pharmacokinetic information from clinical studies and other resources (Tables S2 and S3) in order to calculate therapeutic compound concentrations (*C*_max_) and 20-fold *C*_max_ for use in the in vitro testing (Table 1). Information on the ability of non-teratogenic compounds to cross the human placenta was also collected (Table S4). A third inclusion criterion was that the compound was sufficiently soluble so as not to exceed 0.5% DMSO in the culture medium for the *C*_max_. Based on these three criteria, 16 non-teratogens and 23 teratogens were selected (Table 1). Solubility was sufficient to test all test compounds at 1- and 20-fold *C*_max_ with the exception of leflunomide, phenytoin, teriflunomide, and vismodegib that were tested at only 1-fold *C*_max_, as well as carbamazepine that was tested at 1- and 10-fold *C*_max_ due to solubility limitations. Valproic acid was tested at 1- and 1.67-fold *C*_max_ due to known cytotoxic effects at higher concentrations.

**Table 1:** Substances and Applied Concentrations in the UKK2 Test System

				tested concentration [μM]	
compound	abbreviation	pregnancy category^a^	drug class	1-fold*C*_max_^b^	20-fold *C*_max_^b^
Non-teratogens					
ampicillin	AMP	A, B	antibiotic	107	2140
ascorbic acid	ASC	A	vitamin	200	4000
buspirone	BSP	B	anxiolytic, serotonin 5-HT1A receptor agonist	0.0244	0.488
chlorpheniramine	CPA	B	antihistamine, histamine H1 receptor antagonist	0.0304	0.608
dextromethorphan	DEX	A	antitussive and psychoactive agent	0.15	3
diphenhydramine	DPH	A, B	antihistamine, histamine H1 receptor antagonist	0.3	6
doxylamine	DOA	A	antihistamine, histamine H1 receptor antagonist	0.38	7.6
famotidine	FAM	B	antihistamine, histamine H2 receptor antagonist	1.06	21.2
folic acid	FOA	A	vitamin	0.38	7.6
levothyroxine	LEV	A	synthetic thyroid hormone	0.077	1.54
liothyronine	LIO	A	synthetic thyroid hormone	0.00307	0.06145
magnesium (chloride)	MAG	n/a	dietary supplement	1200	24000
methicillin	MET	B	antibiotic	140	2800
ranitidine	RAN	B	antihistamine, histamine H2 receptor antagonist	0.8	16
retinol	RET	n/a	vitamin and retinoid	1	20
sucralose	SUC	n/a	artificial sweetener	2.5	50
Teratogens					
9-*cis*-retinoic acid	9RA	D	retinoid, RAR and RXR ligand	1	20
acitretin	ACI	X	retinoid, RAR activator	1.2	24
actinomycin D	ACD	D	antineoplastic agent, RNA synthesis inhibitor	0.1	2
atorvastatin	ATO	X^56,57^	antilipemic agent, HMG-CoA reductase inhibitor	0.54	10.8
carbamazepine	CMZ	D	anticonvulsant, voltage-gated sodium channel blocker	19	10-fold *C*_max_: 190^d^
doxorubicin	DXR	D	antineoplastic agent, affects DNA and related proteins; produces ROS	1.84	36.8
entinostat	ENT	n/a	potential antineoplastic agent, HDAC inhibitor	0.2	4
favipiravir	FPV	n/a	antiviral drug, selective inhibitor of RNA polymerase of influenza virus	382	7600
isotretinoin	ISO	X	retinoid, RAR ligand	1.7	34
leflunomide	LFL	X	anti-inflammatory agent, DHODH inhibitor	370	d
lithium (chloride)	LTH	D	mood stabilizer	1000	20000
methotrexate	MTX	D/X	antineoplastic, dihydrofolate reductase inhibitor	1	20
methylmercury	MEM	n/a	bioaccumulative environmental toxicant, hypothesized ROS production	0.020	0.4
panobinostat	PAN	n/a, (D)^c^	antineoplastic agent, HDAC inhibitor	0.06	1.2
paroxetine	PAX	D	antidepressant, SSR inhibitor	1.2	24
phenytoin	PHE	D	anticonvulsant, voltage-gated sodium channel blocker	20	d
teriflunomide	TER	X	anti-inflammatory agent, DHODH inhibitor	370	d
thalidomide	THD	X	antiangiogenic	3.9	78
trichostatin A	TSA	n/a	antifungal antibiotic, HDAC inhibitor	0.01	0.2
valproic acid	VPA	D, X^58^	anticonvulsant, voltage-gated sodium channel blocker, antifolate agent, HDAC inhibitor	600	1.67-fold *C*_max_: 1000^d^
vinblastine	VIN	D	antimitotic agent, affects microtubule dynamics	0.0247	0.494
vismodegib	VIS	X	antineoplastic agent, hedgehog pathway inhibitor	20	d
vorinostat	VST	D	antineoplastic agent, HDAC inhibitor	3	60

a U.S. Food and Drug Administration (FDA) and Australian Therapeutic Goods Administration (TGA) pregnancy categories: A: Compounds are safe to use during pregnancy, proven by well-controlled studies in humans or large quantity of data from pregnant women; B: Compounds are considered to be safe but lack sufficient human data; C and D: Compounds showed little or some evidence of teratogenicity in humans or animals; X: Compounds with known teratogenic activity in humans or with a suspected high teratogenic potential based on animal experiments; n/a = not available; Information was obtained from www.drugs.com (accessed on November 2020) if not stated otherwise.

b Maximal plasma or blood concentrations which were usually observed in humans after the administration of therapeutic compound doses (Tables S2 and S3). Fetal enrichment was considered if relevant (Table S4).

c Approved but not assigned (Recommendation: D).

d Carbamazepine and valproic acid were tested at 10-fold and 1.67-fold *C*_max_, respectively, instead of 20-fold *C*_max_; leflunomide, phenytoin, teriflunomide, and vismodegib were tested at 1-fold *C*_max_ due to limited solubility.

### Gene Expression Profiling

**Figure 1:**
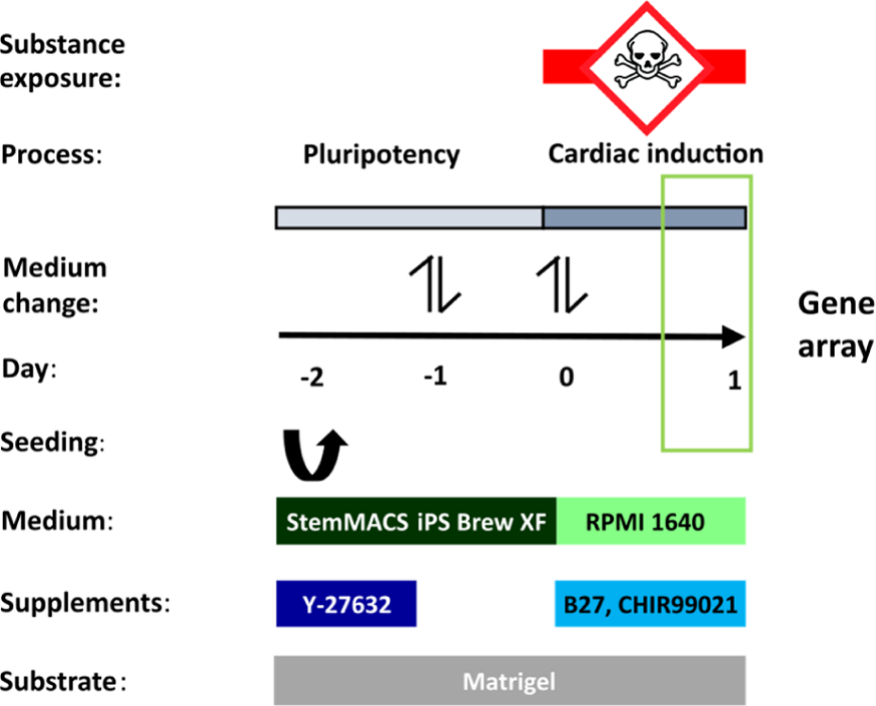
UKK2-test. Overview of the experimental design depicts the protocol from day −2 to day 1. In the pluripotency phase, the applied medium StemMACS iPS Brew XF maintained the hiPSCs’ pluripotent state. The supplement Y-27632 given on the day of seeding (day −2) supported the survival of hiPSCs, which were seeded as single cells on Matrigel. From day 0 to 1, the change to RPMI 1640 media spiked with B27 and CHIR99021 initiated cardiac differentiation of the cells. Simultaneously, cells were exposed to potential (non-)developmental toxic substances for a total of 24 h. On day 1, cells were harvested for gene array analysis. Medium changes were done as indicated on day −1 and day 0.

**Figure 2:**
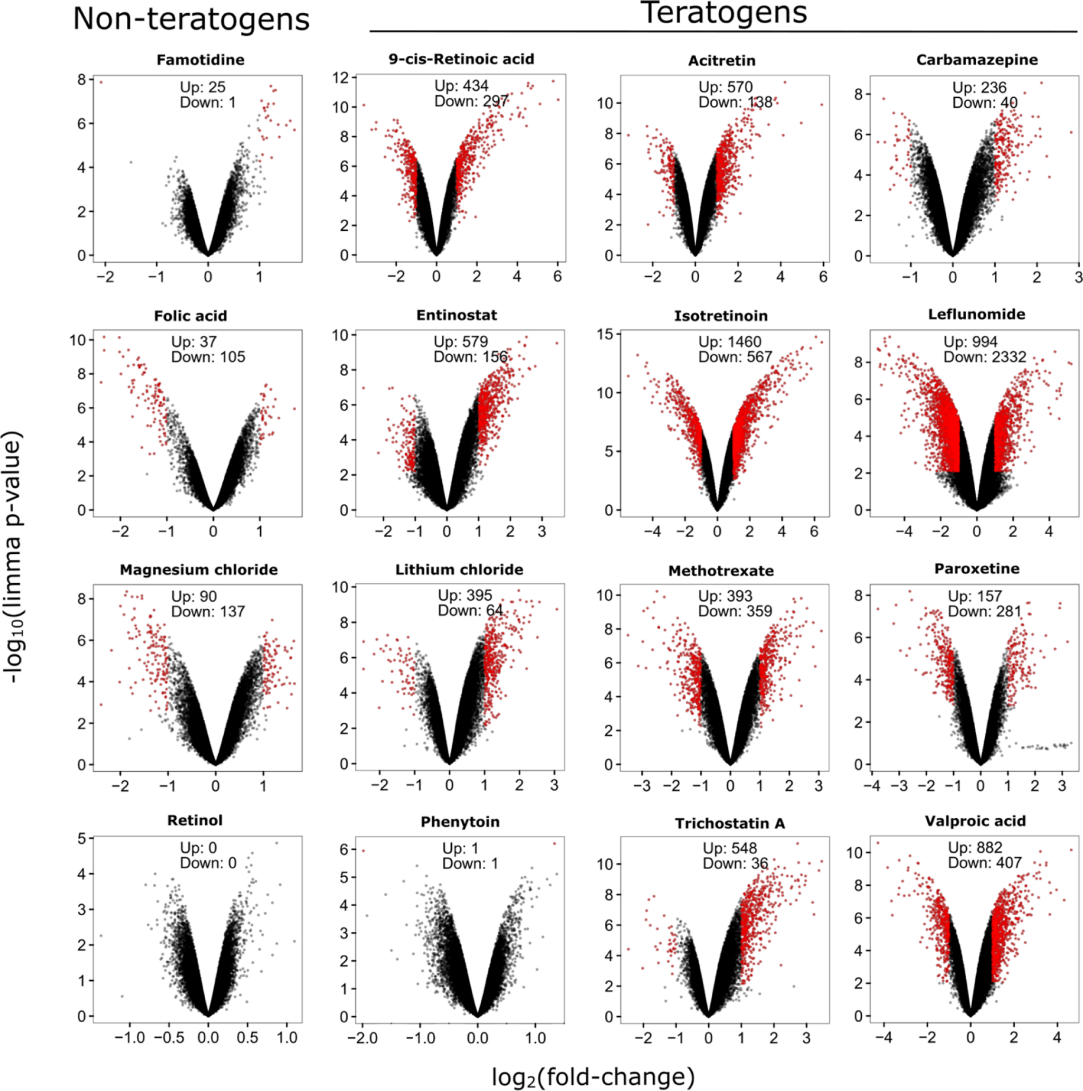
Volcano plots of deregulated probe sets of selected test compounds. Volcano plots show genome-wide expression changes in substance-exposed SBAD2 cells for a representative subset of known teratogens and non-teratogens at therapeutic 1-fold *C*_max_ concentrations. Each dot represents one out of 54,675 probe sets from the Affymetrix gene chips. The fold-change of the differentially expressed probe sets in substance-exposed cells is given on the logarithmic *x*-axis and the corresponding p-values of the LIMMA analyses are given on the negative, logarithmic *y*-axis. Red dots represent probe sets with a statistically significant, FDR-adjusted *p*-value < 0.05 and an absolute fold-change > 2. The numbers of up- and downregulated red-dot-probe sets are indicated.

All test compounds were analyzed in three independent experiments with microarrays after 24 h incubation periods using the protocol summarized in Figure 1. The genome-wide gene expression changes are given in volcano plots, which illustrate a representative selection of non-teratogenic and teratogenic compounds at *C*_max_ (Figure 2) (the plots for all compounds and concentrations can be found in the Supporting Information). To identify meaningful changes, only probe sets that were at least 2-fold deregulated and significantly altered (FDR-adjusted *p*-value < 0.05) were considered. The findings suggest that fewer genes were significantly deregulated for the non-teratogens than the teratogens; however, an all-or-nothing situation was not observed because some non-teratogenic compounds, for example, folic acid and magnesium chloride, also induced significant expression changes. An overview of the number of up- and downregulated probe sets at 1- and 20-fold *C*_max_ and the cytotoxicity status is given in Table 2; all raw data are available in the Gene Expression Omnibus database, accessible under GSE187001.

**Table 2:** Cytotoxicity and Number of Significantly Deregulated Probe Sets in Compound-Exposed Cells

			number of up-/downregulated probe sets^c^			
	cytotoxicity^b^		1-fold *C*_max_^a^		20-fold *C*_max_^a^	
compounds	1-fold *C*_max_^a^	20-fold *C*_max_^a^	up	down	up	down
Non-teratogens						
ampicillin	no	no	52	84	14	14
ascorbic acid	no	no	47	58	270	126
buspirone	no	no	39	5	45	6
chlorpheniramine	no	no	44	5	35	6
dextromethorphan	no	no	26	93	15	17
diphenhydramine	no	no	0	0	3	33
doxylamine	no	no	63	12	60	82
famotidine	no	no	25	1	21	2
folic acid	no	no	37	105	24	107
levothyroxine	no	no	77	131	9	4
liothyronine	no	no	103	74	26	10
magnesium chloride	no	no	90	137	461	333
methicillin	no	no	26	24	45	13
ranitidine	no	no	104	12	102	11
retinol	no	no	0	0	29	4
sucralose	no	no	153	38	136	30
Teratogens						
9-*cis*-retinoic acid	no	no	434	297	459	209
acitretin	no	no	570	138	437	221
actinomycin D	yes	yes	toxic	toxic	toxic	toxic
atorvastatin	no	no	123	5	235	129
carbamazepine	no	no^d^	236	40	551^d^	431^d^
doxorubicin	yes	yes	toxic	toxic	toxic	toxic
entinostat	no	no	579	156	2916	1336
favipiravir	no	no	150	11	686	405
isotretinoin	no	no	1135	580	1154	536
leflunomide	no	d	994	2332	d	d
lithium chloride	no	no	395	64	1176	389
methotrexate	no	no	393	359	359	471
methylmercury	no	no	328	49	108	16
panobinostat	yes	yes	toxic	toxic	toxic	toxic
paroxetine	no	no	157	281	147	473
phenytoin	no	d	1	1	d	d
teriflunomide	no	d	881	620	d	d
thalidomide	no	no	304	238	694	314
trichostatin A	no	yes	548	36	toxic	toxic
valproic acid	no	no^d^	882	407	1827^d^	731^d^
vinblastine	yes	yes	toxic	toxic	toxic	toxic
vismodegib	no	d	14	18	d	d
vorinostat	yes	yes	toxic	toxic	toxic	toxic

a Maximal plasma or blood concentrations which were usually observed in humans after the administration of therapeutic compound doses (Tables S2 and S3). Fetal enrichment was considered if relevant (Table S4).

b Yes, if the compound was highly cytotoxic; No, if the compound showed no cytotoxicity.

c Gene array probe sets that were deregulated with an FDR-adjusted *p*-value < 0.05 and an absolute fold-change > 2 compared to untreated control cells.

d Carbamazepine and valproic acid were tested at 10-fold and 1.67-fold *C*_max_, respectively, instead of 20-fold *C*_max_; leflunomide, phenytoin, teriflunomide, and vismodegib were tested at 1-fold *C*_max_ due to limited solubility.

**Figure 3:**
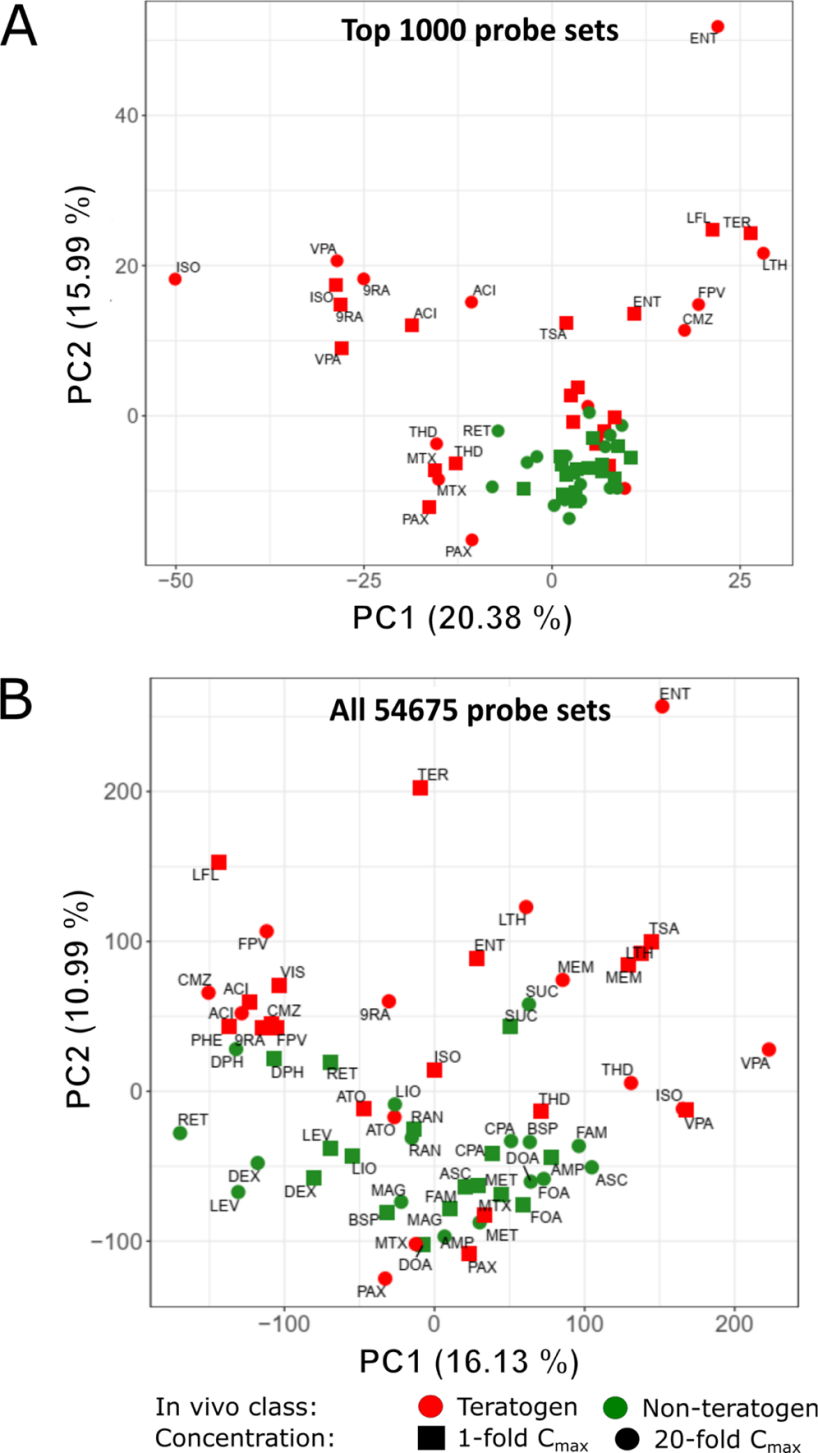
PCA of the teratogenic and non-teratogenic compounds. Two PCA plots are presented for (A) 1000 probe sets with the highest variance across the mean of the condition-wise samples and (B) all 54,675 probe sets. Green and red tags represent in vivo non-teratogens and teratogens, respectively. 1-fold *C*_max_ and 20-fold *C*_max_ concentrations are indicated by squares and circles, respectively. The distribution of the data points on the *x*-axis is given by the PC 1 and on the *y*-axis by PC2. The percentages in parentheses denote the proportion of explained variance for the respective PC. Compound abbreviations are explained in Table 1.

PCA showed that the non-teratogenic substances clustered closely together, partly intermixed with a subset of teratogenic substances, while the teratogenic substances were widely spread along both PC1 and PC2 (Figure 3). The PCA was performed based on the 1000 probe sets with the highest variance (Figure 3A) and on all 54,675 analyzed probe sets (Figure 3B). Using the top-1000 probe sets, the non-teratogenic compounds clustered more closely together compared to the analysis with all probe sets, suggesting that the 1000 probe sets with highest variance may offer an option for classifier construction.

### Differentiation of Teratogenic and Non-teratogenic Compounds Based on Gene Expression and Cytotoxicity

**Figure 4:**
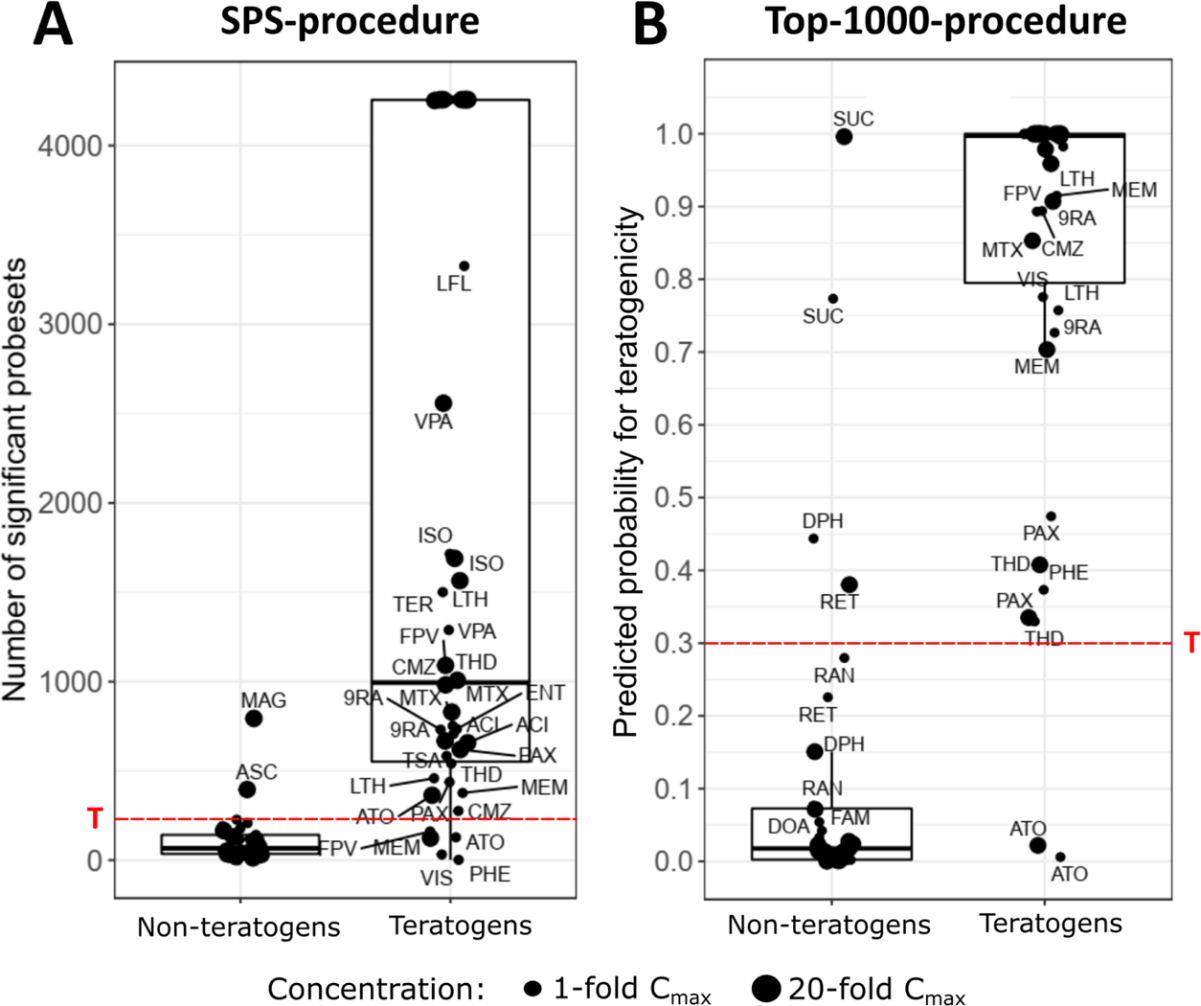
Classification of the teratogenic and non-teratogenic compounds by (A) the SPS-procedure, a method based on the number of significantly deregulated probe sets and (B) the top-1000-procedure, a penalized logistic regression-based technique using the 1000 probe sets with the highest variability. (A) SPS-procedure. The number of SPS is given in the *y*-axis, and the *x*-axis marks non-teratogens and teratogens (compound abbreviations are explained in Table 1). The threshold *T* at 228 SPS separates negative and positive in vitro test results for the calculation of accuracy, sensitivity, and specificity. Key rules of the SPS-procedure: The number of SPS is the sum of up- and downregulated probe sets with an FDR-adjusted *p*-value < 0.05 and an absolute fold change > 2 compared to control conditions. The number of SPS in cytotoxic conditions corresponds to entinostat at 20-fold *C*_max_ (i.e. 4252 SPS) which showed the highest number of SPS across all samples. (B) Top-1000-procedure. The predicted probability for teratogenicity is given on the *y*-axis, and the *x*-axis marks non-teratogens and teratogens. The threshold *T* at a predicted probability of 0.3 separates negative and positive in vitro test results for the calculation of accuracy, sensitivity, and specificity. Key rules of the top-1000-procedure: calculation of the probability was based on a leave-one-out-cross-validation-algorithm and the 1000 probe sets with the highest variance. This resulted in 34 unique classifiers (i.e., one for each non-cytotoxic compound) for which a total of 1160 different probe sets had to be considered because of a strong overlap between the underlying probe sets (Figure S1A). GO over-representation analysis on the 1160 PS (Figure S1B) showed similarities to the GO groups of overlapping genes (Figure 5). Lists of the overall 1160 probe sets and the 1000 probe sets of each classifier are given in the Supporting Information “Classifer”. Cytotoxic conditions were considered to be 100% positive (predicted probability, 1.0).

Two techniques were applied to classify the test compounds based on gene expression and cytotoxicity. First, the number of SPS was used and a compound was classified as test-positive or test-negative if this number was above or below a specific threshold; for simplicity, this technique was further named “SPS-procedure”. Accuracy was highest using a threshold of 228 significantly deregulated probe sets (SPS), which was then applied in subsequent analyses (Figure 4A). With the exception of ASC and MAG (abbreviations defined in Table 1), the SPS-procedure correctly classified the non-teratogens as test-negative (true negative); the teratogens were test-positive (true positive) with the exception of ATO, FPV, MEM, PHE, and VIS that were test-negative (false negative). Cytotoxic conditions were considered as a positive test result and were integrated into the classification procedure by assigning them with the highest observed number of SPS across all samples, that is, 4252 SPS as observed for entinostat at 20-fold *C*_max_ (Table 2).

Second, penalized logistic regression was performed based on the 1000 probe sets with highest variance with leave-one-out cross-validation, further named “top-1000-procedure”. Using this approach, all teratogens, except ATO, were correctly classified as test-positives together; the non-teratogens were test-negative with the exception of SUC, DPH, and RET that were false positive (Figure 4B). A comprehensive overview of the predicted probabilities of all compounds and the classification results (true/false positive; true/false negative) are given in Tables S5 and S6 in the Supporting Information, respectively.

We next investigated which of the two procedures—SPS or top-1000—was superior at distinguishing teratogenic from non-teratogenic compounds. In addition, we also examined if 1-fold or 20-fold *C*_max_ should be considered and whether information on cytotoxicity should be included with gene expression for classification. The top-1000-procedure led to higher values for the AUC, accuracy, and sensitivity compared to the classification using the number of SPS (Table 3). Moreover, classification of gene expression data obtained with the 1-fold *C*_max_ concentration resulted in slightly higher values for the AUC, accuracy, and sensitivity compared to the 20-fold *C*_max_ for both procedures. Cytotoxicity alone allowed classification with relatively low metrics values. Combined analysis of cytotoxicity and gene expression consistently increased the AUC, accuracy, and sensitivity of both the SPS- and the top-1000-procedure, compared to the analysis of gene expression (SPS- or top-1000) alone. The highest values for AUC, accuracy, and sensitivity of 0.96, 0.92, and 0.96, respectively, were obtained for the top-1000-procedure based on gene expression data combined with cytotoxicity for the 1-fold *C*_max_ concentration. However, the specificity was only 0.88; whereas the SPS-procedure consistently reached the highest specificity of 1 for the 1-fold *C*_max_. When cytotoxicity alone was considered, a specificity of 1 was obtained, but with very low sensitivity, accuracy, and AUC.

**Table 3:** Performance Metrics of the SPS-Procedure and the Top-1000-Procedure

			SPS-procedure		top-1000-procedure	
metric	*C*_max_	cytotoxicity only	gene expression only	cytotoxicity and gene expression	gene expression only	cytotoxicity and gene expression
AUC	1-fold	0.61	0.87	0.90	0.94	0.96
	20-fold^a^	0.63	0.86	0.90	0.93	0.95
accuracy	1-fold	0.54	0.77	0.90	0.79	0.92
	20-fold^a^	0.56	0.72	0.87	0.77	0.92
sensitivity	1-fold	0.22	0.61	0.83	0.74	0.96
	20-fold^a^	0.26	0.61	0.87	0.70	0.96
specificity	1-fold	1.00	1.00	1.00	0.88	0.88
	20-fold^a^	1.00	0.88	0.88	0.88	0.88

a Including 10-fold *C*_max_ carbamazepine, 1.67-fold *C*_max_ valproic acid (VPA), and 1-fold *C*_max_ samples of leflunomide, phenytoin, teriflunomide, and vismodegib. Cytotoxicity only: Only cytotoxicity data were considered for the calculation of the metrics, that is, cytotoxic conditions were considered as positive and non-cytotoxic conditions as negative test results. Gene expression only: only gene expression data were considered for the calculation of the metrics. Cytotoxicity and gene expression: all data for cytotoxicity as well as for gene expression were considered for the calculation of the metrics. AUC: for each possible cutoff used as threshold, predictions were made for each of the conditions, based on which sensitivity and specificity were calculated. The ROC curve was obtained by plotting all pairs of (1-specificity) and sensitivity against each other. The AUC was determined as the area under this ROC curve. Accuracy: ratio of correct predictions ((true negatives and positives)/(true and false negatives and positives)) (Table S6). Sensitivity: ratio of detected teratogens (true positives/(false negatives + true positives)) (Table S6). Specificity: ratio of detected non-teratogens (true negatives/(true negatives + false positives)) (Table S6).

### Biological Interpretation of Gene Expression Changes by Teratogens and Non-teratogens

**Figure 5:**
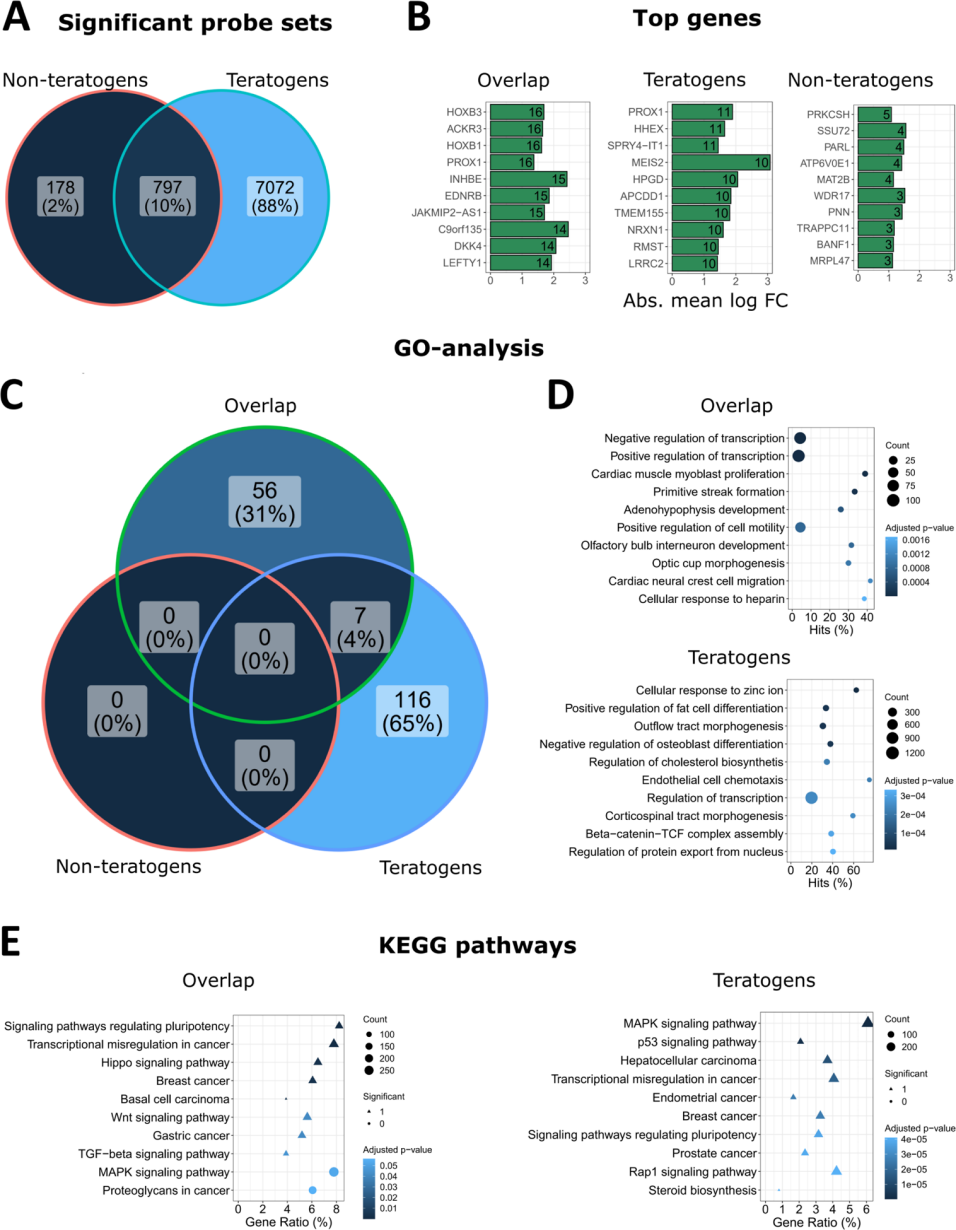
Biological interpretation of genes differentially expressed after exposure of hiPSCs to teratogens and non-teratogens at 1-fold *C*_max_. (A) Numbers of SPS (log2 fold change > 1; adjusted *p*-value < 0.05) induced by non-teratogens and teratogens at the plasma peak concentration (1-fold *C*_max_). (B) Top genes in the gene sets of the overlap, teratogens, and non-teratogens from (A). The numbers in the bars indicate the number of compounds that deregulated the specific genes. All differential genes are given in the Supporting Information “Top genes”. (C) Numbers of significantly (adj. *p*-value < 0.05) over-represented GO groups in the overlap, teratogen, and non-teratogen gene sets. (D) Ten GO groups with the lowest adj. *p*-values in the overlap and teratogen gene sets. No significant GO groups were obtained for the non-teratogen gene set. The names of the GO groups were shortened. Full names and complete GO group lists can be found in the Supporting Information “GO analysis”. “Count”: number of significant genes from (A) linked to the GO group. “Hits”: percentage of significant genes compared to all genes assigned to the GO group. (E) KEGG pathway enrichment analyses of the overlap and teratogen gene sets. The ten KEGG pathways with the lowest adj. *p*-values are given. No significant KEGG pathways were obtained for the non-teratogen gene set. Full names and complete KEGG-pathway lists are given in the Supporting Information “KEGG pathways”. “Count”: number of significant genes from (A) linked to the KEGG pathway. “Gene Ratio”: percentage of significant genes associated to the pathway compared to the number of all significant genes associated to any pathway.

To study the biological significance of genome-wide expression changes, we first investigated if teratogens and non-teratogens influence the expression of similar or different sets of genes. For this purpose, we considered probe sets that were deregulated by the 23 tested teratogens, as well as those altered by the 16 non-teratogens. At the plasma peak concentration (1-fold *C*_max_), a higher number of probe sets was significantly deregulated by the teratogens (*n* = 7869) compared to those influenced by the non-teratogens (*n* = 975) (Figure 5A). Interestingly, a large fraction of the probe sets deregulated by the non-teratogens (797 of the 975 probe sets) overlapped with those deregulated by the teratogens (Figure 5A). A similar scenario was also observed when the up- and downregulated probe sets were separately analyzed (Figures S2 and S3), and for the data set obtained with higher concentrations of the test compounds (Figures S3–S5).

To characterize biological functions, we differentiated three gene sets: first, genes that overlap upon treatment with both teratogens and non-teratogens, for simplicity further named “overlap gene set”; second, genes exclusively altered by the teratogens, further named “teratogen gene set”; and third, genes exclusively influenced by the non-teratogens, accordingly named “non-teratogen gene set”. Initially, we focused on the probe sets that were significantly deregulated by the highest number of test compounds (Figure 5B, Supporting Information “Top genes”). In the overlap gene set, HOXB3, ACKR3, HOXB1, and PROX1 were most frequently affected and significantly deregulated by 16 compounds. The genes that were most frequently deregulated in the teratogen gene set were PROX1, HHEX, and SPR4-IT1. They were influenced by 11 compounds. It may appear surprising that PROX1 occurs as a top gene in both the teratogen and in the overlap gene sets, but this may be due to the probe set-based analysis, where different probe sets in both gene groups were annotated to the same gene, PROX1. The gene most frequently altered in the non-teratogen gene set (PRKCSH) was influenced by only five compounds. The genes in the overlap and in the teratogen gene set suggest functions in development and differentiation. For example, HOXB3, ACKR3, HOXB1, and PROX1—the top genes in the overlap gene set—are all transcription factors involved in the development of several tissues.^33−36^ Furthermore, PROX1 and HHEX, the genes most frequently altered in the teratogen gene set, are also known to be involved in developmental processes,^36,37^ while the cancer-associated long non-coding RNA SPR4-IT1 influences differentiation- and proliferation-associated genes.^38^ In contrast, the non-teratogen gene set seems to be associated with a variety of processes, for example, PRKCSH represents the beta-subunit of glucosidase II in the endoplasmic reticulum,^39^ SSU72 is a phosphatase,^40^ and PARL encodes a mitochondrial protease,^41^ suggesting no specific biological motif.

To address the biological motif of expression changes by an unbiased method, an over-representation analysis of GO groups was performed for the three gene sets (Figure 5C, Supporting Information “GO analysis”). The overlap and teratogen gene sets resulted in 63 and 123 significantly over-represented GO groups, respectively (Figure 5C, Supporting Information “GO analysis”). In contrast, probe sets exclusively deregulated by the non-teratogens did not result in any significantly over-represented GO group. For both the teratogen and the overlap gene sets, developmental processes of several tissues were significantly over-represented (Figure 5D), including cardiac muscle, primitive streak, adenohypophysis, olfactory bulb, optic cup, neural crest (overlap gene set) and fat cell, outflow tract, osteoblast, and corticospinal tract (teratogen gene set). Moreover, other general motifs, such as regulation of transcription, were obtained in both gene sets. Analysis of enriched KEGG pathways also identified identical or similar pathways in the overlap and teratogen gene sets. Examples include signaling pathways regulating pluripotency, MAPK signaling pathways, as well as several cancer-associated pathways (Figure 5E, Supporting Information “KEGG pathways”). As for the GO groups, no significant over-representation of KEGG pathways was obtained for the gene set of non-teratogens.

## Discussion

The reliable identification of developmental toxicants by an in vitro test is important, because conventional animal testing, for example, using a 2-generation reproduction study, is labor- and cost-intensive and requires large numbers of animals.^42,43^ In the present study, we tested a set of 23 teratogens and 16 non-teratogens by gene expression profiling at concentrations of 1-fold and 20-fold *C*_max_ in the hiPSC-derived UKK2-test. Using a penalized logistic regression procedure (top-1000-procedure) at 1-fold *C*_max_, together with information on whether cytotoxicity occurs, classification was possible with an AUC of 0.96, an accuracy of 0.92, a sensitivity of 0.96, and a specificity of 0.88. These performance metrics were unexpectedly favorable, considering that a hiPSC-based system was used, where cardiac differentiation was initiated. Most of the tested teratogens were not reported to specifically affect cardiac development but are known to disturb other aspects of embryo-fetal development, such as limb deformations by thalidomide,^1^ spina bifida by valproic acid,^44^ or developmental neurotoxicity due to methylmercury exposure.^45^ A possible explanation why these compounds were tested positive in the UKK2-test may be that the here-applied hiPSCs activate numerous gene regulatory networks during differentiation that overlap with those of other embryo-fetal developmental processes. Thus, even if a test does not recapitulate a specific developmental process like limb development, it may nevertheless show gene expression changes after exposure to, for example, thalidomide at in vivo relevant concentrations.

The goal of the present study was to answer three basic questions on how a transcriptomics-based developmental in vitro test should be performed. First, we observed that a penalized logistic regression procedure (top-1000-procedure) with leave-one-out cross-validation based on the 1000 probe sets with the highest variance allows classification with a higher AUC and accuracy than just using the number of differential genes (SPS-procedure). However, the situation is complex because the top-1000-procedure led to higher sensitivity, while the SPS-procedure resulted in higher specificity. It must be noted, however, that the SPS-procedure was not cross-validated with a leave-one-out-approach like the top-1000-procedure. If such an approach was performed, only the value of the threshold would have changed in each fold of the cross-validation. Because the measured numbers of the SPS-procedure would not have been altered, the resulting changes in accuracy, sensitivity, and specificity would have been small, with metrics close to the non-cross-validated metrics. Thus, future studies should further consider both approaches.

A second question was if a test should be considered as positive if cytotoxicity occurs. The results clearly show that higher metrics are obtained when information on cytotoxicity is included into the classification procedure. Cytotoxicity alone led to an AUC of 0.61 and 0.63 for 1-fold and 20-fold *C*_max_, respectively, which is better than a random result, but much worse when compared to the procedure that includes gene expression. A specific cytotoxicity test, for example, based on mitochondrial activity, was not performed because cytotoxicity at the therapeutic *C*_max_ was not expected and thus not considered when the experiments were initially designed. This omission is a limitation of the present study, as the results clearly indicate that a sensitive cytotoxicity test, such as the CellTiter-Blue Cell Viability Assay,^16,46^ would improve the metrics and should be included in future studies. However, it should also be considered that most of the true positive test results were due to gene expression alterations at non-cytotoxic concentrations and only five (at 1-fold *C*_max_) and six (at 20-fold *C*_max_) of the 23 teratogens showed cytotoxicity.

The third question addressed in this study was if classification is more precise at 20-fold *C*_max_ than at 1-fold *C*_max_. No major differences in the metrics were obtained between both concentrations, rather the values were slightly higher for 1-fold *C*_max_ than for 20-fold *C*_max_. This result was surprising because previous classification studies on hepato- and nephrotoxicity reported better classification for at least 20-fold higher concentrations than the *C*_max_.^16,17^ This discrepancy between developmental and liver, as well as kidney toxicity may be explained by the fact that a relatively high fraction of hepato- or nephrotoxic compounds require metabolic activation.^48,43^ Because metabolic activities of cultivated cells are usually lower compared to the in vivo situation, higher test compound concentrations may be required in vitro to induce similar toxic effects as seen in vivo. In contrast, metabolism may be less critical for the here-analyzed developmental toxicants, which may explain the favorable result with 1-fold *C*_max_. Nevertheless, concentration-dependent testing, including lower concentrations than the *C*_max_, may provide further insights in future studies.

Finally, the misclassifications of some compounds should be discussed as they may reveal limitations of the test system, which could be addressed in upcoming experiments. The compounds MAG and ASC at 20-fold *C*_max_ in the SPS-procedure and RET at 20-fold *C*_max_ in the top-1000-procedure were false positives. However, RET is known to be teratogenic at high doses,^49^ and overdoses of ASC and MAG have been shown to cause adverse effects,^50,51^ indicating that the high concentrations used in vitro may also compromise differentiating cells in vivo. In contrast, the misclassification of SUC, DPH, and ATO by the top-1000-procedure shows that the test still has to be improved.

In previous studies, we observed that different classes of teratogens, such as HDAC inhibitors and mercurials can be differentiated based on their gene expression profiles.^9,10^ In these previous studies, concentrations were tested that were based on cytotoxicity so that all compounds were compared with identical factors below cytotoxic thresholds. In contrast, the present study was based on maximal plasma concentrations (*C*_max_) that result after specific doses of drugs that either cause or not cause an increased risk of teratogenicity if used during pregnancy. As expected, the non-teratogens caused expression changes in a much lower number of genes compared to the teratogens. Surprisingly, a large fraction of the genes (approximately 82%) altered by the non-teratogens overlapped with the genes deregulated by teratogens. Moreover, similar GO groups and KEGG pathways associated with developmental processes were affected. These findings suggest that non-teratogens can also disturb the differentiation of the here-applied hiPSC if high enough concentrations are applied. Therefore, the identification of in vivo relevant concentrations for the exposure of the stem cells represents a very important step of the evaluation procedure.

The analysis of GO groups and KEGG pathways of genes differentially expressed after test compound exposure of the here-applied in vitro cardiomyogenic protocol UKK2^18,19^ did not only result in over-representation of genes associated with cardiac muscle development but also with the differentiation of a much larger set of tissues, including primitive streak, adenohypophysis, olfactory bulb, optic cup, neural crest, fat cells, and osteoblasts. Moreover, genes associated with a large set of signaling pathways were enriched, such as Wnt, MAK kinase, P53, Rap1, Hippo, and TGF-beta signaling. Although further research is required to understand the biological mechanism underlying these processes, the broad spectrum of involved pathways and GO groups may be advantageous if one aims for a test that comprehensively identifies human teratogens.

A previous study presented a metabolic biomarker-based in vitro test with human embryonic stem cells for developmental toxicity screening, where the amino acids ornithine and cystine in the culture medium were identified as biomarkers.^52,53^ This test was reported to identify developmental toxicants with an accuracy of 77%, a sensitivity of 57%, and a specificity of 100%. In our subsequent work, it may be worthwhile to test whether the combination of these metabolic biomarkers together with gene expression profiles improves performance measures. A further perspective is to include stem cell-based assays that recapitulate other developmental steps in addition to the UKK2-test. For example, the UKN1-test models neuroectodermal induction resulting in neural ectodermal progenitor cells.^47,54,55^ Thus, subsequent important steps will be to integrate additional cell systems that recapitulate complementary developmental processes and to include further readouts in order to study if these altogether improve accuracy.

In the current work, we present two classification procedures, the SPS-procedure and the top-1000-procedure, rather than specific classifiers. An advantage is that such classification procedures can be applied to any data set with other hiPSC lines, differentiation protocols or compounds. In contrast, a specific classifier represents a fixed set of genes and algorithms, which could be established with the available data, but may result in misleading conclusions based on the current state of research. It cannot, for example, be excluded that using other hiPSC lines, differentiation protocols or compounds will lead to different genes with the highest variance, and consequently to other top-1000-classifiers, or that, for example, the threshold of the gene number (here: *n* = 228) will be different for the SPS-classifier. Thus, it appears more appropriate to test and compare classification procedures in the present data set, which to our knowledge is the first gene expression-based classification study of teratogens using in vivo relevant (1-fold *C*_max_) test compound concentrations. Only when data on more compounds, hiPSC lines, and protocols are available, will it then be worthwhile to address if a universally valid, fixed classifier can be identified.

In conclusion, we established UKK2, a transcriptomics- and hiPSC-based test, which identifies developmental toxicants with high in vivo concordance. Even in its present state, the here-established assay that requires only a 24 h incubation period with test compounds may be useful as part of a battery of tests that are performed during the discovery phase in drug development.

## Supporting Information Available

Mechanisms of action and teratogenic evidence of teratogenic compounds; compounds and their human relevant plasma or blood concentrations at therapeutic doses; placental transfer of non-teratogenic compounds; predicted probabilities for teratogenicity; classification of the in vitro test results in the two procedures; overlap analysis and GO analysis of the 1160 probe sets of the 34 classifiers of the top-1000-procedure; biological interpretation of genes differentially expressed after exposure of hiPSC to teratogens and non-teratogens; and volcano plots of all teratogens and non-teratogens (PDF)
Classifier (XLSX)
Top genes (XLSX)
GO analysis (XLSX)
KEGG pathways (XLSX)

## Abbreviations

hiPSC: human induced pluripotent stem cell
*C*_max_: maximal plasma concentration
PS: probe set
SPS: significant probe set
AUC: area under the (receiver operator characteristic) curve
GO: Gene Ontology
KEGG: Kyoto Encyclopedia of Genes and Genomes
