# Supplementary material for: Classification of Developmental Toxicants in a Human iPSC Transcriptomics-Based Test
Source: Chem Res Toxicol. 2022 Apr 13;35(5):760–73. doi: 10.1021/acs.chemrestox.1c00392 (PMC9377669; doi:10.1021/acs.chemrestox.1c00392)
Supplement: Supplementary file 1 — tx1c00392_si_001.pdf [file tx1c00392_si_001.pdf]

SUPPORTING INFORMATION

Classification of developmental toxicants in a human iPSC transcriptomics-based test

Anna Cherianidou,[†,‖] Florian Seidel,[§,‖] Franziska Kappenberg,[‡,‖] Nadine Dreser,[Ⅎ] Jonathan Blum,[Ⅎ] Tanja Waldmann,[Π] Nils Blüthgen,[Σ,Δ] Johannes Meisig,[Σ,Δ] Katrin Madjar,[‡] Margit Henry,[†,¢] Tamara Rotshteyn,[†,¢] Rosemarie Marchan,[§] Karolina Edlund,[§] Marcel Leist,[Ⅎ,⊥] Jörg Rahnenführer,[‡,⊥] Agapios Sachinidis,[†,¢,⊥] Jan G. Hengstler*[,§,⊥]

[‖]A.C., F.S. and F.K. have equal first authorship contribution

[⊥]M.L., J.R., A.S. and J.G.H. have equal senior authorship contribution

*corresponding author (Hengstler@ifado.de)

Affiliations

[†]University of Cologne, Faculty of Medicine and University Hospital Cologne, Center for Physiology, Working Group Sachinidis, Robert-Koch-Str. 39, 50931 Cologne, Germany

[§]Leibniz Research Centre for Working Environment and Human Factors at the Technical University of Dortmund (IfADo), Ardeystrasse 67, 44139 Dortmund, Germany

[‡]Department of Statistics, TU Dortmund University, Vogelpothsweg 87, 44227 Dortmund, Germany

[Ⅎ]In Vitro Toxicology and Biomedicine, Department of Biology, University of Konstanz, Universitätsstr. 10, PO, Box M657, 78457 Konstanz, Germany

[Π]Department of Advanced Cell Systems, trenzyme GmbH, Byk-Gulden-Str. 2, 78467 Konstanz, Germany

[Σ]Institute of Pathology, Charité-Universitätsmedizin Berlin, Chariteplatz 1, 10117 Berlin, Germany

[Δ]IRI Life Sciences, Humboldt Universität zu Berlin, Philippstraße 13, Haus 18, 10115 Berlin, Germany

[¢]Center for Molecular Medicine Cologne (CMMC), University of Cologne, 50931 Cologne, Germany

Table of Contents

Table S 1: Mechanisms of action and teratogenic evidence of teratogenic compounds……………….S3

Table S 2: Non-teratogenic compounds and their human relevant plasma or blood concentrations at therapeutic doses……………………………………………………………………………………….S5

Table S 3: Teratogenic compounds and their human relevant plasma or blood concentrations at therapeutic doses……………………………………………………………………………………….S8

Table S 4: Placental transfer of non-teratogenic compounds……………………………………………………..S11

Table S 5: Predicted probabilities for teratogenicity…………………………………………………………………..S12

Table S 6: Classification of the in vitro test results in the two procedures……………………………………S13

Figure S1: Overlap-analysis (A) and GO-analysis (B) of the 1160 probe sets of the 34 classifiers of the top-1000-procedure………...S14

Figures S2-S6: Biological interpretation of genes differentially expressed after exposure of hiPSC to teratogens and non-teratogens………………………………………………………………………………………S15

References………S21

Volcano-plots of non-teratogens (alphabetically sorted)……………………………………………………………S27

Volcano-plots of teratogens (alphabetically sorted)…………………………………………………………………..S59

Table S 1: Mechanisms of action and teratogenic evidence of teratogenic compounds

Teratogenic compound	Mechanism of action (MoA)	Evidence for teratogenicity
9-cis-Retinoic acid (Alitretinoin)	Binds at and acts over retinoic acid receptors (RARs) and retinoid x receptors (RXRs) [1–3]	Short trunk, limb reduction defects, ectrodactyly, cleftpalate, micrognathia, and other craniofacial defects in mice [4]
Acitretin	Activates, but does not bind to RARs [5]. Binding to RXRs is not known	Retinoid acid related defects (see isotretinoin) in few human case reports and animal studies [6]
Actinomycin D	Binds to DNA and inactivates RNA synthesis [7]	Malformations of CNS, viscera and skeleton in animals (rat, hamster, rabbit) [8]. Human data do not show evidence for teratogenicity [6]
Atorvastatin	3-hydroxy-3-methylglutaryl-coenzyme A (HMG-CoA) reductase inhibitor [9]	CNS, skeleton and cardiovascular malformations in few human and animal studies in which different statins were used. The biochemical mechanism indicates a potential embryo-fetal risk. However, in a lot of studies, statins didn't induce malformations [6;9]
Carbamazepine	Blocks voltage-gated sodium channels [10]	Association with an increased incidence of major and minor malformations, including neural tube defects (NTDs), cardiovascular and urinary tract defects, and cleft palate, supported by different human studies and case reports [6;11]
Doxorubicin	Among others, interaction with and alteration of DNA and related proteins; production of free radicals and reactive oxygen-species (ROS) [12]	Structural anomalies and death in rats and rabbits; association with similar outcomes in humans after exposure during organogenesis [6]
Entinostat (MS-275)	HDACi [13]	No data available, refer to other HDACis
Favipiravir	Selective inhibitor of the RNA-dependent RNA polymerase of influenza virus [14]	Teratogenic and embryotoxic effects in animal studies. No human data available [15]
Isotretinoin	Binds at and acts over retinoid acid receptors (RARs) but not retinoid X receptors (RXRs) [1–3]	Craniofacial, CNS, cardiovascular and thymic defects, well-documented in animal studies and humans [16;17;6]
Leflunomide	Is metabolized to its active metabolite teriflunomide; inhibits dihydroorotate dehydrogenase (DHODH), a key player in de novo pyrimidine synthesis [18]	Teratogenic in animals at human relevant doses. Human data do not show evidence for teratogenicity [6]
Lithium (chloride)	Many cellular targets, e.g. inhibition of glycogen synthase kinase 3 (GSK-3) [19]	Although evidence is weak, lithium may lead to an increased incidence of cardiovascular defects and Ebstein's anomaly [6]
Methotrexate	Multiple targets; best known is its antifolate effect as dihydrofolate reductase inhibitor [20]	Multiple defects in animals and humans, e.g. limb malformations, CNS damages and craniofacial defects [6;21;22]
Methylmercury	Exact mechanism is unknown; among others, the mostly hypothesized is the production of ROS [23–25]	Malformations in animals, neural and neurodevelopmental defects in animals and humans [26–29]
Panobinostat	HDACi [30]	Teratogenic, embryo-fetal toxic and mutagenic in animals. Human data are not available [6;30]
Paroxetine	Selective serotonin reuptake inhibitor (SSRI) [31]	Different major and minor malformations and defects in some human studies and case reports, e.g. cardiac defects; withdrawal symptoms in neonates [6]

Teratogenic compound	Mechanism of action (MoA)	Evidence for teratogenicity
Phenytoin (5,5-Diphenylhydantoin)	Blocks voltage-gated sodium channels [10]	Major and minor congenital abnormalities like craniofacial and limb defects; fetal hydantoin syndrome (FHS); association with neurodevelopmental defects; evidence is supported by different human studies and case reports [6]
Teriflunomide (A-771726)	Active metabolite of leflunomide; inhibits dihydroorotate dehydrogenase (DHODH), a key player in de novo pyrimidine synthesis [18]	Teratogenic in animals at human relevant doses. Human data do not show evidence for teratogenicity [6]
Thalidomide	The exact mechanism is unknown, but it has multiple possible targets; most prominent are its antiangiogenic properties [32]	Severe defects of limbs, skeleton, head & face, CNS and organ system in numerous human cases [6;32]
Trichostatin A (TSA)	HDACi [33]	No data available, refer to other HDACis
Valproic acid (VPA)	Multiple possible targets and actions: Activator of glutamic acid decarboxylase (GAD), voltage-gated sodium channel blocker, reduction of folic acid levels, HDACi [34;10]	Neural tube defects (NTDs), spina bifida, fetal valproate syndrome, defects of the head, face, limbs, urogenital tract and neurodevelopment in animals and humans [6;34]
Vinblastine	Inhibits mitosis and arrests cell cycle by affecting microtubule dynamics [35]	Evidence is weak: Malformations in hamster embryos in one study [36], unspecified details in other animal studies, no data on human pregnancies [6]
Vismodegib	Hedgehog pathway inhibitor [37]	Congenital malformations in rats at human relevant doses. No human data available [38;37]
Vorinostat	HDACi [39]	Teratogenic in animals at human relevant doses. Human data are not available [6;39]

Table S 2: Non-teratogenic compounds and their human relevant plasma or blood concentrations at therapeutic doses. *Common therapeutic doses and dosing schemes were collected from www.drugs.com if not stated otherwise. Routes of application were as follows: PO= Peroral, IV=Intravenously, IM=Intramuscular or subcutaneously. RDA = Recommended daily allowance.*

Non-teratogenic compound	Common therapeutic doses and dosing schemes in humans	Clinical study design, dosing scheme and other resources of information	Plasma peak or blood concentrations	Reference
Ampicillin	Endocarditis: 2 g IV every 4 h<br><br>Meningitis: 150 to 200 mg/kg/day IV in equally divided doses every 3 to 4 h<br><br>Other infections: 500 mg PO, IM, IV every 6 h	a) 400 mg/kg/day in six doses IV, healthy children (n = 17)<br>b) 1.5 g sultamicillin (containing the equivalent of 882 mg ampicillin) with probenecid (1.0 g) (n=8, men)	a) After 30 min: 37.3 ± 19 µg/ml (107 ± 55 µM)<br>After 3 h: 11 ± 10.2 µg/ml (32 ± 29 µM)<br>b) 23.1 µg/ml (66 µM)	a) [40]<br>b) [41]
Ascorbic acid	RDA: 80 mg<br><br>50 - 200 mg/day PO, IM, IV, or subcutaneously as dietary supplement	a) 30 - 2500 mg PO daily (n= 17, 7 men, 10 women)<br>b) 30 - 2500 mg PO (n=7, men)	a) 200 mg: 90 µM (predicted)<br>1250 mg: 187 µM (predicted)<br>3000 mg: 206 µM (predicted)<br>b) 60 mg: 20 µM (steady-state)<br>200 mg: 60 µM (steady-state)<br>2500 mg: 90 µM (steady-state)	a) [42]<br>b) [43]
Buspirone	15-60 mg in divided doses	a) 20 mg PO<br>b) 20mg PO, healthy men (n=12)<br>c) 30mg PO (n=9, healthy adults)	a) 2.5 ng/ml (5.9 nM)<br>b) 1.15 ± 0.77ng/ml (0.9 - 4.55 nM)<br>c) 6.6 ± 3.7 ng/ml (15.6 - 24.4 nM)	a) [44]<br>b) [45]<br>c) [46]
Chlor-pheniramine	4 mg PO every 4 to 6 hours	a) 8 mg chlorpheniramine maleate PO (4 male, 1 female healthy adults)<br>b) (+)chlorpheniramine 2 mg and 6 mg PO, 12 healthy men	a) 11.9 - 35.6 ng/ml (30.4 nM - 91 nM), mean 17.9 ng/ml (46 nM)<br>b) 2mg: 3.4–7.4 ng/ml, mean 5.0 ± 1.1 ng/ml<br>6 mg: 2.0–14.3 ng/ml, mean 7.9 ± 3.2 ng/ml	a) [47]<br>b) [48]
Dextro-methorphan	30-120 mg / day	a) 30 mg (dextrometorphan hbr) (n=9)<br>b) 30-60 mg PO, with or without quinidine (n=121, healthy adults)	a) 0.12 ± 0.03 µM<br>b) max. 7.7 ± 7.0 ng/ml (28 ± 26 nM)<br>with quinidine: max. 232 ± 96 ng/ml (856 ± 354 nM)	a) [49]<br>b) [50]
Diphen-hydramine	12.5 - 76 mg / dose, PO, IV, IM, max. 400 mg / day	a) Single 50 mg oral dose, healthy women (n=25)<br>b) 50 mg p.o once (healthy humans)<br>c) 6.25 - 50 mg PO (allergic children)<br>d) Overdose (humans)	a) 0.3 µM (74 ng/ml)<br>b) 37 - 83 ng/ml<br>c) 48 - 93 ng/ml<br>d) 0.1 – 4.7 µg/ml (lethal)	a) [51]<br>b) [52]<br>c) [53]<br>d) [54]
Doxylamine	10-40 mg daily	a) 25 mg PO once (n=25, healthy women)<br>b) 12.5 mg PO once (n=12, 3 men, 9 women, healthy)<br>c) 25 mg PO once (n=12, 3 men, 9 women, healthy)	a) 0.38 µM<br>b) 0.23 µM<br>c) 0.46 µM	a) [51]<br>b-c) [55]
Famotidine	20-40 mg PO daily	a) 40 mg PO (n=7, healthy adults)<br>b) 20-40 mg, different studies<br>c) 20 mg IM, pregnant women (n=34)	a) 17 to 139 ng/ml (50.3 - 411.9 nM)<br>b)20 mg: 4 - 137 ng/ml (11.8 - 406 nM), mean 71 ng/ml (210 nM)<br>40 mg: 15 - 358 ng/ml (44.4 - 1060 nM) , mean 132 ng/ml (391 nM)<br>c) Maternal: approx. 140 ng/ml (418 nM)<br>Fetal: approx. 90 ng/ml (267 nM)	a) [56]<br>b) [57]<br>c) [58]

Non-teratogenic compound	Common therapeutic doses and dosing schemes in humans	Clinical study design, dosing scheme and other resources of information	Plasma peak or blood concentrations	Reference
Folic acid	400 µg daily<br>500 µg during Lactation<br>600 µg during Pregnancy	a) 1.1 mg or 5 mg PO once (n=6, non-pregnant healthy women)<br>b) 400 µg PO daily during whole pregnancy (n=10, pregnant women)	a) Baseline: 0.011 – 0.034 µM (5 to 15 ng/mL)<br>1.1 mg: 0.014 µM (6.2 +/- 2.2 ng/mL) steady-state (estimated)<br>5.0 mg: 0.094 µM (41.6 +/- 11.3 ng/mL) steady-state (estimated)<br>b) Umbilical cord blood: Up to 90.5 nM total folate	a) [59]<br>b) [60]
Levothyroxine	Max. 200 - 300 µg daily	a) 600 µg PO, 18 men and 18 women<br>b) 54 - 300 µg 2 men, 4 women<br>c) 600 µg (n=84)<br>d) 115.2 ± 38.5 µg/day (n=10, 9 female, 1 male)<br>e) Baseline, neonates cord blood (n=40)	a) 76.64 ± 16.48 ng/ml (99 nM ± 21.2 nM)<br>b) max. 1.25±0.51 ng/dl<br>c) 71.4 ± 16.0 ng/ml<br>d) 106 ± 22 nM<br>e) Cord blood: 35 - 70 nM	a) [61]<br>b) [62]<br>c) [63]<br>d) [64]<br>e) [65]
Liothyronine	25-75 µg daily	a) 40.3 ± 11.3 µg/day (n=10, 9 female, 1 male)<br>b) 50 µg PO, (n=12, 4 females and 8 males)<br>c) 50 - 300 µg / day (n=15, pregnant women)	a) 2.84 ± 1.54 nM<br>b) 346 ng/dl (5.3 nM)<br>c) Baseline: Maternal: 4.1 ± 1.1 µg/l (6.3 ± 1.7 nM)<br>Baseline Fetal: 3.2 ± 1.3 µg/l<br>Treated: Maternal: 8.5 ± 1.7 µg/l<br>Treated: Fetal: 5.6 ± 1.7 µg/l	a) [64]<br>b) [66]<br>c) [67]
Magnesium (chloride)	As dietary supplement / RDA: Up to 400 mg (16.5 mmol) daily for pregnant women<br><br>Constipation & Dyspepsia: Up to 4.8 g daily over max. 7 days (as MgOH)<br><br>Hypomagnesemia: Up to 5 g IV in 3 hours (as $MgSO_4$)<br><br>Pre-eclampsia: Max. daily dose: 40 g IV (as $MgSO_4$)	a) 4 g loading dose + 2 g/h maintenance dose IV (n=111, pre-eclampsia treatment of pregnant women<br>b) MgOH 3 x 360 mg PO (45 mmol Mg) (n=10, healthy men)<br>$MgSO_4$ 2 g IV (8 mmol Mg) (n=10, healthy men)<br>c) 12 and 24 mmol Mg PO (n=8, healthy men)<br>4 and 8 mmol Mg IV (n=8, healthy men)	a) 7.2 mg/dl (3 mM) steady-state (Baseline: 2.0 mg/dl (0.82 mM))<br>b) MgOH: 0.95 mM<br>$MgSO_4$: 1.2 mM<br>c) Baseline: 0.78 - 0.83 mM<br>12 mmol PO: 3.1 % increase<br>24 mmol PO: 4.6 % increase<br>4 mmol IV: 9.5 % increase<br>8 mmol IV: 16.1 % increase	a) [68]<br>b) [69]<br>c) [70]
Methicillin	Adults: 1 g IM every 4 h<br>Infants: 0.5 g IM every 4 h<br><br>[71]	a) 2 g IV once, (n=5, male and female adults)<br>b) 500 mg IV, pregnant women (n=105)	a) Immediately after administration: ~ 140 µM<br>2 h after injection: 13-20 µg/ml (34 - 53 µM)<br>b) 30 min after injection: 10.5 ± 4.3 µg/ml<br>2 h after injection: 3.2 ± 1.9 µg/ml	a) [72]<br>b) [73]
Ranitidine	150 - 300 mg PO daily	a) 150 mg<br>b) 37.5 mg PO (Ranitidine HCl) (n=9, children)<br>c) 50 mg IV (n=20, pregnant women)<br>150 mg PO (n=80, pregnant women)<br>d) 2.4 mg/kg IV bolus (n=27, infants)	a) 360 - 650 ng/ml (1.14 -2.07 µM)<br>b) 53.9 to 492 ng/ml (0.17 - 1.56 µM), mean 117 ng/ml (0.37 µM)<br>c) Cord blood:<br>50 mg IV: At delivery approx. 127 ng/ml<br>12 h after delivery 10-38 ng/ml<br>150 mg PO: approx. max. 200 ng/ml<br>d) 1520 ng/ml	a) [74])<br>b) [75]<br>c) [76]<br>d) [77]
Retinol	RDA: 800 µg	a) Baseline<br>b) Cord blood measurements (n=145)<br>c) N/A<br>d) Estimated intake: 0.5 - 1.5 mg/day (n > 1000 men)	a) 0.2 - 0.3 µg/ml (0.67 - 1.0 µM)<br>b) 0.7 - 1.3 µM<br>c) 0.3 - 0.7 µg/ml (1.0 - 2.3 µM)<br>d) approx. 1.7 - 3.0 µM	a) [78]<br>b) [79]<br>c) [80]<br>d) [81]

Non-teratogenic compound	Common therapeutic doses and dosing schemes in humans	Clinical study design, dosing scheme and other resources of information	Plasma peak or blood concentrations	Reference
Sucralose	RDA: 5 mg/kg/day<br><br>Max. dose: 15 mg/kg [82]	a) 1 mg/kg PO (adult men)<br>b) 5-10 mg/kg/day (adults)<br>c) 250 mg PO (adult men)	a) 140 - 460 ng/ml (350 - 1157 nM)<br>b) 5 mg/kg: 100 - 190 ng/ml;<br>10 mg/kg: max. 900 ng/ml (2.26 µM)<br>c) 1560 ng/ml (3.9 µM)	a) [83]<br>b) [84]<br>c) [85]

Table S 3: Teratogenic compounds and their human relevant plasma or blood concentrations at therapeutic doses. *Common therapeutic doses and dosing schemes were collected from www.drugs.com if not stated otherwise. Routes of application were as follows: PO= Peroral, IV=Intravenously, IM=Intramuscular or subcutaneously. RDA = Recommended daily allowance. RDA = Recommended daily allowance. EPA = Environmental protection agency*

Teratogenic compound	Common therapeutic doses and dosing schemes in humans	Clinical study design, dosing scheme and other resources of information	Plasma (peak) or blood concentrations	Reference
9-cis-Retinoic acid (Alitretinoin)	Topical application of 0.1 % alitretinoin containing gel up to four times daily [86]	Single oral dose of<br>a) 40 mg (men)<br>b) 40 mg daily + contraceptives (women)	a) 146 ng/ml - 272 ng/ml (0.49 - 0.91 µM)<br>b) 143 ng/ml	a-b) [87]
Acitretin	25 to 50 mg PO once a day	Single dose of<br>a) 50 mg<br>b) 50 mg (men)<br>c) 40 mg	a) 500 ng/ml<br>b) 400 ng/ml<br>c) 70 - 420 ng/ml	a) [88]<br>b) [89]<br>c) [90]
Actinomycin D	45 µg/kg or 1250 µg/m2 IV over 10 to 15 minutes once every 3 weeks	a) 0.2 -2 mg IV once (children)<br>b) 0.7-1.5 mg/m² IV bolus (n=31, children and adolescents)	a) 5-186 ng/ml (4 - 148 nM)<br>b) 3-100 ng/ml (2.4 - 80 nM)	a) [91]<br>b) [92]
Atorvastatin	10-80 mg PO daily	a) 80 mg PO once daily over 14 days (n=8, healthy adults)<br>b) 120 mg PO once (n=1) (Upper dose-limit)<br>c) In rats: 225 mg/kg daily	a) 252 ng/ml (0.45 µM)<br>b) 300 ng/ml (0.54 µM)<br>c) 5.8 µg/ml (10.44 µM)	a) [93]<br>b) [94]<br>c) [95]
Carbamazepine	200 - 1600 mg/day	a) 400 - 1000 mg once PO (pregnant women)<br>b) Up to 8 mg/kg (pregnant women)	a) Fetal cord blood: Up to 0.5 - 4.4 µg/ml<br>b) Fetal cord blood: Up to 4.5 µg/ml	a) [96]<br>b) [97]
Doxorubicin	Up to 75 mg/m² IV over 10 min each 21 days, 4 cycles in total; Lifetime cumulative dosis max. 550 mg/m²	a) 60 mg/m² IV over 40 min (female adults, adjuvant therapy with cyclophosphamide)<br>b) 4-day infusion 75 mg/m²<br>low-dose 25 mg/m² bolus IV<br>high-dose 75 mg/m² bolus IV<br>c) 75 mg/m² IV oder 15 min	a) 0.63 µg/ml<br>b) 4-day IV: 0.1 µg/ml<br>Low-dose: 2.3 µg/ml<br>High-dose: 3.7 µg/ml<br>c) 0.1 µM (0.05 µg/ml)	a) [98]<br>b) [99]<br>c) [100]
Entinostat (MS-275)	n/a	a) In vitro benchmark concentration<br>b) Max.8 mg/week	a) 208 nM<br>b) 5 ng/ml mean (0.12 µM), max.150 ng/ml (0,36 µM)	a) [101]<br>b) [13]
Favipiravir	1600 mg PO twice on first day, 600 mg PO twice following days [15]	a) 1st study (JP101): 30 - 1600 mg PO once (n=36, men)<br>2nd study (JP103): 400-600 mg PO, multiple dosing (n=18, men)<br>3rd study (JP111): 1200 single dose followed by 400-600 mg, multiple dosing (n=12, men)<br>b) 1800 mg twice on first day, followed by 800 mg twice/day (adults)<br>c) 600 mg PO twice/day (adults)	a) 1st study: 1.39 - 78 .6 µg/ml<br>2nd study: 17.2 - 43.8 µg/ml<br>3rd study: 40.5 -61.5 µg/ml<br>b) 35-50 µg/ml<br>c) Steady-state: 61.5 µg/ml	a) [15]<br>b) [102]<br>c) [103]

Teratogenic compound	Common therapeutic doses and dosing schemes in humans	Clinical study design, dosing scheme and other resources of information	Plasma (peak) or blood concentrations	Reference
**Isotretinoin**	**0.25 to 0.5 mg/kg PO twice a day**<br>**Maximum dose: Up to 2 mg/kg/day**	**a) 80 mg oral dose**<br>**b) 100 mg oral dose in man**	**a) 300 ng/ml (1 µM)**<br>**b) 74 to 511 ng/ml (1.7 µM)**	**a) [88]**<br>**b) [104]**
**Leflunomide**	**20 mg PO daily**	**5-100 mg PO, different dosing regimens (adults)**	**Steady-State: 4 -152 µg/ml**	**[105]**
**Lithium (chloride)**	**900-1800 mg PO daily**	**a) Recommended therapeutic plasma levels based on multiple studies**<br>**b) 600 mg**<br>**c) 1000 mg**	**a) 0.6-0,75 mM**<br>**b) 0.6 mM**<br>**Therapeutic 0.4 -1.1 mM,**<br>**Mid toxic 1.5 -2 mM,**<br>**Toxic >2 mM**<br>**c) 0.4 – 0.8 mM**	**a) [106]**<br>**b) [107]**<br>**c) [108]**
**Methotrexate**	**Acute lymphoblastic leukemia:**<br>**-Induction: 3.3 mg/m2/day orally for 4 to 6 weeks**<br>**-Maintenance dose during remission: 30 mg/m2 orally or IM twice a week**<br>**-Alternate maintenance dose during remission: 2.5 mg/kg IV every 14 days**<br><br>**Psoriasis:**<br>**10 to 30 mg/week PO, IM, IV or subcutaneously**<br><br>**Rheumatoid arthritis:**<br>**7.5 -20 mg PO or subcutaneously once a week**<br><br>**Osteosarcoma:**<br>**12-15 g/m2 IV as a 4-hour infusion**	**2.1 - 36 mg/m2 PO (n= 297, chilrden)**	**0.1 - 4.3 µM, mean 1.1 µM**	**[109]**
**Methylmercury**	**RDA: 0.1 µg/kg/day**	**a) Baseline concentration in Korean Population**<br>**b) RDA intake and EPA reference dose corresponding concentration**<br>**c) In vitro benchmark concentration**	**a) 4.5 µg/l (17,9 nM)**<br>**b) 5.8 µg/l**<br>**c) 1.5 µM**	**a) [110]**<br>**b) [111]**<br>**c) [101]**
**Panobinostat**	**20 mg PO every other day (3 doses per week)**	**a) In vitro benchmark concentration**<br>**b) 20 mg once**	**a) 4 nM**<br>**b) 20 ng/ml mean (57 nM)**	**a) [101]**<br>**b) [112]**

Teratogenic compound	Common therapeutic doses and dosing schemes in humans	Clinical study design, dosing scheme and other resources of information	Plasma (peak) or blood concentrations	Reference
**Paroxetine**	**20-60 mg PO daily**	**a) 20-50 mg PO single dose (n=29)<br>b) 30 mg/day PO over 30 days (n=15)<br>c) 40 mg PO daily, chronic dosing (n=30)**	**a) 2.5-65.1 ng/ml (7- 198 nM)<br>b) 8.6-105 ng/ml (26- 319 nM)<br>c) 1.7-407.0 ng/ml (7.9- 1237 nM)**	**a-c) [113]**
**Phenytoin (5,5-Diphenyl-hydantoin)**	**300 mg PO daily<br><br>Status epilepticus: max. 50 mg/min IV in 4 h**	**a) 100-700 mg IV (pregnant women)<br>b) 1.7 - 6.0 mg/kg (n=13, pregnant women)**	**a) Fetal cord blood: 0.4-13 µg/ml<br>b) Maternal blood = cord blood at delivery:<br>2.2 - 20 µg/ml**	**a) [96]<br>b) [114]**
**Teriflunomide (A-771726)**	**7-14 mg PO daily**	**refer to leflunomide**	**refer to leflunomide**	**refer to leflunomide**
**Thalidomide**	**100-400 mg PO daily**	**200 mg PO (n =45, adult men)**	**1-2 mg/l (3,9 µM)**	**[115]**
**Trichostatin A**	**n/a**	**In vitro benchmark concentration**	**10 nM**	**[101]**
**Valproic acid (VPA)**	**Maximum dose: 60 mg/kg daily<br><br>Therapeutic plasma levels: 50 - 100 µg/ml (347 - 693 µM)**	**50-1350 mg / day (pregnant women)**	**Maternal blood: 15.6 - 85 µg/ml<br>Fetal blood: 15.8 - 90 µg/ml**	**[96]**
**Vinblastine**	**up to 18.5 mg/m2 IV weekly**	**a) 7.5 mg/m² IV once<br>b) 7-11 mg/m² IV once<br>c) 3 mg/m² (coinjection cis-Platin) IV once<br>d) Mice: 12-35 mg/kg IV once<br>e) Mice**	**a) 10 -20 ng/ml (12,3 - 24,6 nM)<br>b) 20-40 ng/ml (24,6 - 59,2 nM)<br>c) 4,8 ng/ml (5,9 nM)<br>d) 10 ng/ml (12,3 nM)<br>e) 10 ng/ml (effective teratogenic fetal blood concentration in mice)**	**a) [116]<br>b) [117]<br>c) [118]<br>d) [119]<br>e) [120]**
**Vismodegib**	**150 mg PO daily**	**a) 150 mg PO once (adults with hepatic disfunctions)<br>b) 150 mg PO once or multiple dosing (healthy females)**	**a) 17 - 27 µM<br>b) Single dose: 6 µM<br>Multiple doses (steady-state): 16.4 µM**	**a) [121]<br>b) [122]**
**Vorinostat**	**400 mg PO daily**	**a) 400 mg PO once daily over 2 to 480+ days<br>b) In vitro benchmark concentration**	**a) 0.4-3 µM<br>b) 140 nM**	**a) [123]<br>b) [101]**

Table S 4: Placental transfer of non-teratogenic compounds

Negative compound	Placental transfer	Reference
Ampicillin	Yes (1:1)	[124]
Ascorbic acid	Yes; ascorbic acid enriches in the fetus 2- to 4-fold compared to maternal plasma / blood concentrations	[6]
Buspirone	Unknown	No information online at 18.11.2020, no information in [6]
Chlorpheniramine	Unknown	No information online at 18.11.2020, no information in [6]
Dextromethorphan	Unknown	No information online at 18.11.2020, no information in [6]
Diphenhydramine	In humans: Unknown<br>In sheep: Yes	[125]
Doxylamine	In humans: Unknown<br>In rhesus monkey: Yes, occurs at a rate of 30-60 %	[126]
Famotidine	Yes<br>(Fetal to maternal ratio of compound in blood/plasma: 0.64 ± 0.13)	[58]
Folic acid	Yes; folic acid enriches in the fetus 2- to 4-fold compared to maternal plasma / blood concentrations	[127;128]
Levothyroxine	Yes, occurs at a rate of estimated 30 %	[65;129;130]
Liothyronine	Yes	[67]
Magnesium (chloride)	Yes, almost 1:1<br>(Fetal to maternal ratio of compound in blood/plasma 0.94 ± 0.15)	[68]
Methicillin	Yes (1:1)	[124;73]
Ranitidine	Yes (Fetal to maternal ratio of compound in blood/plasma<br>- after peroral application: 0.38<br>- after intravenous application: 0.9)	[76]
Retinol	Yes	[78;131;132]
Sucralose	Unknown	No information online at 18.11.2020, no information in [6]

Table S 5: Predicted probabilities for teratogenicity

Compounds	Predicted probability for teratogenicity[b]	
	1-fold C_{max}[a]	20-fold C_{max}[a]
**Non-teratogens**		
Ampicillin	0.04	0.00
Ascorbic acid	0.00	0.02
Buspirone	0.03	0.03
Chlorpheniramine	0.00	0.01
Dextromethorphan	0.00	0.01
Diphenhydramine	0.44	0.15
Doxylamine	0.08	0.00
Famotidine	0.05	0.02
Folic acid	0.00	0.00
Levothyroxine	0.00	0.00
Liothyronine	0.00	0.01
Magnesium chloride	0.00	0.02
Methicillin	0.02	0.01
Ranitidine	0.28	0.07
Retinol	0.23	0.38
Sucralose	0.77	1.00
**Teratogens**		
9-cis-retinoic acid	0.73	0.91
Acitretin	0.99	1.00
Actinomycin D	1.00	1.00
Atorvastatin	0.01	0.02
Carbamazepine	0.89	1.00[c]
Doxorubicin	1.00	1.00
Entinostat	1.00	1.00
Favipiravir	0.89	0.98
Isotretinoin	1.00	1.00
Leflunomide	1.00	---[c]
Lithium chloride	0.76	0.96
Methotrexate	0.98	0.85
Methylmercury	0.91	0.70
Panobinostat	1.00	1.00
Paroxetine	0.47	0.34
Phenytoin	0.37	---[c]
Teriflunomide	1.00	---[c]
Thalidomide	0.33	0.41
Trichostatin A	1.00	1.00
Valproic acid	1.00	1.00[e]
Vinblastine	1.00	1.00
Vismodegib	0.78	---[c]
Vorinostat	1.00	1.00

[a]Maximal plasma or blood concentrations which were usually observed in humans after the administration of therapeutic compound doses (Table S 2, Table S 3). Fetal enrichment was considered if relevant (Table S 4)

[b]Probability of a compound to be a teratogen in the top-1,000 classifier

[c]Carbamazepine and VPA were tested at 10-fold and 1.67-fold C_{max}, respectively, instead of 20-fold C_{max}; leflunomide, phenytoin, teriflunomide and vismodegib were tested at 1-fold C_{max} due to limited solubility.

Table S 6: Classification of the in vitro test results in the two procedure

	SPS-procedure		Top 1000-procedure	
	**1-fold C_{max}[a]**	**20-fold C_{max}[a]**	**1-fold C_{max}[a]**	**20-fold C_{max}[a]**
**Non-teratogens**				
Ampicillin	TN	TN	TN	TN
Ascorbic acid	TN	FP	TN	TN
Buspirone	TN	TN	TN	TN
Chlorpheniramine	TN	TN	TN	TN
Dextromethorphan	TN	TN	TN	TN
Diphenhydramine	TN	TN	FP	TN
Doxylamine	TN	TN	TN	TN
Famotidine	TN	TN	TN	TN
Folic acid	TN	TN	TN	TN
Levothyroxine	TN	TN	TN	TN
Liothyronine	TN	TN	TN	TN
Magnesium (chloride)	TN	FP	TN	TN
Methicillin	TN	TN	TN	TN
Ranitidine	TN	TN	TN	TN
Retinol	TN	TN	TN	FP
Sucralose	TN	TN	FP	FP
**Teratogens**				
9-cis-retinoic acid	TP	TP	TP	TP
Acitretin	TP	TP	TP	TP
Actinomycin D	TP	TP	TP	TP
Atorvastatin	FN	TP	FN	FN
Carbamazepine	TP	TP[b]	TP	TP[b]
Doxorubicin	TP	TP	TP	TP
Entinostat	TP	TP	TP	TP
Favipiravir	FN	TP	TP	TP
Isotretinoin	TP	TP	TP	TP
Leflunomide	TP	--[b]	TP	--[b]
Lithium (chloride)	TP	TP	TP	TP
Methotrexate	TP	TP	TP	TP
Methylmercury	TP	FN	TP	TP
Panobinostat	TP	TP	TP	TP
Paroxetine	TP	TP	TP	TP
Phenytoin	FN	--[b]	TP	--[b]
Teriflunomide	TP	--[b]	TP	--[b]
Thalidomide	TP	TP	TP	TP
Trichostatin A	TP	TP	TP	TP
Valproic acid	TP	TP[b]	TP	TP[b]
Vinblastine	TP	TP	TP	TP
Vismodegib	FN	--[b]	TP	--[b]
Vorinostat	TP	TP	TP	TP

TN = True Negative; FN = False Negative; FP = False Positive; TP = True Positive

[a]Maximal plasma or blood concentrations which were usually observed in humans after the administration of therapeutic compound doses (Table S 2, Table S 3). Fetal enrichment was considered if relevant (Table S 4).

[b]Carbamazepine and VPA were tested at 10-fold and 1.67-fold C_{max}, respectively, instead of 20-fold C_{max}; leflunomide, phenytoin, teriflunomide and vismodegib were tested at 1-fold C_{max} due to limited solubility.

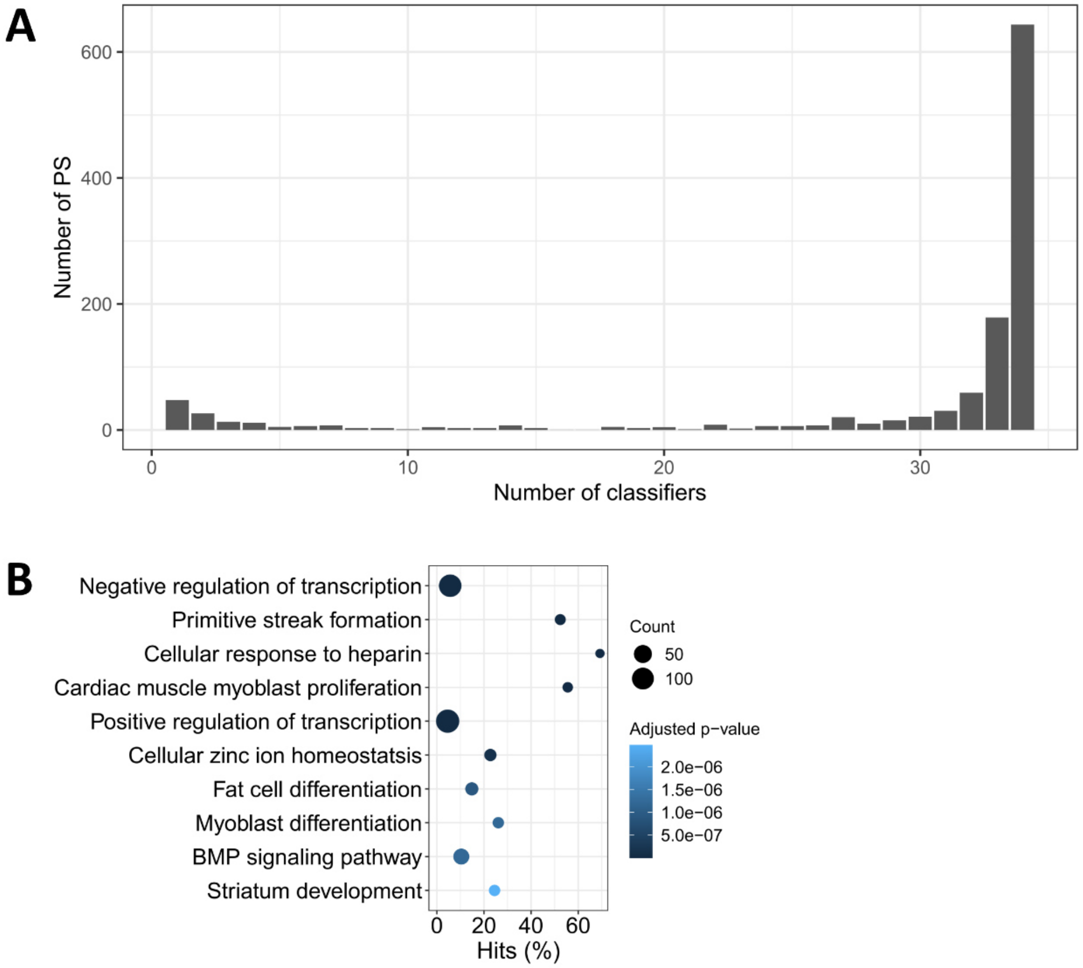

Fig. S1: Overlap-analysis (A) and GO-analysis (B) of the 1160 probe sets of the 34 classifiers of the top 1000 procedure. (A) Across all 34 classifiers that were constructed, a sum of 1160 different probe sets were used. Illustrated is the sum of probe sets (y-axis) that overlap in a given number of classifiers (x-axis). For example, >600 PS overlap in all 34 classifiers and an additional number of ~200 probe sets overlap in 33 of 34 classifiers, but not in all 34; ~50 probe sets do not overlap and were only found once in single classifiers. The minimal overlap between any two classifiers in a pairwise comparison is 865 PS (not shown). **(B)** Shown are the ten overrepresented GO-groups (adj. p-value <0.05**)** with the lowest adj. p-values. The names of the GO-groups were shortened. Full names and complete GO-group lists and in the SI “GO analysis”. “Count”: Number of genes from (A) linked to the GO group. “Hits”: Percentage of genes from (A) compared to all genes assigned to the GO group.

Fig. S2-S6:

Biological interpretation of genes differentially expressed after exposure of hiPSC to teratogens and non-teratogens. The following combinations were investigated:

- Fig. S2: Only upregulated probe sets at 1-fold C_{max}
- Fig. S3: Only downregulated probe sets at 1-fold C_{max}
- Fig. S4: All deregulated probe sets at 20-fold C_{max}
- Fig. S5: Only upregulated probe sets at 20-fold C_{max}
- Fig. S6: Only downregulated probe sets at 20-fold C_{max}
- **(A)** Numbers of significant probe sets (log2 fold change >1; adjusted p-value <0.05) induced by non-teratogens and teratogens at the given concentration **(B)** Top genes in the gene sets of the overlap, teratogens and non-teratogens from (A). The numbers in the bars indicate how many compounds deregulated the specific genes. All differential genes are given in the SI "Top genes". **(C)** Numbers of significantly (adj. p-value <0.05) overrepresented GO groups in the overlap, teratogen and non-teratogen gene sets. **(D)** The ten GO-groups with the lowest adj. p-values in the overlap and teratogen gene sets. No significant GO-groups were obtained for the non-teratogen gene set. The names of the GO-groups were shortened. Full names and complete GO-group lists and in the SI "GO analysis". "Count": Number of significant genes from (A) linked to the GO group. "Hits": Percentage of significant genes compared to all genes assigned to the GO group. **(E)** KEGG pathway enrichment analyses of the overlap and teratogen gene sets. The ten KEGG pathways with the lowest adj. p-values are given. No significant KEGG pathways were obtained for the non-teratogen gene set. Full names and complete KEGG-pathway lists are given in the SI "KEGG pathways". "Count": Number of significant genes from (A) linked to the KEGG pathway. "Gene Ratio": Percentage of significant genes associated to the pathway compared to the number of all significant genes associated to any pathway.

Fig. S2:
Upregulated probe sets at 1-fold C_{max}

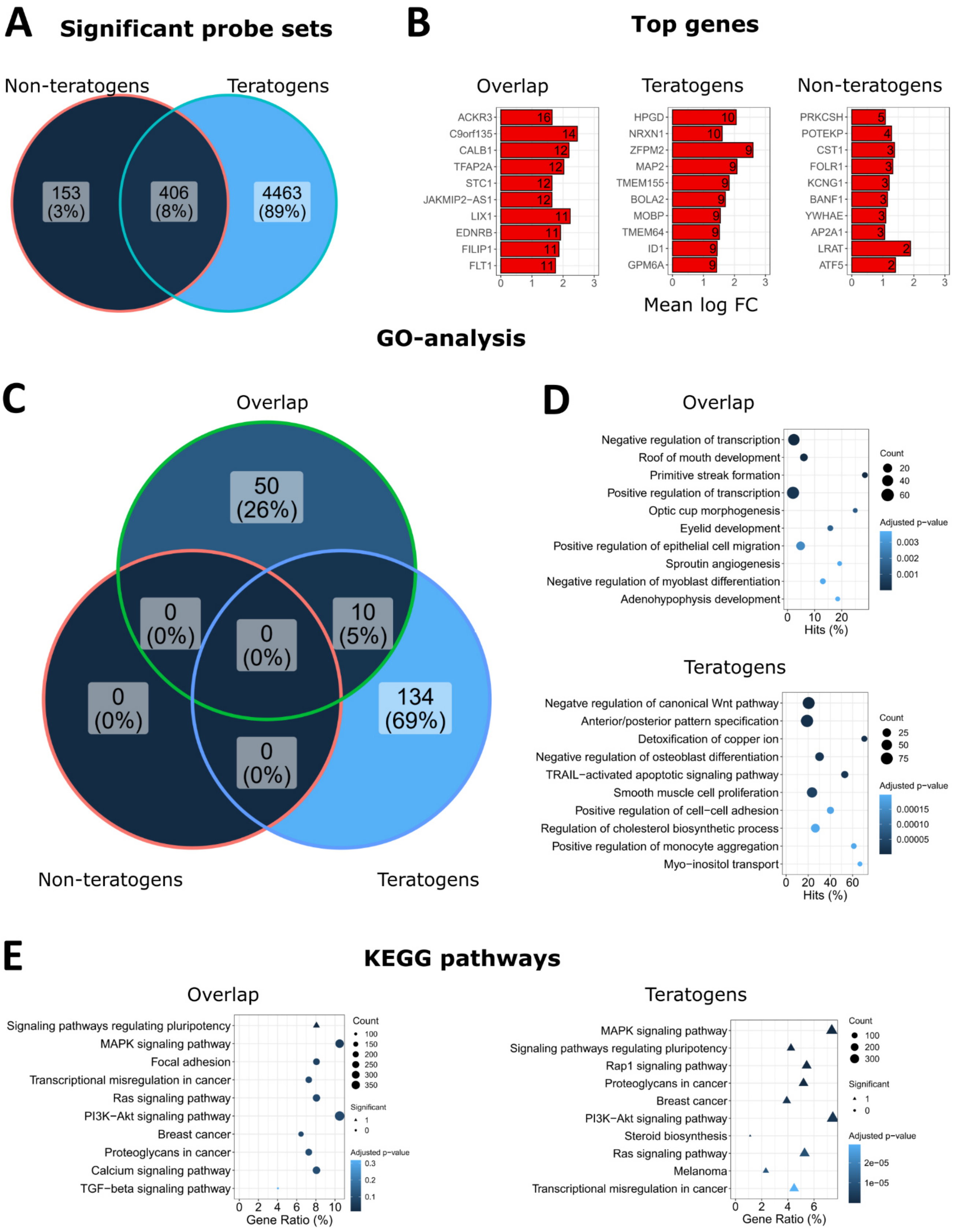

Fig. S3:
Downregulated probe sets at 1-fold C_{max}

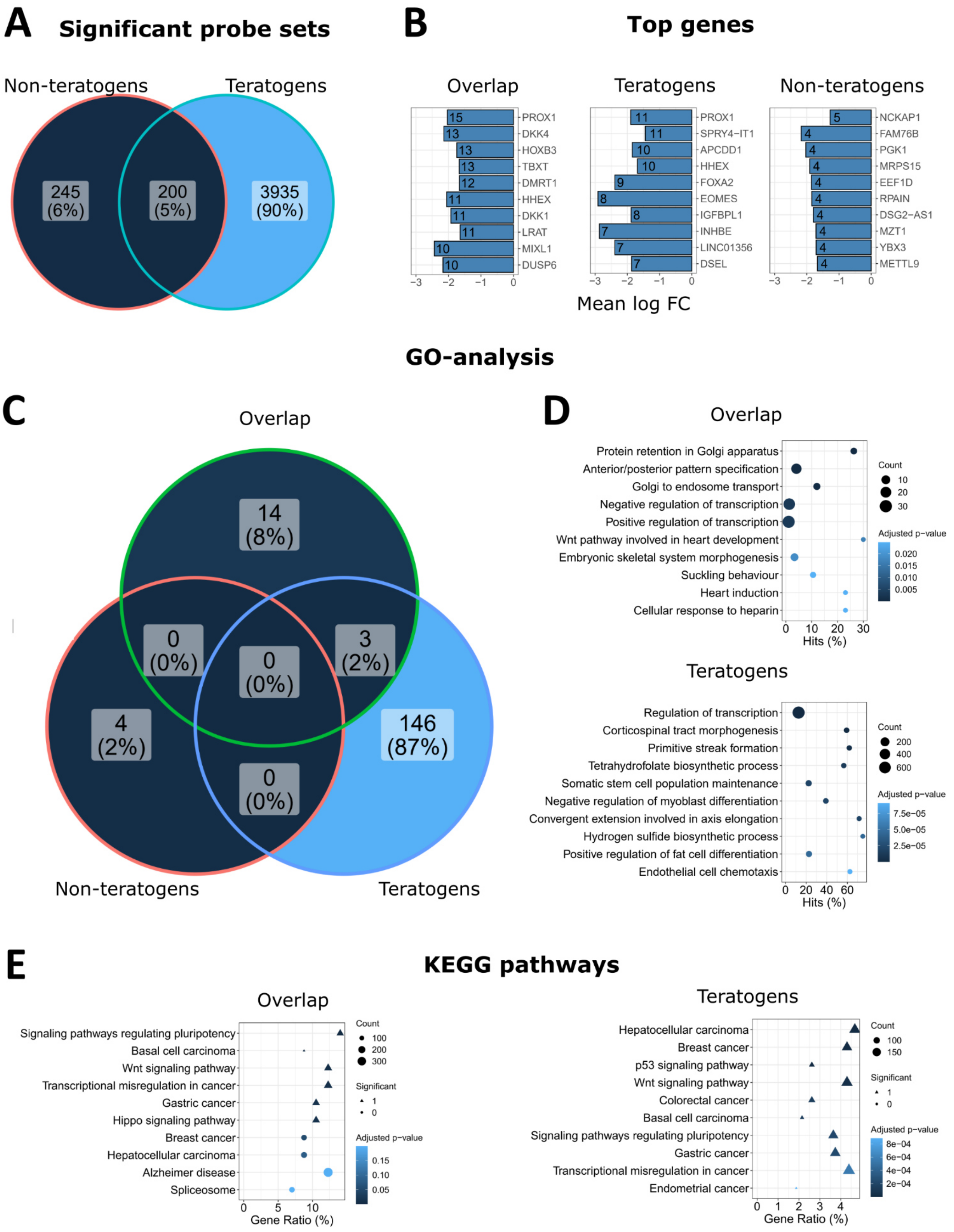

Fig. S4:
All deregulated probe sets at 20-fold C_{max}

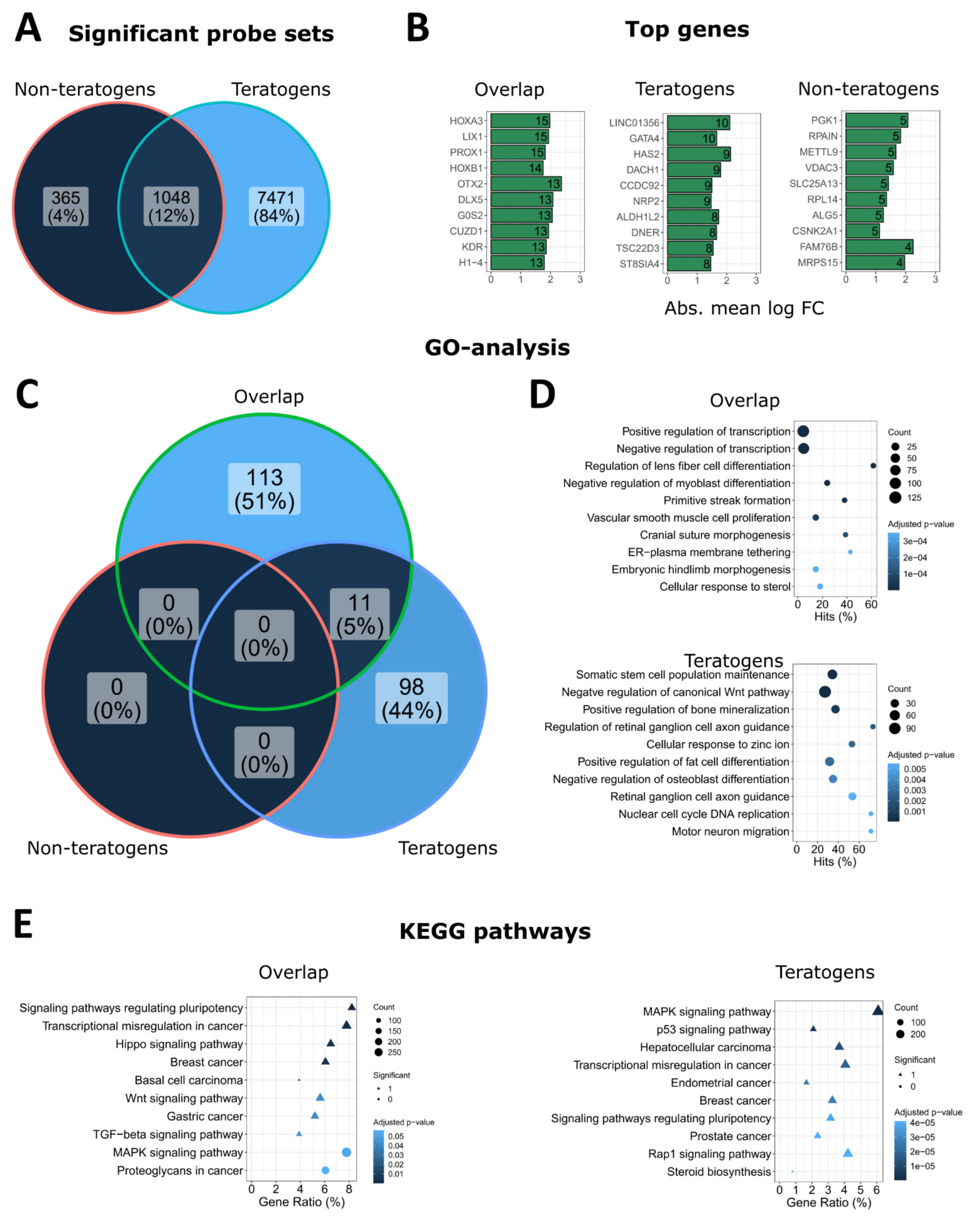

Fig. S5:

Upregulated probe sets at 20-fold C_{max}

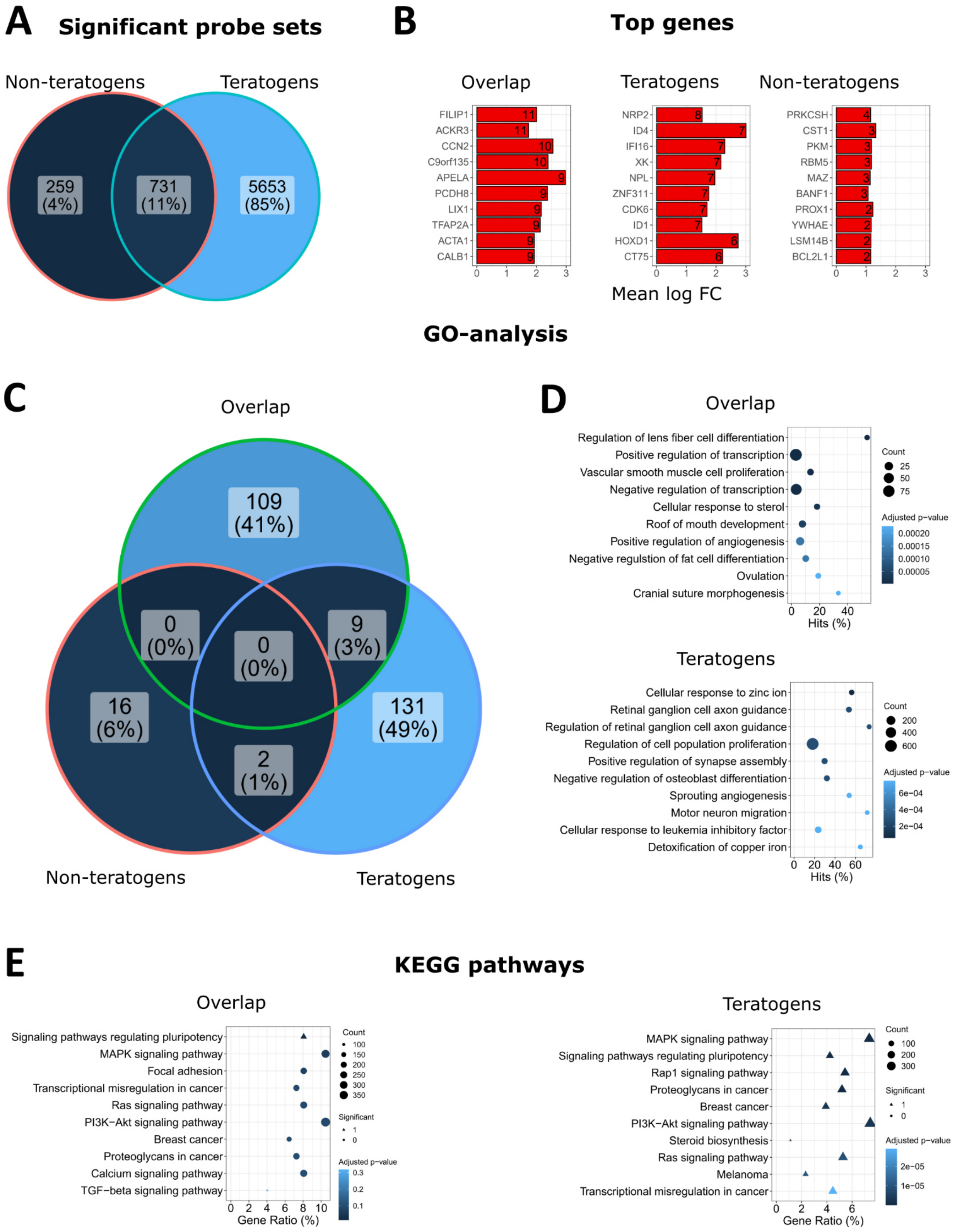

Fig. S6:

Downregulated probe sets at 20-fold C_{max}

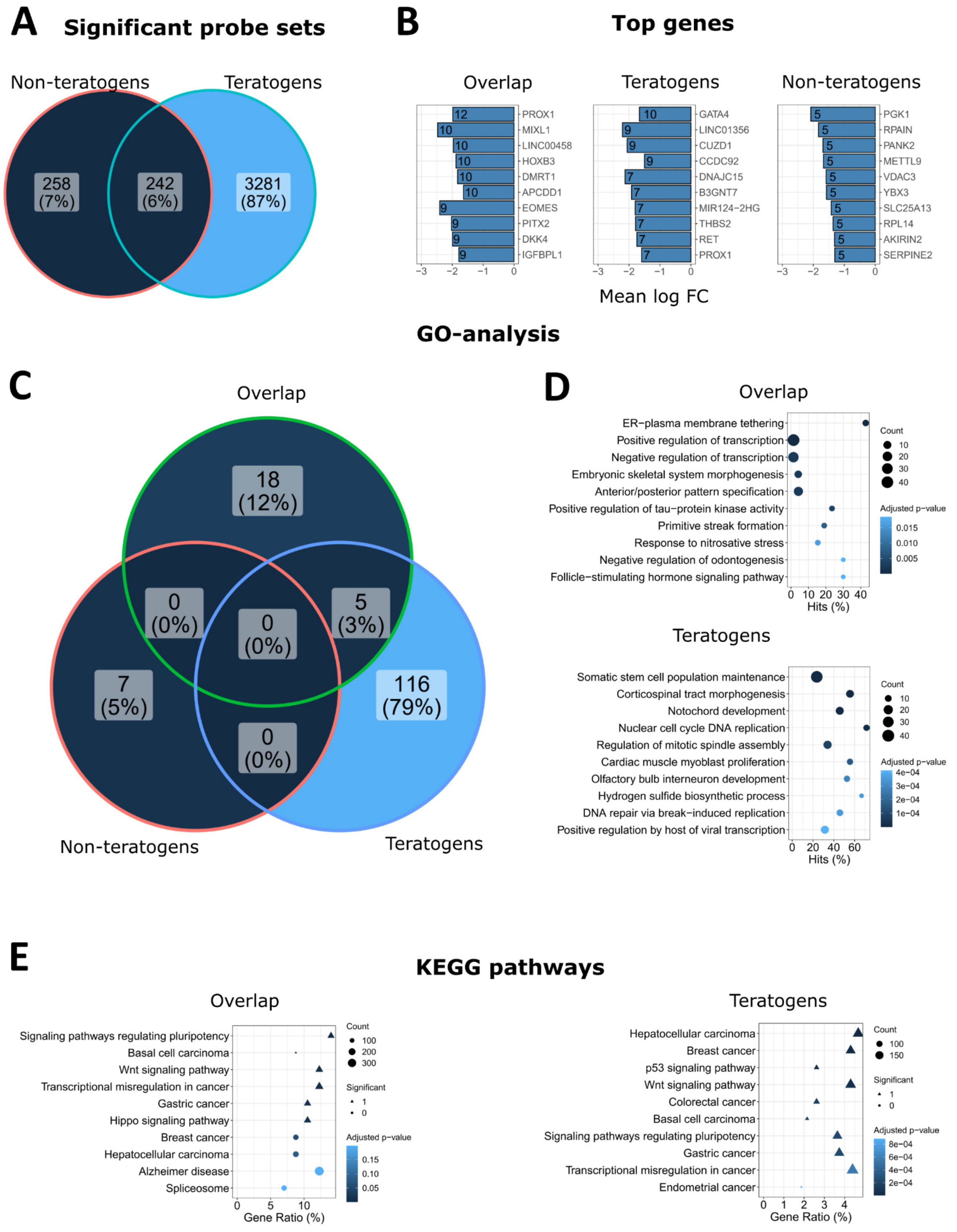

References

[1] Comptour, Aurélie; Rouzaire, Marion; Belville, Corinne; Bouvier, Damien; Gallot, Denis; Blanchon, Loïc; Sapin, Vincent (**2016**): Nuclear retinoid receptors and pregnancy: placental transfer, functions, and pharmacological aspects. In *Cellular and molecular life sciences : CMLS* 73 (20), pp. 3823–3837. DOI: 10.1007/s00018-016-2332-9.

[2] Allenby, G.; Bocquel, M. T.; Saunders, M.; Kazmer, S.; Speck, J.; Rosenberger, M. et al. (**1993**): Retinoic acid receptors and retinoid X receptors: interactions with endogenous retinoic acids. In *Proceedings of the National Academy of Sciences of the United States of America* 90 (1), pp. 30–34. DOI: 10.1073/pnas.90.1.30.

[3] Idres, Nadia; Marill, Julie; Flexor, Maria A.; Chabot, Guy G. (**2002**): Activation of retinoic acid receptor-dependent transcription by all-trans-retinoic acid metabolites and isomers. In *The Journal of biological chemistry* 277 (35), pp. 31491–31498. DOI: 10.1074/jbc.M205016200.

[4] Kochhar, D. M.; Jiang, H.; Penner, J. D.; Heyman, R. A. (**1995**): Placental transfer and developmental effects of 9-cis retinoic acid in mice. In *Teratology* 51 (4), pp. 257–265. DOI: 10.1002/tera.1420510411.

[5] Pilkington, Tania; Brogden, Rex N. (**1992**): Acitretin. In *Drugs* 43 (4), pp. 597–627. DOI: 10.2165/00003495-199243040-00010.

[6] Briggs, Gerald G.; Towers, Craig V.; Freeman, Roger K. (**2017**): Drugs in pregnancy and lactation. A reference guide to fetal and neonatal risk. Eleventh edition. Philadelphia, PA: Wolters Kluwer. Available online at http://search.ebscohost.com/login.aspx?direct=true&scope=site&db=nlebk&AN=1857599.

[7] Hollstein, Ulrich (**1974**): Actinomycin. Chemistry and mechanism of action. In *Chem. Rev.* 74 (6), pp. 625–652. DOI: 10.1021/cr60292a002.

[8] Tuchmann-Duplessis, H.; Hiss, D.; Mottot, G.; Rosner, I. (**1973**): Embryotoxic and teratogenic effect of actinomycin D in the syrian hamster. In *Toxicology* 1 (2), pp. 131–133. DOI: 10.1016/0300-483X(73)90025-5.

[9] Lecarpentier, Edouard; Morel, Olivier; Fournier, Thierry; Elefant, Elisabeth; Chavatte-Palmer, Pascale; Tsatsaris, Vassilis (**2012**): Statins and pregnancy: between supposed risks and theoretical benefits. In *Drugs* 72 (6), pp. 773–788. DOI: 10.2165/11632010-000000000-00000.

[10] Rogawski, Michael A.; Löscher, Wolfgang; Rho, Jong M. (**2016**): Mechanisms of Action of Antiseizure Drugs and the Ketogenic Diet. In *Cold Spring Harbor perspectives in medicine* 6 (5). DOI: 10.1101/cshperspect.a022780.

[11] Matalon, S.; Schechtman, S.; Goldzweig, G.; Ornoy, A. (**2002**): The teratogenic effect of carbamazepine: a meta-analysis of 1255 exposures. In *Reproductive Toxicology* 16 (1), pp. 9–17. DOI: 10.1016/S0890-6238(01)00199-X.

[12] Varela-López, Alfonso; Battino, Maurizio; Navarro-Hortal, María D.; Giampieri, Francesca; Forbes-Hernández, Tamara Y.; Romero-Márquez, José M. et al. (**2019**): An update on the mechanisms related to cell death and toxicity of doxorubicin and the protective role of nutrients. In *Food and chemical toxicology : an international journal published for the British Industrial Biological Research Association* 134, p. 110834. DOI: 10.1016/j.fct.2019.110834.

[13] Connolly, Roisin M.; Rudek, Michelle A.; Piekarz, Richard (**2017**): Entinostat: a promising treatment option for patients with advanced breast cancer. In *Future oncology (London, England)* 13 (13), pp. 1137–1148. DOI: 10.2217/fon-2016-0526.

[14] Furuta, Yousuke; Gowen, Brian B.; Takahashi, Kazumi; Shiraki, Kimiyasu; Smee, Donald F.; Barnard, Dale L. (**2013**): Favipiravir (T-705), a novel viral RNA polymerase inhibitor. In *Antiviral research* 100 (2), pp. 446–454. DOI: 10.1016/j.antiviral.2013.09.015.

[15] Pharmaceutical and Food Safety Bureau (**2014**): Report on the Deliberation Results for Avigan Tablet 200 mg. Available online at https://www.pmda.go.jp/files/000210319.pdf, checked on 11/27/2020.

[16] Nau, H. (**2001**): Teratogenicity of isotretinoin revisited: species variation and the role of all-trans-retinoic acid. In *Journal of the American Academy of Dermatology* 45 (5), S183-7. DOI: 10.1067/mjd.2001.113720.

[17] Browne, Hannah; Mason, Gerald; Tang, Thomas (**2014**): Retinoids and pregnancy: an update. In *Obstet Gynecol* 16 (1), pp. 7–11. DOI: 10.1111/tog.12075.

[18] Fox, R. I.; Herrmann, M. L.; Frangou, C. G.; Wahl, G. M.; Morris, R. E.; Strand, V.; Kirschbaum, B. J. (**1999**): Mechanism of action for leflunomide in rheumatoid arthritis. In *Clinical immunology (Orlando, Fla.)* 93 (3), pp. 198–208. DOI: 10.1006/clim.1999.4777.

[19] Malhi, Gin S.; Tanious, Michelle; Das, Pritha; Coulston, Carissa M.; Berk, Michael (**2013**): Potential mechanisms of action of lithium in bipolar disorder. Current understanding. In *CNS drugs* 27 (2), pp. 135–153. DOI: 10.1007/s40263-013-0039-0.

[20] Cronstein, Bruce N.; Aune, Thomas M. (**2020**): Methotrexate and its mechanisms of action in inflammatory arthritis. In *Nature reviews. Rheumatology* 16 (3), pp. 145–154. DOI: 10.1038/s41584-020-0373-9.

[21] Verberne, Eline A.; Haan, Emma de; van Tintelen, J. Peter; Lindhout, Dick; van Haelst, Mieke M. (**2019**): Fetal methotrexate syndrome: A systematic review of case reports. In *Reproductive toxicology (Elmsford, N.Y.)* 87, pp. 125–139. DOI: 10.1016/j.reprotox.2019.05.066.

[22] Hyoun, Sara C.; Običan, Sarah G.; Scialli, Anthony R. (**2012**): Teratogen update: methotrexate. In *Birth defects research. Part A, Clinical and molecular teratology* 94 (4), pp. 187–207. DOI: 10.1002/bdra.23003.

[23] Fretham, Stephanie Jb; Caito, Samuel; Martinez-Finley, Ebany J.; Aschner, Michael (**2012**): Mechanisms and Modifiers of Methylmercury-Induced Neurotoxicity. In *Toxicology Research* 1 (1), pp. 32–38. DOI: 10.1039/C2TX20010D.

[24] Farina, Marcelo; Rocha, João B. T.; Aschner, Michael (**2011**): Mechanisms of methylmercury-induced neurotoxicity: evidence from experimental studies. In *Life sciences* 89 (15-16), pp. 555–563. DOI: 10.1016/j.lfs.2011.05.019.

[25] Unoki, Takamitsu; Akiyama, Masahiro; Kumagai, Yoshito; Gonçalves, Filipe Marques; Farina, Marcelo; Da Rocha, João Batista Teixeira; Aschner, Michael (**2018**): Molecular Pathways Associated With Methylmercury-Induced Nrf2 Modulation. In *Frontiers in genetics* 9, p. 373. DOI: 10.3389/fgene.2018.00373.

[26] EPA (**2012**): Mercury Study Report to Congress. Volume V: Health Effects of Mercury and Mercury Compounds. Environmental Protection Agency. Available online at https://www.epa.gov/mercury/mercury-study-report-congress, checked on 1/22/2021.

[27] Harada, M. (**1995**): Minamata disease: methylmercury poisoning in Japan caused by environmental pollution. In *Critical reviews in toxicology* 25 (1), pp. 1–24. DOI: 10.3109/10408449509089885.

[28] Grandjean, Philippe; Weihe, Pal; White, Roberta F.; Debes, Frodi; Araki, Shunichi; Yokoyama, Kazuhito et al. (**1997**): Cognitive Deficit in 7-Year-Old Children with Prenatal Exposure to Methylmercury. In *Neurotoxicology and Teratology* 19 (6), pp. 417–428. DOI: 10.1016/S0892-0362(97)00097-4.

[29] Karagas, Margaret R.; Choi, Anna L.; Oken, Emily; Horvat, Milena; Schoeny, Rita; Kamai, Elizabeth et al. (**2012**): Evidence on the human health effects of low-level methylmercury exposure. In *Environmental health perspectives* 120 (6), pp. 799–806. DOI: 10.1289/ehp.1104494.

[30] Novartis Pharmaceuticals (**2016**): Farydak Prescribing Information. Available online at https://www.accessdata.fda.gov/drugsatfda_docs/label/2015/205353s000lbl.pdf, checked on 1/20/2021.

[31] Davis, Bruce A.; Nagarajan, Anu; Forrest, Lucy R.; Singh, Satinder K. (**2016**): Mechanism of Paroxetine (Paxil) Inhibition of the Serotonin Transporter. In *Sci Rep* 6, p. 23789. DOI: 10.1038/srep23789.

[32] Vargesson, Neil (**2015**): Thalidomide-induced teratogenesis: history and mechanisms. In *Birth defects research. Part C, Embryo today : reviews* 105 (2), pp. 140–156. DOI: 10.1002/bdrc.21096.

[33] Xu, W. S.; Parmigiani, R. B.; Marks, P. A. (**2007**): Histone deacetylase inhibitors: molecular mechanisms of action. In *Oncogene* 26 (37), pp. 5541–5552. DOI: 10.1038/sj.onc.1210620.

[34] Ornoy, Asher (**2009**): Valproic acid in pregnancy: how much are we endangering the embryo and fetus? In *Reproductive toxicology (Elmsford, N.Y.)* 28 (1), pp. 1–10. DOI: 10.1016/j.reprotox.2009.02.014.

[35] Dhamodharan, R.; Jordan, M. A.; Thrower, D.; Wilson, L.; Wadsworth, P. (**1995**): Vinblastine suppresses dynamics of individual microtubules in living interphase cells. In *Molecular biology of the cell* 6 (9), pp. 1215–1229. DOI: 10.1091/mbc.6.9.1215.

[36] Ferm, V. H. (**1963**): Congenital malformations in hamster embryos after treatment with vinblastine and vincristine. In *Science (New York, N.Y.)* 141 (3579), p. 426. DOI: 10.1126/science.141.3579.426.

[37] Genentech (**2012**): Erivedge Product Information. Available online at https://www.accessdata.fda.gov/drugsatfda_docs/label/2012/203388lbl.pdf, checked on 1/21/2021.

[38] Morinello, Eric; Pignatello, Michael; Villabruna, Loris; Goelzer, Petra; Bürgin, Heinrich (**2014**): Embryofetal development study of vismodegib, a hedgehog pathway inhibitor, in rats. In *Birth defects research. Part B, Developmental and reproductive toxicology* 101 (2), pp. 135–143. DOI: 10.1002/bdrb.21093.

[39] Merck Sharp & Dohme (**2018**): Zolinza Prescribing Information. Available online at https://www.merck.com/product/usa/pi_circulars/z/zolinza/zolinza_pi.pdf, checked on 1/20/2021.

[40] Giachetto, Gustavo; Pirez, María Catalina; Nanni, Luciana; Martínez, Adriana; Montano, Alicia; Algorta, Gabriela et al. (**2004**): Ampicillin and penicillin concentration in serum and pleural fluid of hospitalized children with community-acquired pneumonia. In *The Pediatric infectious disease journal* 23 (7), pp. 625–629. DOI: 10.1097/01.inf.0000128783.11218.c9.

[41] Emmerson, A. M.; Cox, D. A.; Lees, L. J. (**1983**): Pharmacokinetics of sulbactam and ampicillin following oral administration of sultamicillin with probenecid. In *European journal of clinical microbiology* 2 (4), pp. 340–344. DOI: 10.1007/BF02019464.

[42] Padayatty, Sebastian J.; Sun, He; Wang, Yaohui; Riordan, Hugh D.; Hewitt, Stephen M.; Katz, Arie et al. (**2004**): Vitamin C pharmacokinetics: implications for oral and intravenous use. In *Annals of internal medicine* 140 (7), pp. 533–537. DOI: 10.7326/0003-4819-140-7-200404060-00010.

[43] Levine, M.; Conry-Cantilena, C.; Wang, Y.; Welch, R. W.; Washko, P. W.; Dhariwal, K. R. et al. (**1996**): Vitamin C pharmacokinetics in healthy volunteers: evidence for a recommended dietary allowance. In *Proceedings of the National Academy of Sciences of the United States of America* 93 (8), pp. 3704–3709. DOI: 10.1073/pnas.93.8.3704.

[44] Mahmood, I.; Sahajwalla, C. (**1999**): Clinical pharmacokinetics and pharmacodynamics of buspirone, an anxiolytic drug. In *Clinical Pharmacokinetics* 36 (4), pp. 277–287. DOI: 10.2165/00003088-199936040-00003.

[45] Dalhoff, K.; Poulsen, H. E.; Garred, P.; Placchi, M.; Gammans, R. E.; Mayol, R. F.; Pfeffer, M. (**1987**): Buspirone pharmacokinetics in patients with cirrhosis. In *British Journal of Clinical Pharmacology* 24 (4), pp. 547–550. DOI: 10.1111/j.1365-2125.1987.tb03210.x.

[46] Lamberg, T. S.; Kivistö, K. T.; Neuvonen, P. J. (**1998**): Concentrations and effects of buspirone are considerably reduced by rifampicin. In *British Journal of Clinical Pharmacology* 45 (4), pp. 381–385. DOI: 10.1046/j.1365-2125.1998.t01-1-00698.x.

[47] Huang, S. M.; Athanikar, N. K.; Sridhar, K.; Huang, Y. C.; Chiou, W. L. (**1982**): Pharmacokinetics of chlorpheniramine after intravenous and oral administration in normal adults. In *European journal of clinical pharmacology* 22 (4), pp. 359–365. DOI: 10.1007/BF00548406.

[48] Tagawa, Masaaki; Kano, Michiko; Okamura, Nobuyuki; Higuchi, Makoto; Matsuda, Michiaki; Mizuki, Yasuyuki et al. (**2002**): Differential cognitive effects of ebastine and (+)-chlorpheniramine in healthy subjects: correlation between cognitive impairment and plasma drug concentration. In *British Journal of Clinical Pharmacology* 53 (3), pp. 296–304. DOI: 10.1046/j.0306-5251.2001.01183.x.

[49] Schadel, M.; Wu, D.; Otton, S. V.; Kalow, W.; Sellers, E. M. (**1995**): Pharmacokinetics of dextromethorphan and metabolites in humans: influence of the CYP2D6 phenotype and quinidine inhibition. In *Journal of clinical psychopharmacology* 15 (4), pp. 263–269. DOI: 10.1097/00004714-199508000-00005.

[50] Pope, Laura E.; Khalil, M. H.; Berg, James E.; Stiles, Mark; Yakatan, Gerald J.; Sellers, Edward M. (**2004**): Pharmacokinetics of dextromethorphan after single or multiple dosing in combination with quinidine in extensive and poor metabolizers. In *Journal of clinical pharmacology* 44 (10), pp. 1132–1142. DOI: 10.1177/0091270004269521.

[51] Luna, B. G.; Scavone, J. M.; Greenblatt, D. J. (**1989**): Doxylamine and diphenhydramine pharmacokinetics in women on low-dose estrogen oral contraceptives. In *Journal of clinical pharmacology* 29 (3), pp. 257–260. DOI: 10.1002/j.1552-4604.1989.tb03323.x.

[52] Carruthers, S. G.; Shoeman, D. W.; Hignite, C. E.; Azarnoff, D. L. (**1978**): Correlation between plasma diphenhydramine level and sedative and antihistamine effects. In *Clinical Pharmacology and Therapeutics* 23 (4), pp. 375–382. DOI: 10.1002/cpt1978234375.

[53] Gelotte, Cathy K.; Zimmerman, Brenda A.; Thompson, Gary A. (**2018**): Single-Dose Pharmacokinetic Study of Diphenhydramine HCl in Children and Adolescents. In *Clinical pharmacology in drug development* 7 (4), pp. 400–407. DOI: 10.1002/cpdd.391.

[54] Köppel, C.; Ibe, K.; Tenczer, J. (**1987**): Clinical symptomatology of diphenhydramine overdose: an evaluation of 136 cases in 1982 to 1985. In *Journal of toxicology. Clinical toxicology* 25 (1-2), pp. 53–70. DOI: 10.3109/15563658708992613.

[55] Videla, Sebastián; Cebrecos, Jesús; Lahjou, Mounia; Wagner, France; Guibord, Pascal; Xu, Zhengguo et al. (**2013**): Pharmacokinetic dose proportionality between two strengths (12.5 mg and 25 mg) of doxylamine hydrogen succinate film-coated tablets in fasting state: a single-dose, randomized, two-period crossover study in healthy volunteers. In *Drugs in R&D* 13 (2), pp. 129–135. DOI: 10.1007/s40268-013-0015-7.

[56] Morgan, M. Y.; Stambuk, D.; Cottrell, J.; Mann, S. G. (**1990**): Pharmacokinetics of famotidine in normal subjects and in patients with chronic liver disease. In *Alimentary pharmacology & therapeutics* 4 (1), pp. 83–96. DOI: 10.1111/j.1365-2036.1990.tb00452.x.

[57] Chremos, A. N. (**1987**): Clinical pharmacology of famotidine: a summary. In *Journal of clinical gastroenterology* 9 Suppl 2, pp. 7–12. DOI: 10.1097/00004836-198707002-00003.

[58] Doi, H.; Maruta, H.; Kudoh, I.; Takahashi, Y.; Takano, O.; Ogawa, K. et al. (**1991**): Placental transfer and effects of famotidine on neonates. In *J Anesth* 5 (3), pp. 276–280. DOI: 10.1007/s0054010050276.

[59] Nguyen, Patricia; Boskovic, Rada; Yazdani, Parvaneh; Kapur, Bhushan; Vandenberghe, Hilde; Koren, Gideon (**2008**): Comparing folic acid pharmacokinetics among women of childbearing age: single dose ingestion of 1.1 versus 5 MG folic acid. In *The Canadian journal of clinical pharmacology = Journal canadien de pharmacologie clinique* 15 (2), e314-22.

[60] Obeid, Rima; Kasoha, Mariz; Kirsch, Susanne H.; Munz, Winfried; Herrmann, Wolfgang (**2010**): Concentrations of unmetabolized folic acid and primary folate forms in pregnant women at delivery and in umbilical cord blood. In *The American journal of clinical nutrition* 92 (6), pp. 1416–1422. DOI: 10.3945/ajcn.2010.29361.

[61] Tanguay, Mario; Girard, Johanne; Scarsi, Claudia; Mautone, Giuseppe; Larouche, Richard (**2019**): Pharmacokinetics and Comparative Bioavailability of a Levothyroxine Sodium Oral Solution and Soft Capsule. In *Clinical pharmacology in drug development* 8 (4), pp. 521–528. DOI: 10.1002/cpdd.608.

[62] Kashiwagura, Yasuharu; Uchida, Shinya; Tanaka, Shimako; Watanabe, Hiroshi; Masuzawa, Masahiro; Sasaki, Tadanori; Namiki, Noriyuki (**2014**): Clinical efficacy and pharmacokinetics of levothyroxine suppository in patients with hypothyroidism. In *Biological & pharmaceutical bulletin* 37 (4), pp. 666–670. DOI: 10.1248/bpb.b13-00998.

[63] Yue, C. S.; Scarsi, C.; Ducharme, M. P. (**2012**): Pharmacokinetics and potential advantages of a new oral solution of levothyroxine vs. other available dosage forms. In *Arzneimittel-Forschung* 62 (12), pp. 631–636. DOI: 10.1055/s-0032-1329951.

[64] Celi, Francesco S.; Zemskova, Marina; Linderman, Joyce D.; Babar, Nabeel I.; Skarulis, Monica C.; Csako, Gyorgy et al. (**2010**): The pharmacodynamic equivalence of levothyroxine and liothyronine: a randomized, double blind, cross-over study in thyroidectomized patients. In *Clinical endocrinology* 72 (5), pp. 709–715. DOI: 10.1111/j.1365-2265.2009.03700.x.

[65] Vulsma, T.; Gons, M. H.; Vijlder, J. J. de (**1989**): Maternal-fetal transfer of thyroxine in congenital hypothyroidism due to a total organification defect or thyroid agenesis. In *The New England journal of medicine* 321 (1), pp. 13–16. DOI: 10.1056/NEJM198907063210103.

[66] Jonklaas, Jacqueline; Burman, Kenneth D.; Wang, Hong; Latham, Keith R. (**2015**): Single-dose T3 administration: kinetics and effects on biochemical and physiological parameters. In *Therapeutic drug monitoring* 37 (1), pp. 110–118. DOI: 10.1097/FTD.0000000000000113.

[67] Dussault, J.; Row, V. V.; Lickrish, G.; Volpé, R. (**1969**): Studies of serum triiodothyronine concentration in maternal and cord blood: transfer of triiodothyronine across the human placenta. In *The Journal of clinical endocrinology and metabolism* 29 (4), pp. 595–603. DOI: 10.1210/jcem-29-4-595.

[68] Brookfield, Kathleen F.; Su, Felice; Elkomy, Mohammed H.; Drover, David R.; Lyell, Deirdre J.; Carvalho, Brendan (**2016**): Pharmacokinetics and placental transfer of magnesium sulfate in pregnant women. In *American Journal of Obstetrics and Gynecology* 214 (6), 737.e1-9. DOI: 10.1016/j.ajog.2015.12.060.

[69] Dolberg, Mette Konow Bøgebjerg; Nielsen, Lars Peter; Dahl, Ronald (**2017**): Pharmacokinetic Profile of Oral Magnesium Hydroxide. In *Basic & clinical pharmacology & toxicology* 120 (3), pp. 264–269. DOI: 10.1111/bcpt.12642.

[70] Wilimzig, C.; Latz, R.; Vierling, W.; Mutschler, E.; Trnovec, T.; Nyulassy, S. (**1996**): Increase in magnesium plasma level after orally administered trimagnesium dicitrate. In *European journal of clinical pharmacology* 49 (4), pp. 317–323. DOI: 10.1007/BF00226334.

[71] Simon, H. J.; Rantz, L. A. (**1962**): The newer penicillins. II. Clinical experiences with methicillin and oxacillin. In *Annals of internal medicine* 57, pp. 344–362. DOI: 10.7326/0003-4819-57-3-344.

[72] Sørensen, T. Sandberg; Petersen, O.; Heerfordt, J. (**1982**): The Pharmacokinetics of Methicillin and Dicloxacillin in Wound Fluid Following Internal Fixation of Trochanteric Fractures. In *Acta Orthopaedica Scandinavica* 53 (4), pp. 535–539. DOI: 10.3109/17453678208992253.

[73] Depp, Richard; Kind, Allan C.; Kirby, William M.M.; Johnson, Wayne L. (**1970**): Transplacental passage of methicillin and dicloxacillin into the fetus and amniotic fluid. In *American Journal of Obstetrics and Gynecology* 107 (7), pp. 1054–1057. DOI: 10.1016/0002-9378(70)90628-9.

[74] Richards, D. A. (**1983**): Comparative pharmacodynamics and pharmacokinetics of cimetidine and ranitidine. In *Journal of clinical gastroenterology* 5 Suppl 1, pp. 81–90. DOI: 10.1097/00004836-198312001-00008.

[75] Blumer, Jeffrey L.; Rothstein, Fred C.; Kaplan, Barbara S.; Yamashita, Toyoko S.; Eshelman, Fred N.; Myers, Carolyn M.; Reed, Michael D. (**1985**): Pharmacokinetic determination of ranitidine pharmacodynamics in pediatric ulcer disease. In *The Journal of pediatrics* 107 (2), pp. 301–306. DOI: 10.1016/s0022-3476(85)80156-6.

[76] McAuley, D. M.; Moore, J.; McCaughey, W.; Donnelly, B. D.; Dundee, J. W. (**1983**): Ranitidine as an antacid before elective Caesarean section. In *Anaesthesia* 38 (2), pp. 108–114. DOI: 10.1111/j.1365-2044.1983.tb13927.x.

[77] Fontana, M.; Massironi, E.; Rossi, A.; Vaglia, P.; Gancia, G. P.; Tagliabue, P.; Principi, N. (**1993**): Ranitidine pharmacokinetics in newborn infants. In *Archives of disease in childhood* 68 (5 Spec No), pp. 602–603. DOI: 10.1136/adc.68.5_spec_no.602.

[78] Sklan, D.; Shalit, I.; Lasebnik, N.; Spirer, Z.; Weisman, Y. (**1985**): Retinol transport proteins and concentrations in human amniotic fluid, placenta, and fetal and maternal sera. In *The British journal of nutrition* 54 (3), pp. 577–583. DOI: 10.1079/bjn19850144.

[79] Manolescu, Daniel C.; El-Kares, Reyhan; Lakhal-Chaieb, Lajmi; Montpetit, Alexandre; Bhat, Pangala V.; Goodyer, Paul (**2010**): Newborn serum retinoic acid level is associated with variants of genes in the retinol metabolism pathway. In *Pediatric research* 67 (6), pp. 598–602. DOI: 10.1203/PDR.0b013e3181dcf18a.

[80] Repetto, M. R.; Repetto, M. (**1998**): Therapeutic, toxic, and lethal concentrations of 73 drugs affecting respiratory system in human fluids. In *Journal of toxicology. Clinical toxicology* 36 (4), pp. 287–293. DOI: 10.3109/15563659809028023.

[81] Michaëlsson, Karl; Lithell, Hans; Vessby, Bengt; Melhus, Håkan (**2003**): Serum retinol levels and the risk of fracture. In *New England Journal of Medicine* 348 (4), pp. 287–294. DOI: 10.1056/NEJMoa021171.

[82] SCF (**2000**): Opinion of the Scientific Committee on Food on sucralose (Adopted by the SCF on 7 September 2000). SCF/CS/ADDS/EDUL/190 Final. Scientific Committee on Food (SCF). Available online at https://ec.europa.eu/food/sites/food/files/safety/docs/sci-com_scf_out68_en.pdf, updated on 12/9/2000, checked on 11/26/2020.

[83] Roberts, A.; Renwick, A. G.; Sims, J.; Snodin, D. J. (**2000**): Sucralose metabolism and pharmacokinetics in man. In *Food and Chemical Toxicology* 38, pp. 31–41. DOI: 10.1016/S0278-6915(00)00026-0.

[84] Baird, I.McLean; Shephard, N. W.; Merritt, R. J.; Hildick-Smith, G. (**2000**): Repeated dose study of sucralose tolerance in human subjects. In *Food and Chemical Toxicology* 38, pp. 123–129. DOI: 10.1016/S0278-6915(00)00035-1.

[85] Sylvetsky, Allison C.; Bauman, Viviana; Blau, Jenny E.; Garraffo, H. Martin; Walter, Peter J.; Rother, Kristina I. (**2017**): Plasma concentrations of sucralose in children and adults. In *Toxicological and environmental chemistry* 99 (3), pp. 535–542. DOI: 10.1080/02772248.2016.1234754.

[86] Panretin®, Stiefel Laboratories: Panretin(R) product information. Available online at https://www.accessdata.fda.gov/drugsatfda_docs/label/1999/20886lbl.pdf, checked on 11/25/2020.

[87] Schmitt-Hoffmann, A. H.; Roos, B.; Schoetzau, A.; Leese, P. T.; Meyer, I.; van de Wetering, J.; Kovacs, P. (**2012**): Oral alitretinoin: a review of the clinical pharmacokinetics and pharmacodynamics. In *Expert review of clinical pharmacology* 5 (4), pp. 373–388. DOI: 10.1586/ECP.12.26.

[88] Wiegand, Ulf-W.; Chou, Ruby C. (**1998**): Pharmacokinetics of acitretin and etretinate. In *Journal of the American Academy of Dermatology* 39 (2), S25-S33. DOI: 10.1016/s0190-9622(98)70441-4.

[89] Brindley, C. J. (**1989**): Overview of recent clinical pharmacokinetic studies with acitretin (Ro 10-1670, etretin). In *Dermatologica* 178 (2), pp. 79–87. DOI: 10.1159/000248397.

[90] Heath, Michael S.; Sahni, Dev R.; Curry, Zachary A.; Feldman, Steven R. (**2018**): Pharmacokinetics of tazarotene and acitretin in psoriasis. In *Expert opinion on drug metabolism & toxicology* 14 (9), pp. 919–927. DOI: 10.1080/17425255.2018.1515198.

[91] Hill, Christopher R.; Cole, Michael; Errington, Julie; Malik, Ghada; Boddy, Alan V.; Veal, Gareth J. (**2014**): Characterisation of the clinical pharmacokinetics of actinomycin D and the influence of ABCB1 pharmacogenetic variation on actinomycin D disposition in children with cancer. In *Clin Pharmacokinet* 53 (8), pp. 741–751. DOI: 10.1007/s40262-014-0153-2.

[92] Veal, Gareth J.; Cole, Michael; Errington, Julie; Parry, Annie; Hale, Juliet; Pearson, Andrew D. J. et al. (**2005**): Pharmacokinetics of dactinomycin in a pediatric patient population: a United Kingdom Children's Cancer Study Group Study. In *Clinical cancer research : an official journal of the American Association for Cancer Research* 11 (16), pp. 5893–5899. DOI: 10.1158/1078-0432.CCR-04-2546.

[93] Cilla, Donald D.; Whitfield, Lloyd R.; Gibson, Donald M.; Sedman, Allen J.; Posvar, Edward L. (**1996**): Multiple-dose pharmacokinetics, pharmacodynamics, and safety of atorvastatin, an inhibitor of HMG-CoA reductase, in healthy subjects. In *Clin Pharmacol Ther* 60 (6), pp. 687–695. DOI: 10.1016/S0009-9236(96)90218-0.

[94] Posvar, E. L.; Radulovic, L. L.; Cilla, D. D.; Whitfield, L. R.; Sedman, A. J. (**1996**): Tolerance and pharmacokinetics of single-dose atorvastatin, a potent inhibitor of HMG-CoA reductase, in healthy subjects. In *Journal of clinical pharmacology* 36 (8), pp. 728–731. DOI: 10.1002/j.1552-4604.1996.tb04242.x.

[95] Henck, J. W.; Craft, W. R.; Black, A.; Colgin, J.; Anderson, J. A. (**1998**): Pre- and postnatal toxicity of the HMG-CoA reductase inhibitor atorvastatin in rats. In *Toxicological sciences : an official journal of the Society of Toxicology* 41 (1), pp. 88–99. DOI: 10.1006/toxs.1997.2400.

[96] Nau, H.; Kuhnz, W.; Egger, H. J.; Rating, D.; Helge, H. (**1982**): Anticonvulsants during pregnancy and lactation. Transplacental, maternal and neonatal pharmacokinetics. In *Clinical Pharmacokinetics* 7 (6), pp. 508–543. DOI: 10.2165/00003088-198207060-00003.

[97] Pynnönen, S.; Kanto, J.; Sillanpää, M.; Erkkola, R. (**1977**): Carbamazepine: placental transport, tissue concentrations in foetus and newborn, and level in milk. In *Acta pharmacologica et toxicologica* 41 (3), pp. 244–253. DOI: 10.1111/j.1600-0773.1977.tb02145.x.

[98] Barpe, Deise Raquel; Rosa, Daniela Dornelles; Froehlich, Pedro Eduardo (**2010**): Pharmacokinetic evaluation of doxorubicin plasma levels in normal and overweight patients with breast cancer and simulation of dose adjustment by different indexes of body mass. In *European journal of pharmaceutical sciences : official journal of the European Federation for Pharmaceutical Sciences* 41 (3-4), pp. 458–463. DOI: 10.1016/j.ejps.2010.07.015.

[99] Twelves, C. J.; Dobbs, N. A.; Aldhous, M.; Harper, P. G.; Rubens, R. D.; Richards, M. A. (**1991**): Comparative pharmacokinetics of doxorubicin given by three different schedules with equal dose intensity in patients with breast cancer. In *Cancer chemotherapy and pharmacology* 28 (4), pp. 302–307. DOI: 10.1007/BF00685539.

[100] Greene, R. F.; Collins, J. M.; Jenkins, J. F.; Speyer, J. L.; Myers, C. E. (**1983**): Plasma pharmacokinetics of adriamycin and adriamycinol: implications for the design of in vitro experiments and treatment protocols. In *Cancer research* 43 (7), pp. 3417–3421.

[101] Rempel, Eugen; Hoelting, Lisa; Waldmann, Tanja; Balmer, Nina V.; Schildknecht, Stefan; Grinberg, Marianna et al. (**2015**): A transcriptome-based classifier to identify developmental toxicants by stem cell testing: design, validation and optimization for histone deacetylase inhibitors. In *Arch Toxicol* 89 (9), pp. 1599–1618. DOI: 10.1007/s00204-015-1573-y.

[102] Hayden, Frederick G.; Shindo, Nahoko (**2019**): Influenza virus polymerase inhibitors in clinical development. In *Current opinion in infectious diseases* 32 (2), pp. 176–186. DOI: 10.1097/QCO.0000000000000532.

[103] Madelain, Vincent; Nguyen, Thi Huyen Tram; Olivo, Anaelle; Lamballerie, Xavier de; Guedj, Jérémie; Taburet, Anne-Marie; Mentré, France (**2016**): Ebola Virus Infection: Review of the Pharmacokinetic and Pharmacodynamic Properties of Drugs Considered for Testing in Human Efficacy Trials. In *Clin Pharmacokinet* 55 (8), pp. 907–923. DOI: 10.1007/s40262-015-0364-1.

[104] Colburn, W. A.; Gibson, D. M.; Wiens, R. E.; Hanigan, J. J. (**1983**): Food increases the bioavailability of isotretinoin. In *Journal of clinical pharmacology* 23 (11-12), pp. 534–539. DOI: 10.1002/j.1552-4604.1983.tb01800.x.

[105] Rozman, Blaz (**2002**): Clinical pharmacokinetics of leflunomide. In *Clinical Pharmacokinetics* 41 (6), pp. 421–430. DOI: 10.2165/00003088-200241060-00003.

[106] Severus, W. E.; Kleindienst, N.; Seemüller, F.; Frangou, S.; Möller, H. J.; Greil, W. (**2008**): What is the optimal serum lithium level in the long-term treatment of bipolar disorder--a review? In *Bipolar disorders* 10 (2), pp. 231–237. DOI: 10.1111/j.1399-5618.2007.00475.x.

[107] Apotex Inc. (**2013**): Product Monograph Apo-Lithium Carbonate. Apotex Inc. Available online at https://pdf.hres.ca/dpd_pm/00023004.PDF, checked on 11/27/2020.

[108] Wesseloo, Richard; Wierdsma, André I.; van Kamp, Inge L.; Munk-Olsen, Trine; Hoogendijk, Witte J. G.; Kushner, Steven A.; Bergink, Veerle (**2017**): Lithium dosing strategies during pregnancy and the postpartum period. In *The British journal of psychiatry : the journal of mental science* 211 (1), pp. 31–36. DOI: 10.1192/bjp.bp.116.192799.

[109] Balis, F. M.; Holcenberg, J. S.; Poplack, D. G.; Ge, J.; Sather, H. N.; Murphy, R. F. et al. (**1998**): Pharmacokinetics and pharmacodynamics of oral methotrexate and mercaptopurine in children with lower risk acute lymphoblastic leukemia: a joint children's cancer group and pediatric oncology branch study. In *Blood* 92 (10), pp. 3569–3577.

[110] Kim, Byoung-Gwon; Jo, Eun-Mi; Kim, Gyeong-Yeon; Kim, Dae-Seon; Kim, Yu-Mi; Kim, Rock-Bum et al. (**2012**): Analysis of methylmercury concentration in the blood of Koreans by using cold vapor atomic fluorescence spectrophotometry. In *Annals of laboratory medicine* 32 (1), pp. 31–37. DOI: 10.3343/alm.2012.32.1.31.

[111] Mortensen, Mary E.; Caudill, Samuel P.; Caldwell, Kathleen L.; Ward, Cynthia D.; Jones, Robert L. (**2014**): Total and methyl mercury in whole blood measured for the first time in the U.S. population: NHANES 2011-2012. In *Environmental Research* 134, pp. 257–264. DOI: 10.1016/j.envres.2014.07.019.

[112] van Veggel, Mathilde; Westerman, Elsbeth; Hamberg, Paul (**2018**): Clinical Pharmacokinetics and Pharmacodynamics of Panobinostat. In *Clin Pharmacokinet* 57 (1), pp. 21–29. DOI: 10.1007/s40262-017-0565-x.

[113] Kaye, C. M.; Haddock, R. E.; Langley, P. F.; Mellows, G.; Tasker, T. C.; Zussman, B. D.; Greb, W. H. (**1989**): A review of the metabolism and pharmacokinetics of paroxetine in man. In *Acta psychiatrica Scandinavica. Supplementum* 350, pp. 60–75. DOI: 10.1111/j.1600-0447.1989.tb07176.x.

[114] Koch, S.; Jäger-Roman, E.; Lösche, G.; Nau, H.; Rating, D.; Helge, H. (**1996**): Antiepileptic drug treatment in pregnancy: drug side effects in the neonate and neurological outcome. In *Acta paediatrica (Oslo, Norway : 1992)* 85 (6), pp. 739–746. DOI: 10.1111/j.1651-2227.1996.tb14137.x.

[115] Teo, Steve K.; Colburn, Wayne A.; Tracewell, William G.; Kook, Karin A.; Stirling, David I.; Jaworsky, Markian S. et al. (**2004**): Clinical pharmacokinetics of thalidomide. In *Clinical Pharmacokinetics* 43 (5), pp. 311–327. DOI: 10.2165/00003088-200443050-00004.

[116] Lu, K.; Yap, H. Y.; Loo, T. L. (**1983**): Clinical pharmacokinetics of vinblastine by continuous intravenous infusion. In *Cancer research* 43 (3), pp. 1405–1408.

[117] Nelson, R. L.; Dyke, R. W.; Root, M. A. (**1980**): Comparative pharmacokinetics of vindesine, vincristine and vinblastine in patients with cancer. In *Cancer Treatment Reviews* 7, pp. 17–24. DOI: 10.1016/S0305-7372(80)80003-X.

[118] Links, M.; Watson, S.; Lethlean, K.; Aherne, W.; Kirsten, F.; Clarke, S. et al. (**1999**): Vinblastine pharmacokinetics in patients with non-small cell lung cancer given cisplatin. In *Cancer investigation* 17 (7), pp. 479–485. DOI: 10.3109/07357909909032857.

[119] van Tellingen, O.; Beijnen, J. H.; Nooijen, W. J.; Bult, A. (**1993**): Plasma pharmacokinetics of vinblastine and the investigational Vinca alkaloid N-(deacetyl-O-4-vinblastoyl-23)-L-ethyl isoleucinate in mice as determined by high-performance liquid chromatography. In *Cancer research* 53 (9), pp. 2061–2065.

[120] van Calsteren, Kristel; Hartmann, Dieter; van Aerschot, Leen; Verbesselt, Rene; van Bree, Rieta; D'Hooge, Rudi; Amant, Frédéric (**2009**): Vinblastine and doxorubicin administration to pregnant mice affects brain development and behaviour in the offspring. In *NeuroToxicology* 30 (4), pp. 647–657. DOI: 10.1016/j.neuro.2009.04.009.

[121] Abou-Alfa, Ghassan K.; Lewis, Lionel D.; LoRusso, Patricia; Maitland, Michael; Chandra, Priya; Cheeti, Sravanthi et al. (**2017**): Pharmacokinetics and safety of vismodegib in patients with advanced solid malignancies and hepatic impairment. In *Cancer chemotherapy and pharmacology* 80 (1), pp. 29–36. DOI: 10.1007/s00280-017-3315-8.

[122] Graham, Richard A.; Hop, Cornelis E. C. A.; Borin, Marie T.; Lum, Bert L.; Colburn, Dawn; Chang, Ilsung et al. (**2012**): Single and multiple dose intravenous and oral pharmacokinetics of the hedgehog pathway inhibitor vismodegib in healthy female subjects. In *British Journal of Clinical Pharmacology* 74 (5), pp. 788–796. DOI: 10.1111/j.1365-2125.2012.04281.x.

[123] Iwamoto, Marian; Friedman, Evan J.; Sandhu, Punam; Agrawal, Nancy G. B.; Rubin, Eric H.; Wagner, John A. (**2013**): Clinical pharmacology profile of vorinostat, a histone deacetylase inhibitor. In *Cancer chemotherapy and pharmacology* 72 (3), pp. 493–508. DOI: 10.1007/s00280-013-2220-z.

[124] Pacifici, G. M. (**2006**): Placental transfer of antibiotics administered to the mother: a review. In *International journal of clinical pharmacology and therapeutics* 44 (2), pp. 57–63. DOI: 10.5414/cpp44057.

[125] Yoo, S. D.; Axelson, J. E.; Taylor, S. M.; Rurak, D. W. (**1986**): Placental transfer of diphenhydramine in chronically instrumented pregnant sheep. In *Journal of pharmaceutical sciences* 75 (7), pp. 685–687. DOI: 10.1002/jps.2600750714.

[126] Slikker, W., Jr.; Bailey, J.R.; Holder, C. L.; Lipe, G. L. (**1987**): Transplacental disposition of doxylamine succinate in the late-term rhesus monkey. In Heinz Nau (Ed.): Pharmacokinetics in teratogenesis, vol. 1. Boca Raton, Fl.: CRC Pr, pp. 193–202.

[127] Henderson, G. I.; Perez, T.; Schenker, S.; Mackins, J.; Antony, A. C. (**1995**): Maternal-to-fetal transfer of 5-methyltetrahydrofolate by the perfused human placental cotyledon: evidence for a concentrative role by placental folate receptors in fetal folate delivery. In *The Journal of laboratory and clinical medicine* 126 (2), pp. 184–203.

[128] Hutson, Janine R.; Stade, Brenda; Lehotay, Denis C.; Collier, Christine P.; Kapur, Bhushan M. (**2012**): Folic acid transport to the human fetus is decreased in pregnancies with chronic alcohol exposure. In *PLoS ONE* 7 (5), e38057. DOI: 10.1371/journal.pone.0038057.

[129] Carr, E. A.; Beierwaltes, W. H.; Raman, G.; Dodson, V. N.; Tanton, J.; Betts, J. S.; Stambaugh, R. A. (**1959**): The effect of maternal thyroid function of fetal thyroid function and development. In *The Journal of clinical endocrinology and metabolism* 19 (1), pp. 1–18. DOI: 10.1210/jcem-19-1-1.

[130] Burrow, G. N.; Fisher, D. A.; Larsen, P. R. (**1994**): Maternal and fetal thyroid function. In *The New England journal of medicine* 331 (16), pp. 1072–1078. DOI: 10.1056/NEJM199410203311608.

[131] Dancis, J.; Levitz, M.; Katz, J.; Wilson, D.; Blaner, W. S.; Piantedosi, R.; Goodman, D. S. (**1992**): Transfer and metabolism of retinol by the perfused human placenta. In *Pediatric research* 32 (2), pp. 195–199. DOI: 10.1203/00006450-199208000-00014.

[132] Torma, H.; Vahlquist, A. (**1986**): Uptake of vitamin A and retinol-binding protein by human placenta in vitro. In *Placenta* 7 (4), pp. 295–305. DOI: 10.1016/S0143-4004(86)80147-3.

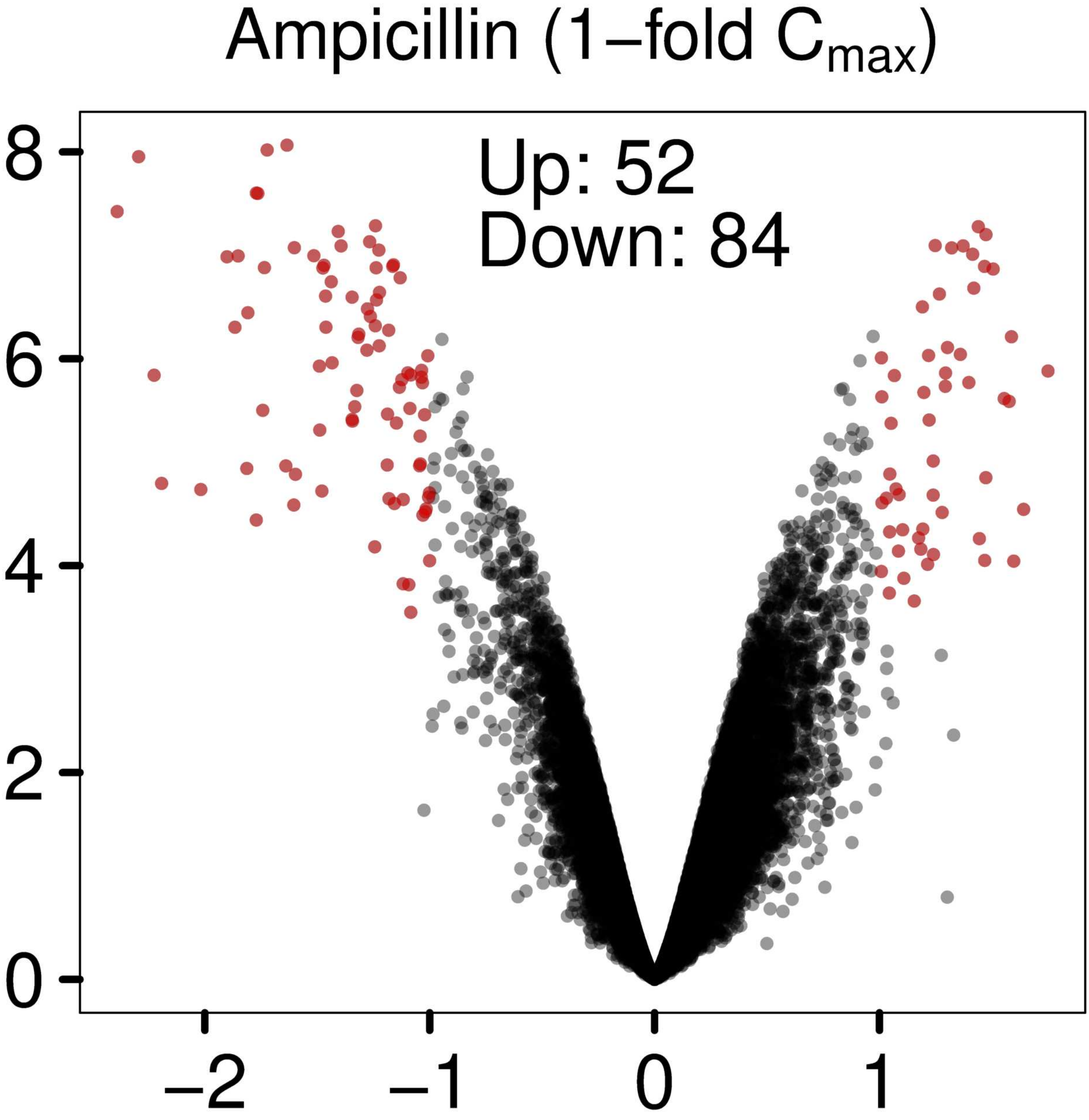

Ampicillin (1–fold C$_{max}$)
Up: 52
Down: 84
8
6
4
2
0
–2
–1
0
1

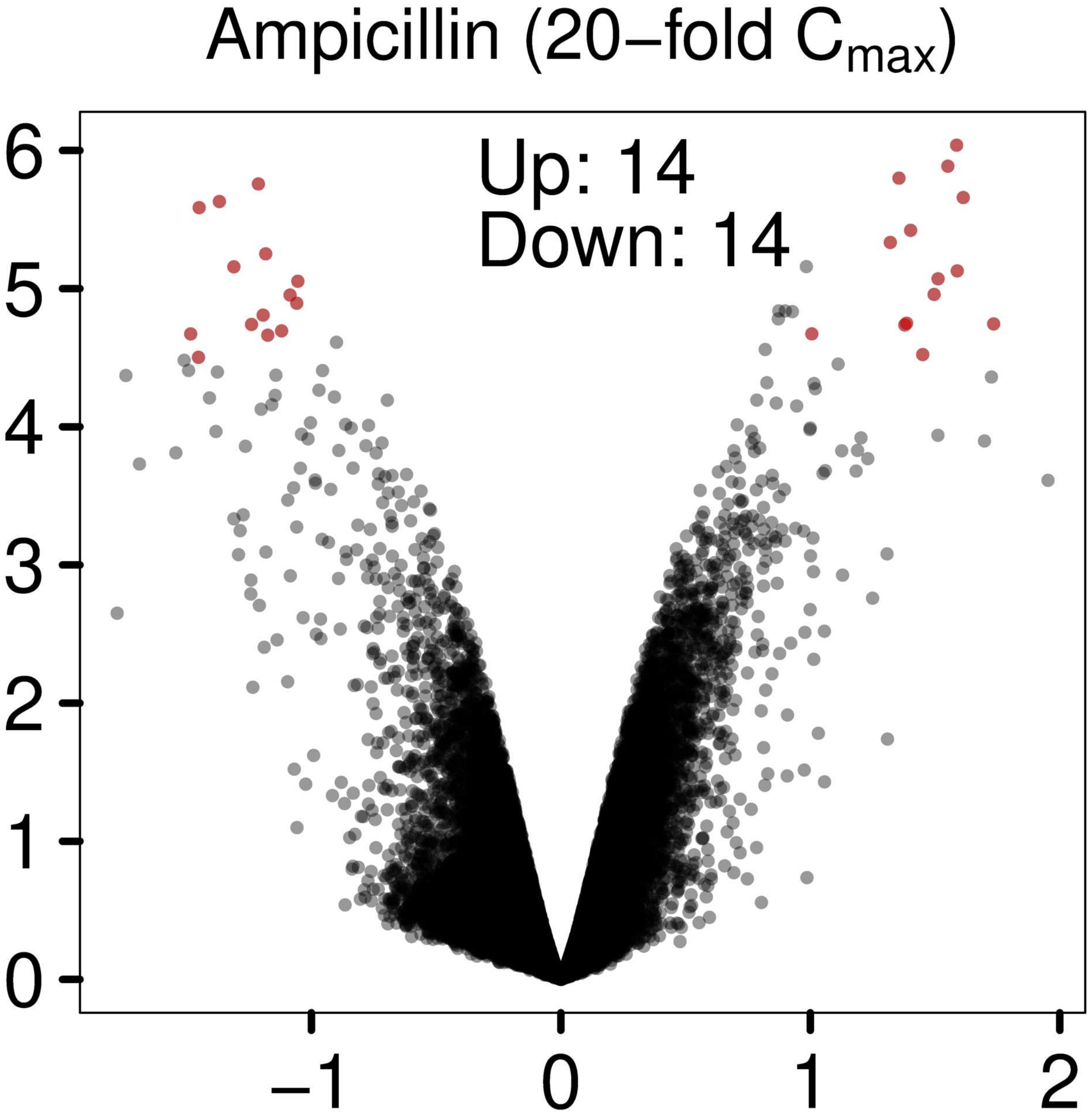

Ampicillin (20−fold C_{max})
Up: 14
Down: 14
6
5
4
3
2
1
0
−1
0
1
2

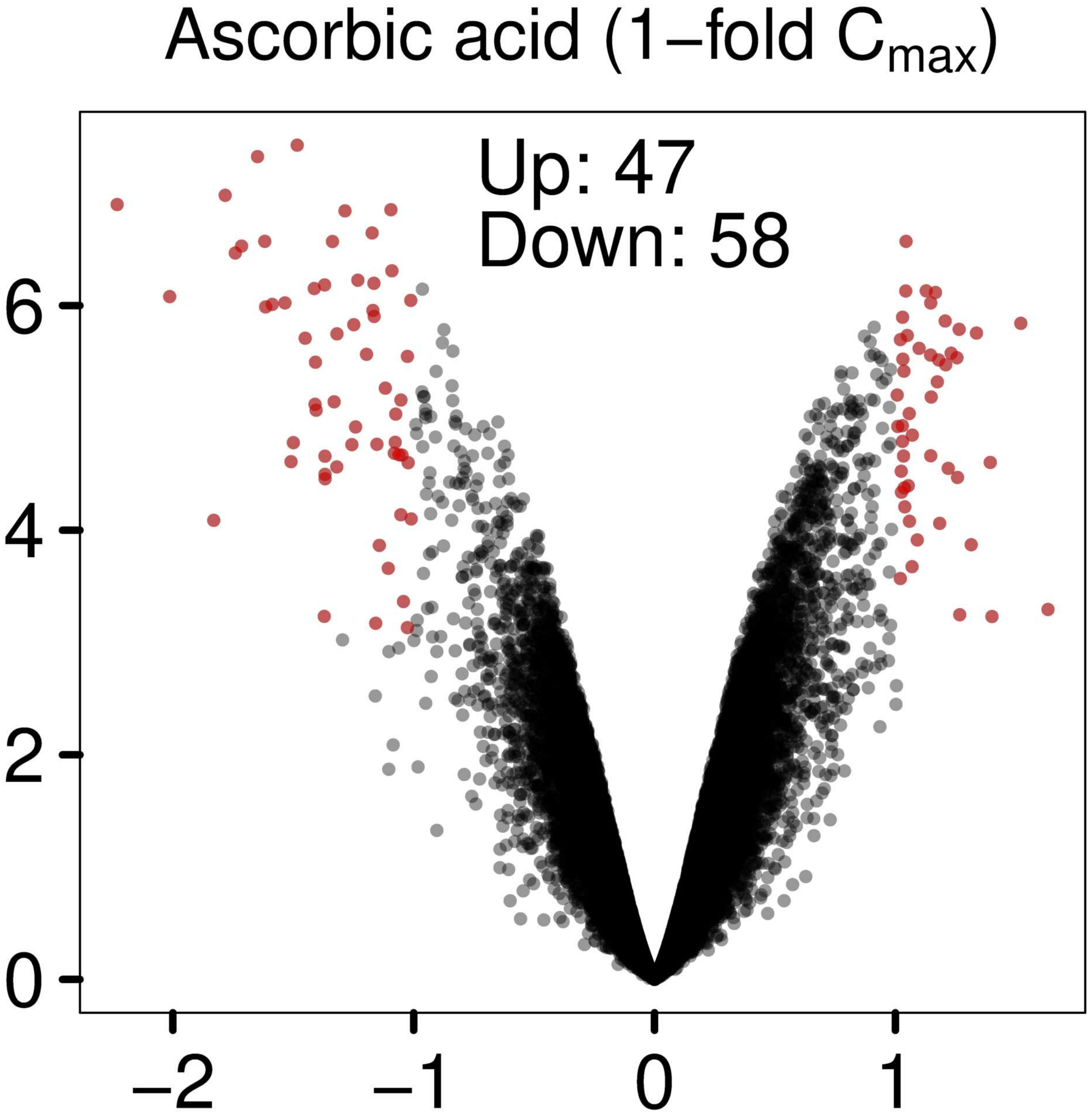

Ascorbic acid (1–fold C_{max})
Up: 47
Down: 58
6
4
2
0
–2
–1
0
1

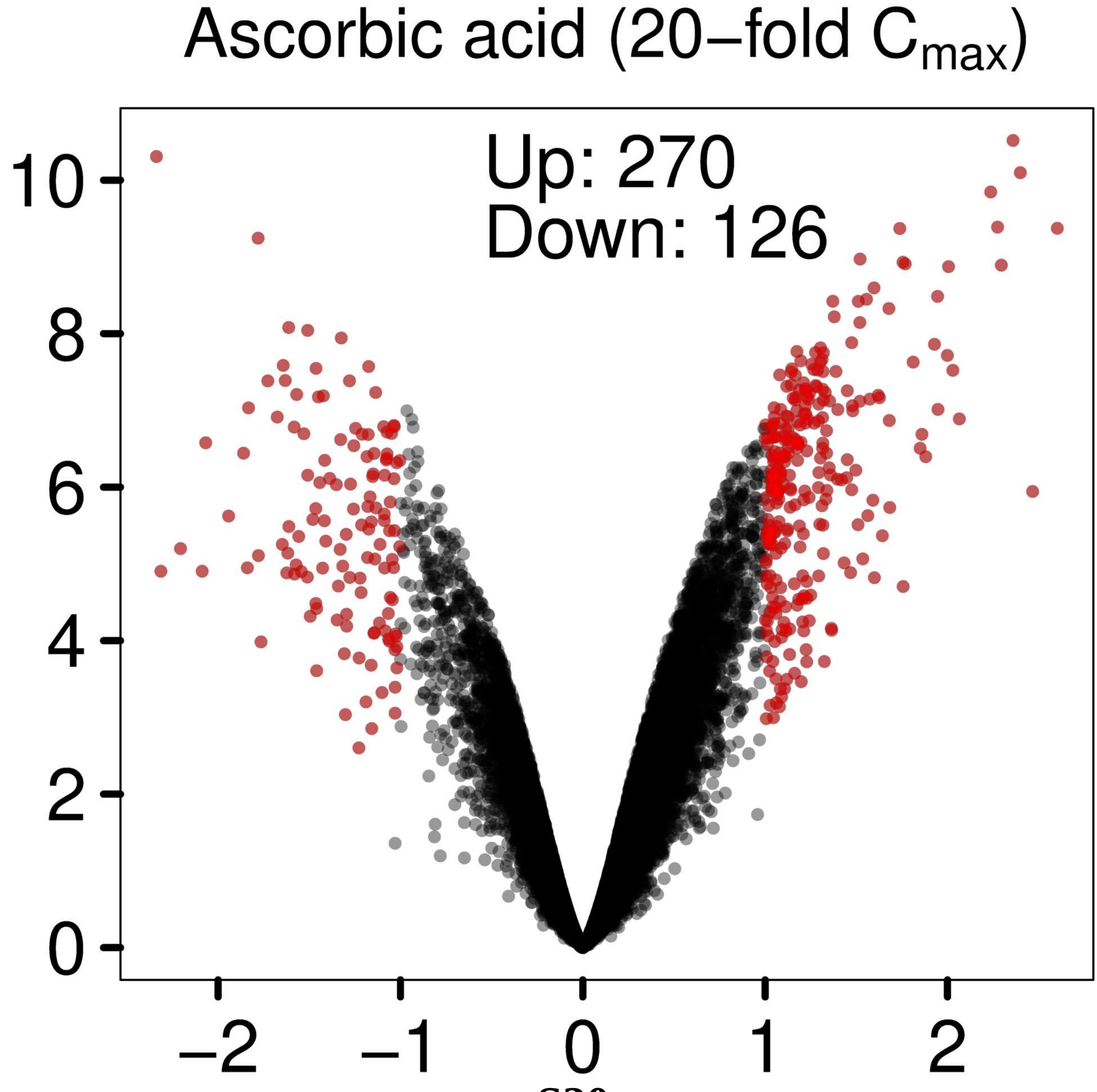

S30

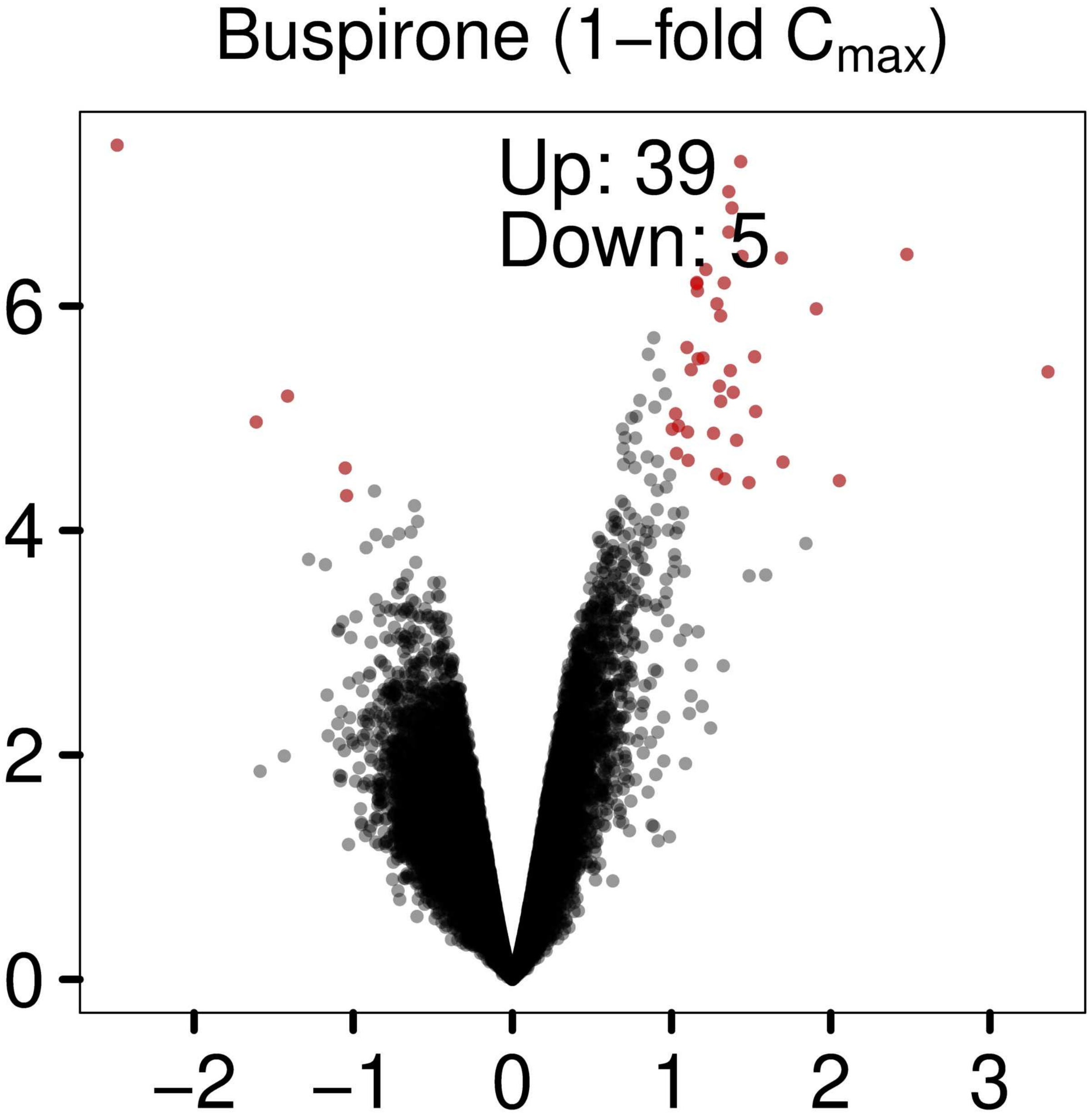

Buspirone (1−fold C_{max})
Up: 39
Down: 5
6
4
2
0
−2
−1
0
1
2
3

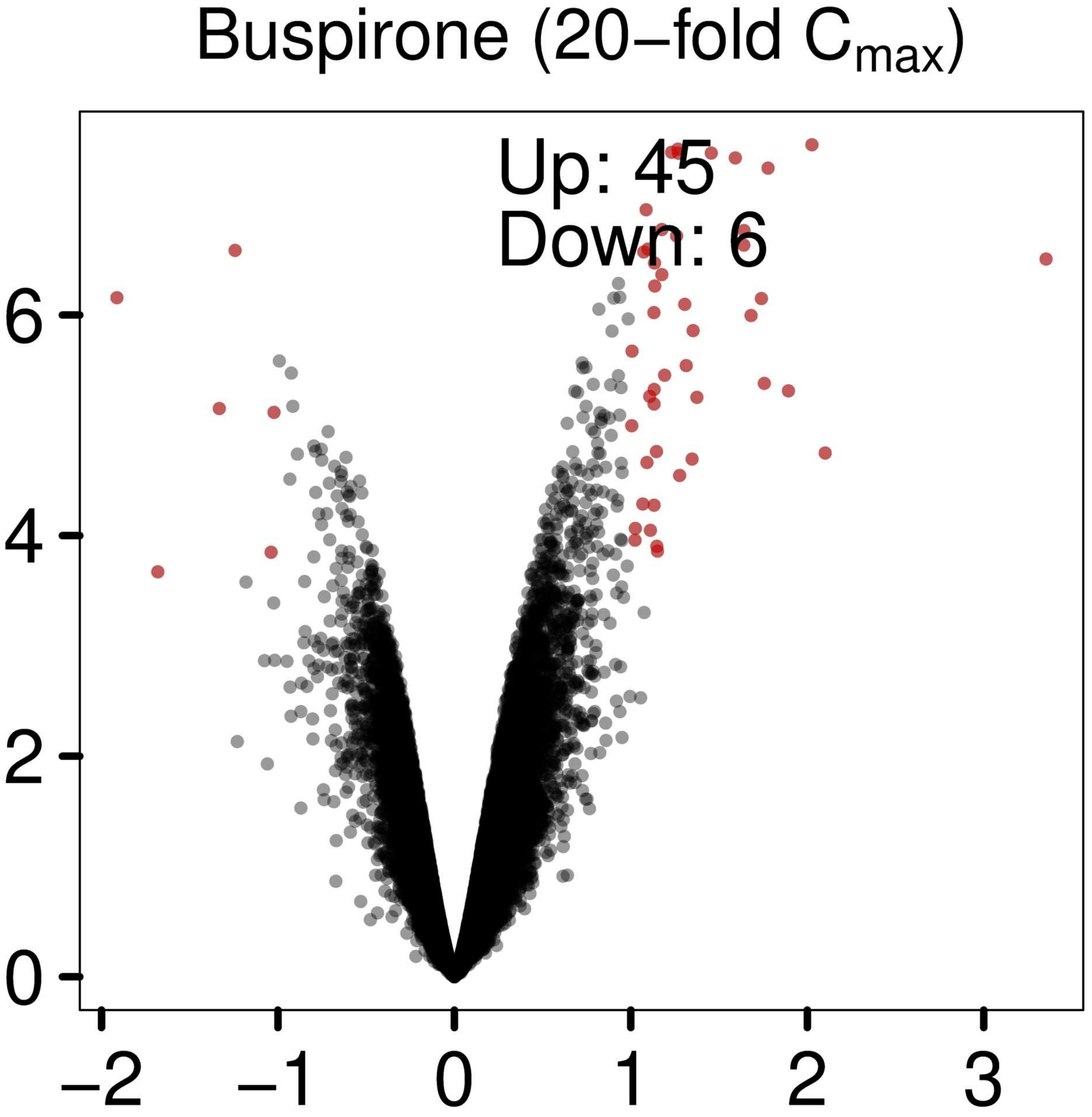
Buspirone (20–fold C_{max})
Up: 45
Down: 6
6
4
2
0
–2
–1
0
1
2
3

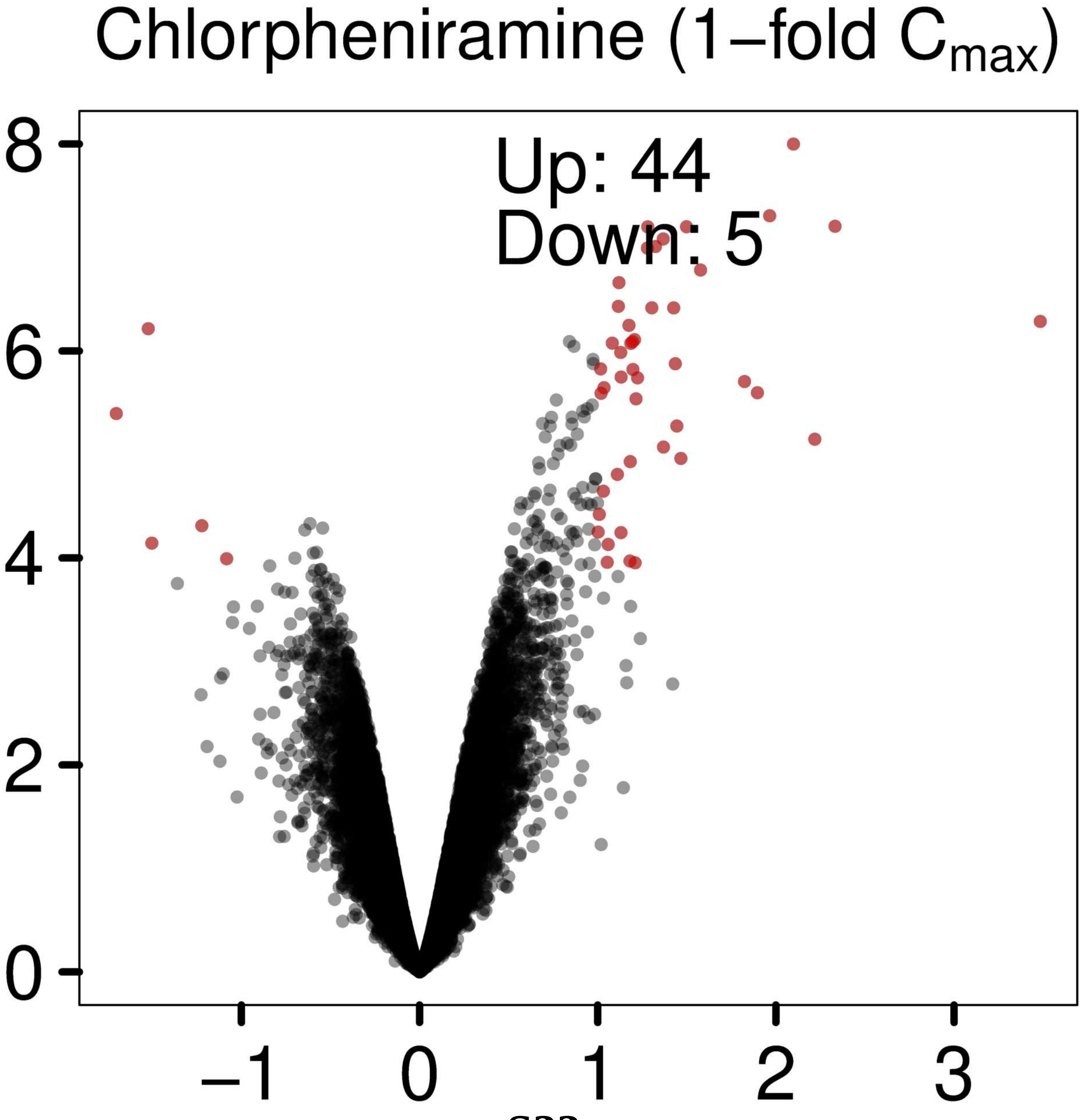

S33

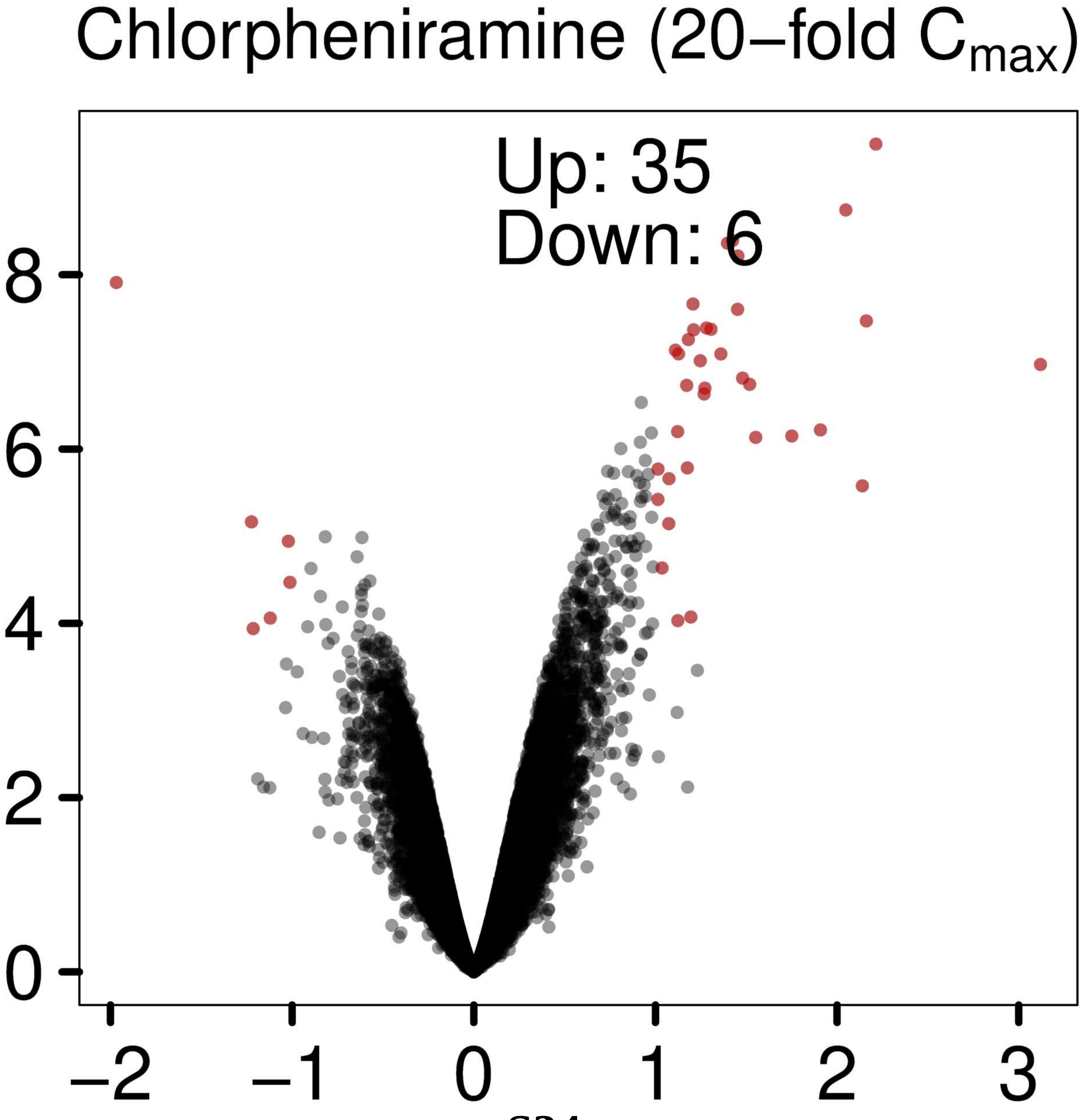

Chlorpheniramine (20–fold C_{max})
Up: 35
Down: 6
8
6
4
2
0
−2
−1
0
1
2
3

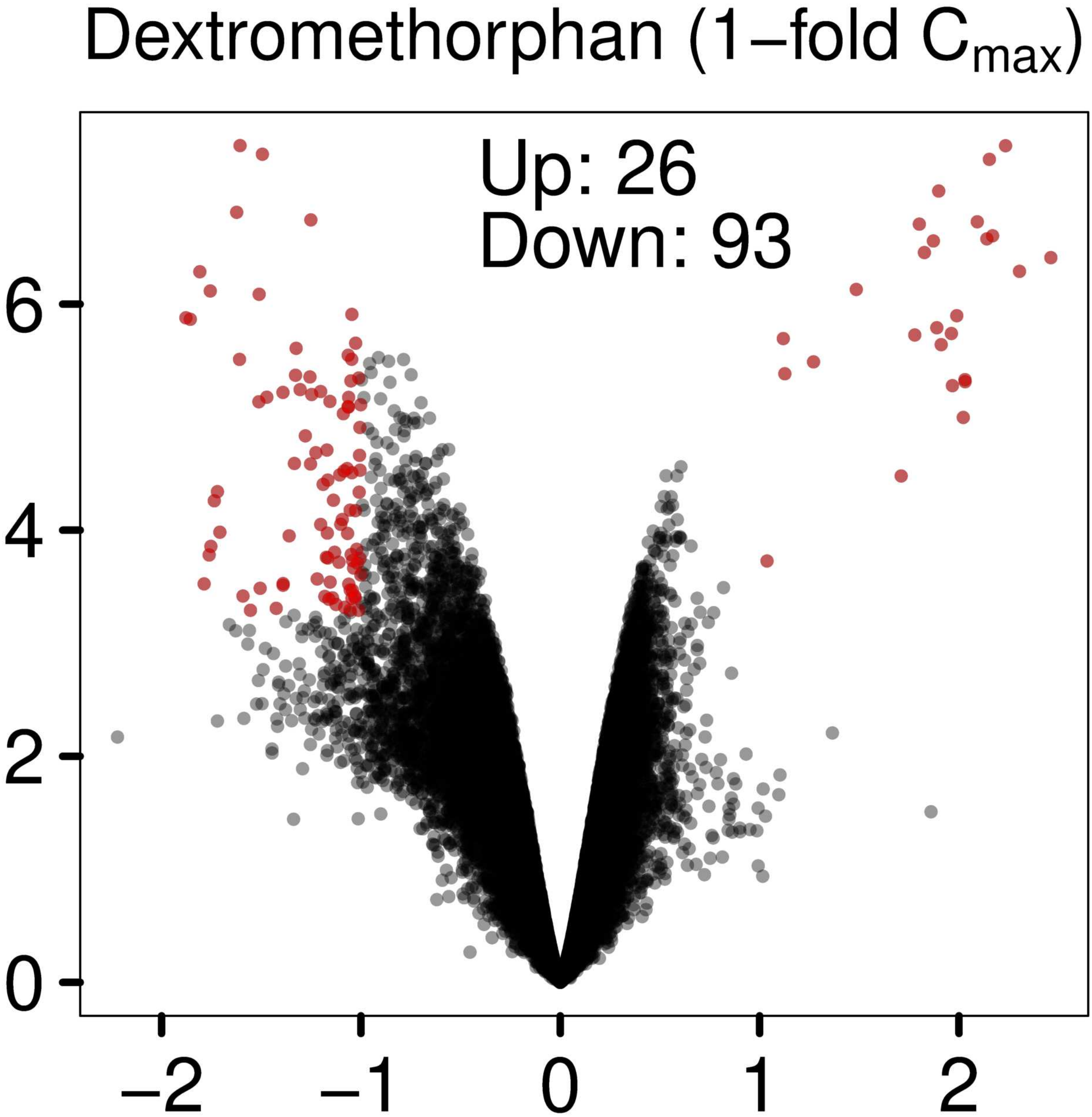
Dextromethorphan (1–fold C_{max})
Up: 26
Down: 93
6
4
2
0
–2
–1
0
1
2

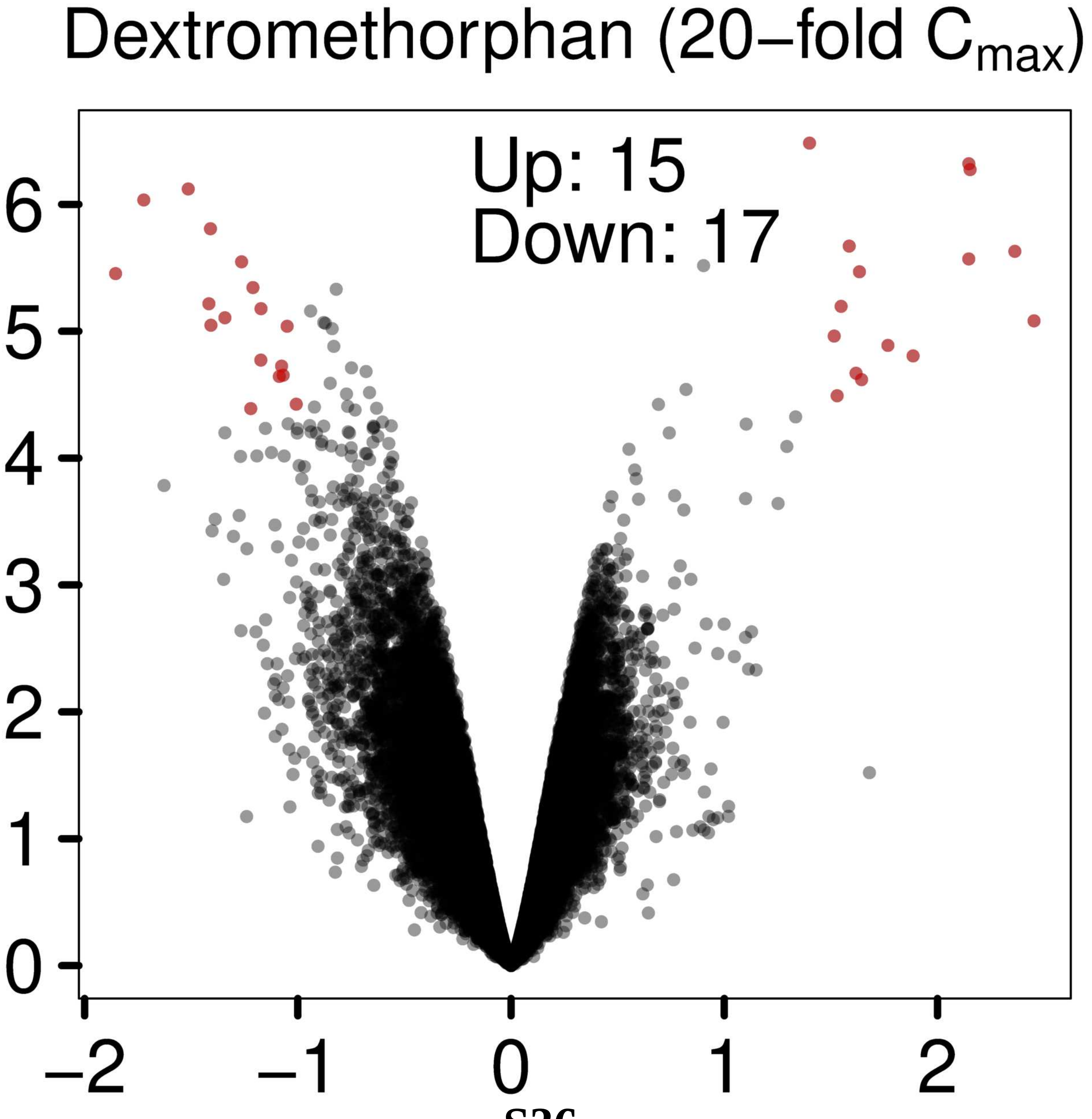
Dextromethorphan (20−fold C_{max})
Up: 15
Down: 17
6
5
4
3
2
1
0
−2
−1
0
1
2

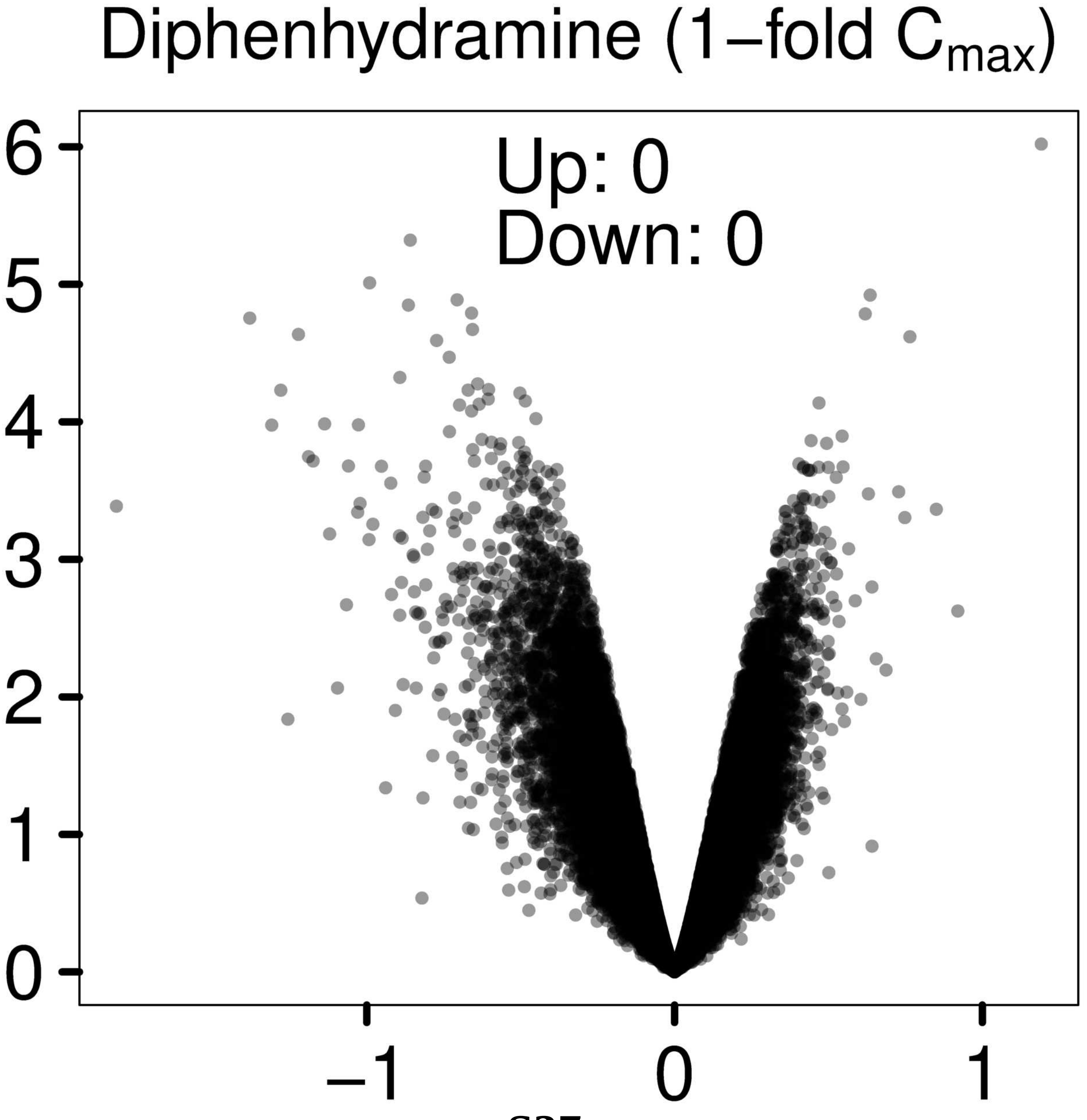

Diphenhydramine (1−fold C_{max})
Up: 0
Down: 0
6
5
4
3
2
1
0
−1
0
1

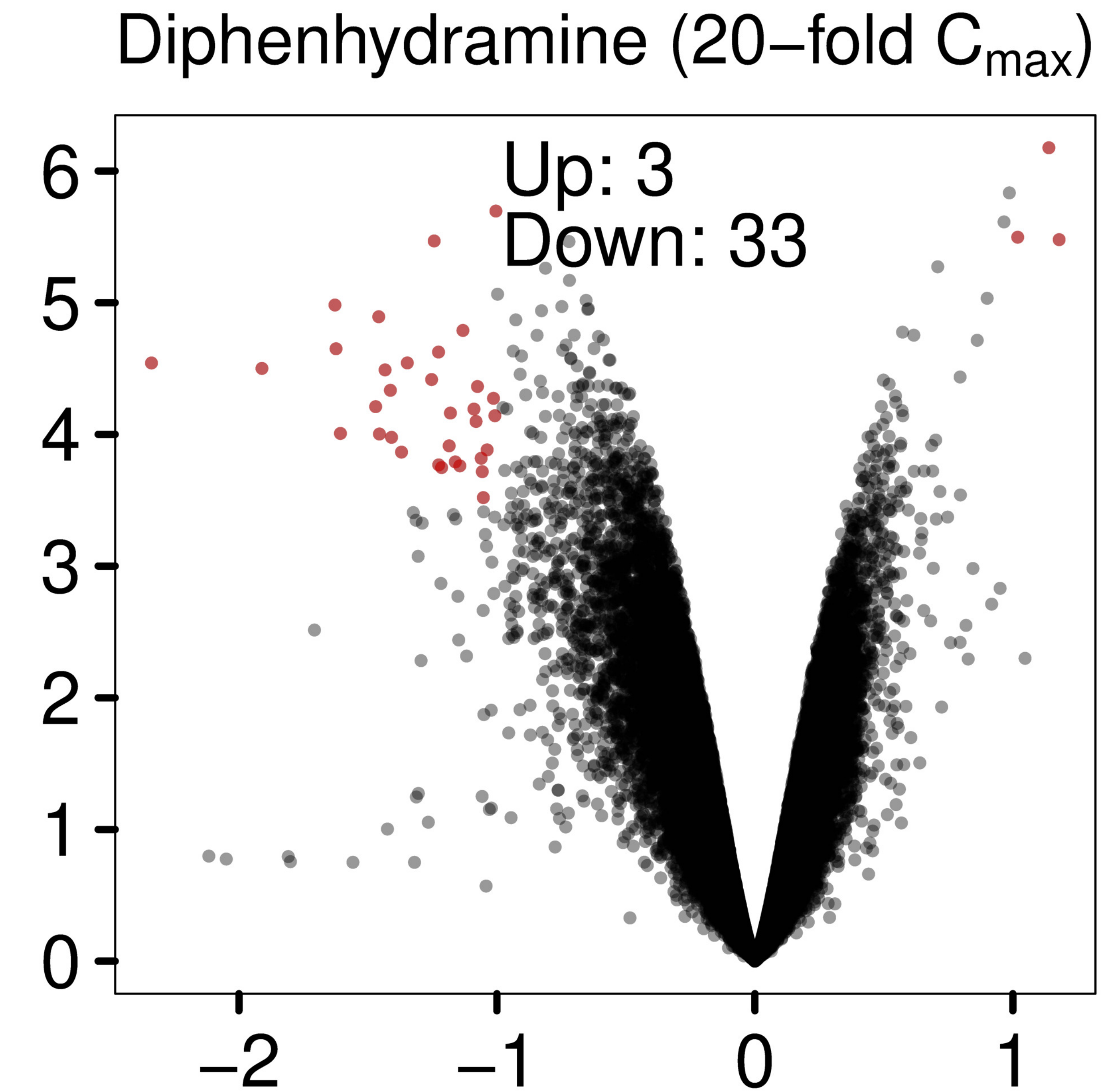

Diphenhydramine (20−fold C_{max})
Up: 3
Down: 33
6
5
4
3
2
1
0
−2
−1
0
1

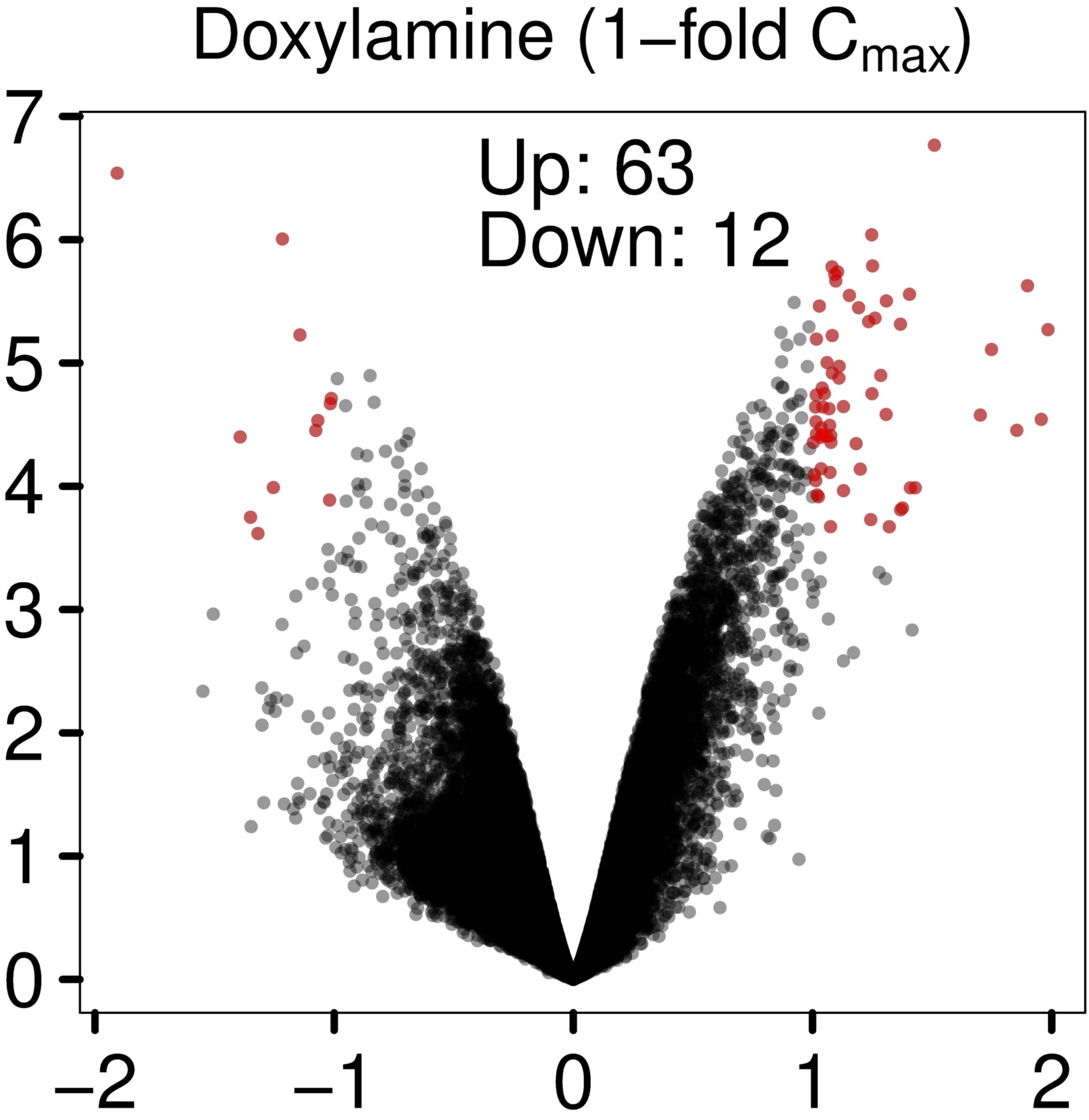
Doxylamine (1–fold C$_{max}$)
Up: 63
Down: 12
7
6
5
4
3
2
1
0
–2
–1
0
1
2

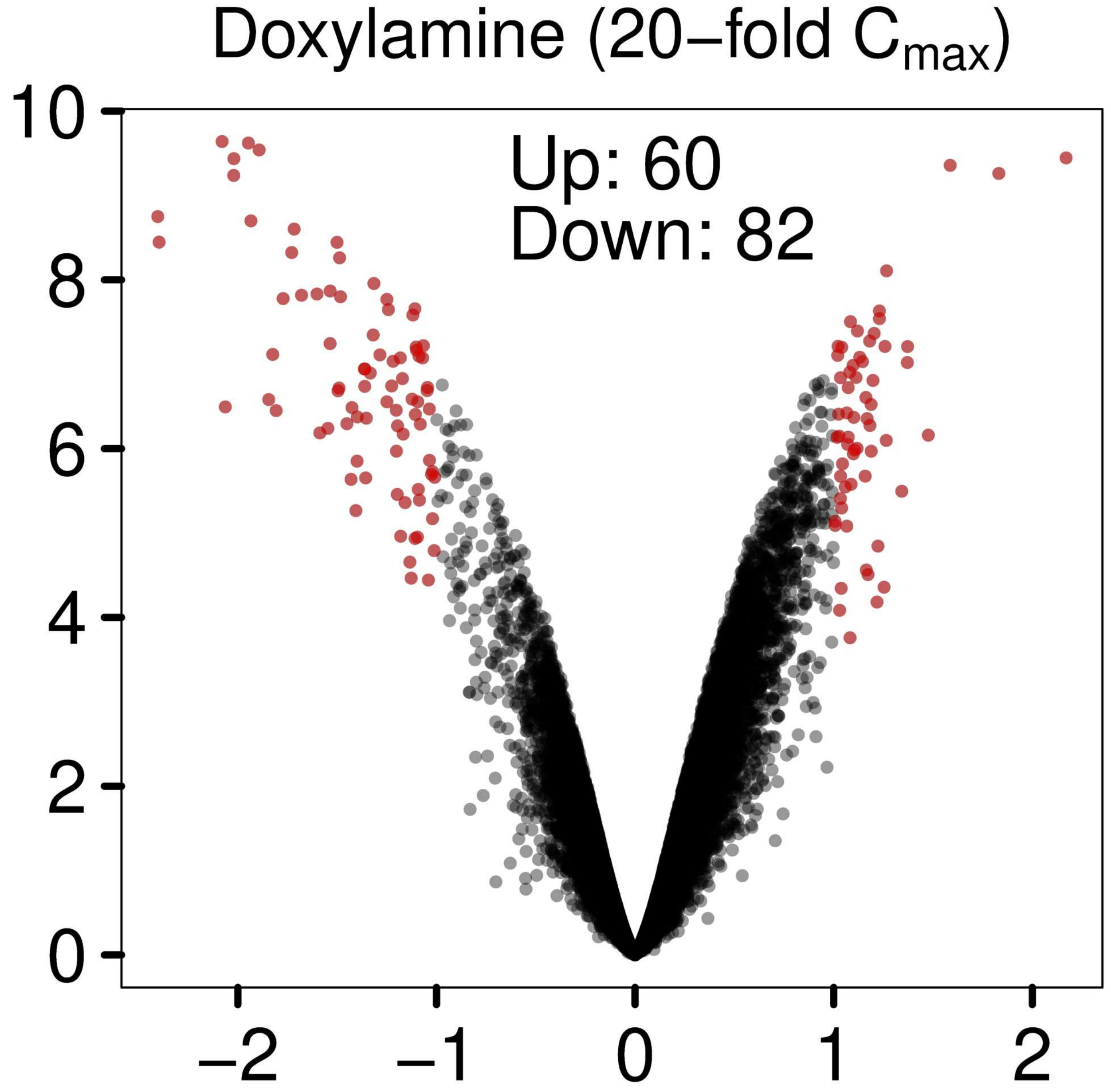

S40

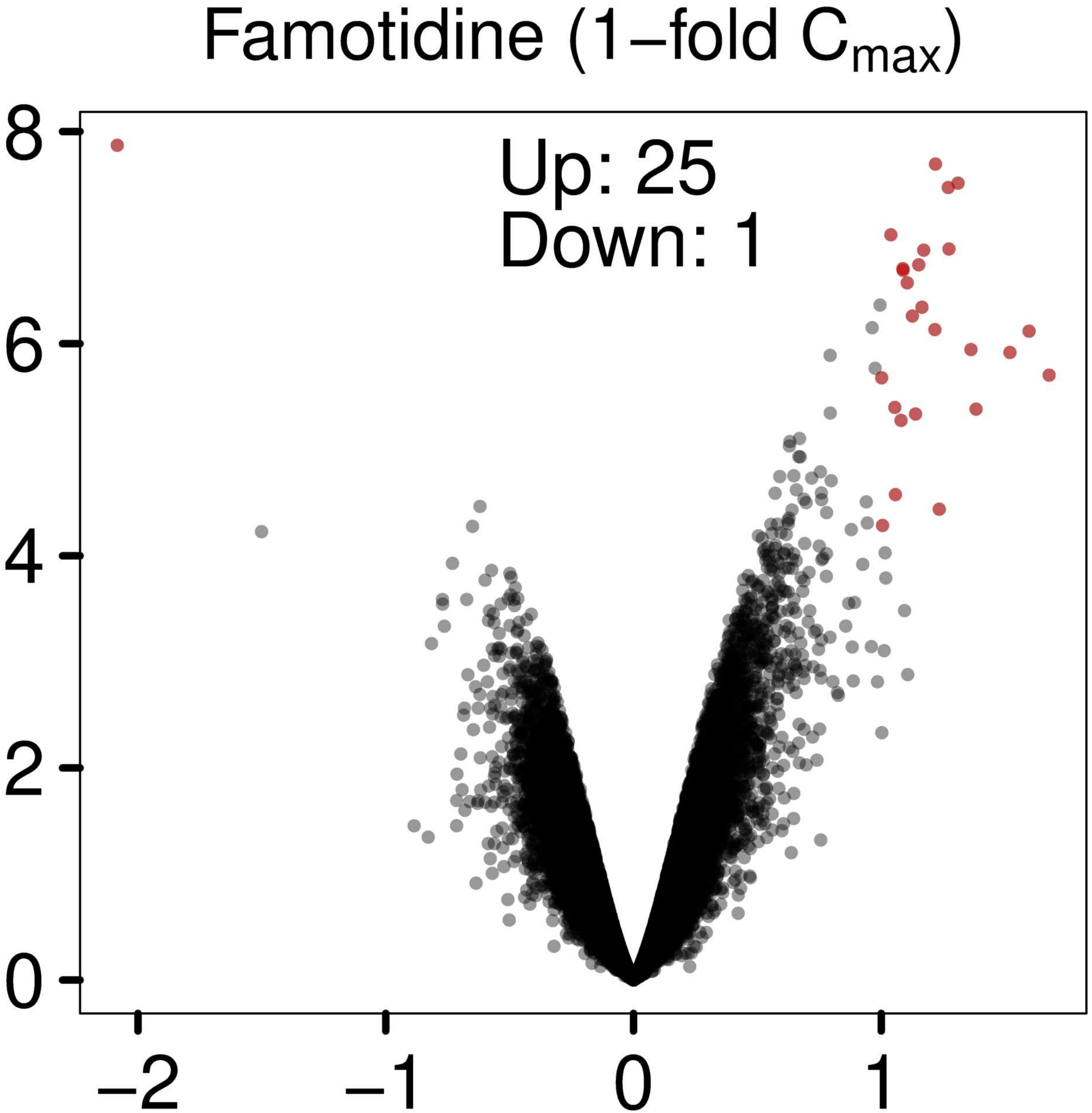

Famotidine (1−fold C_{max})
Up: 25
Down: 1
8
6
4
2
0
−2
−1
0
1

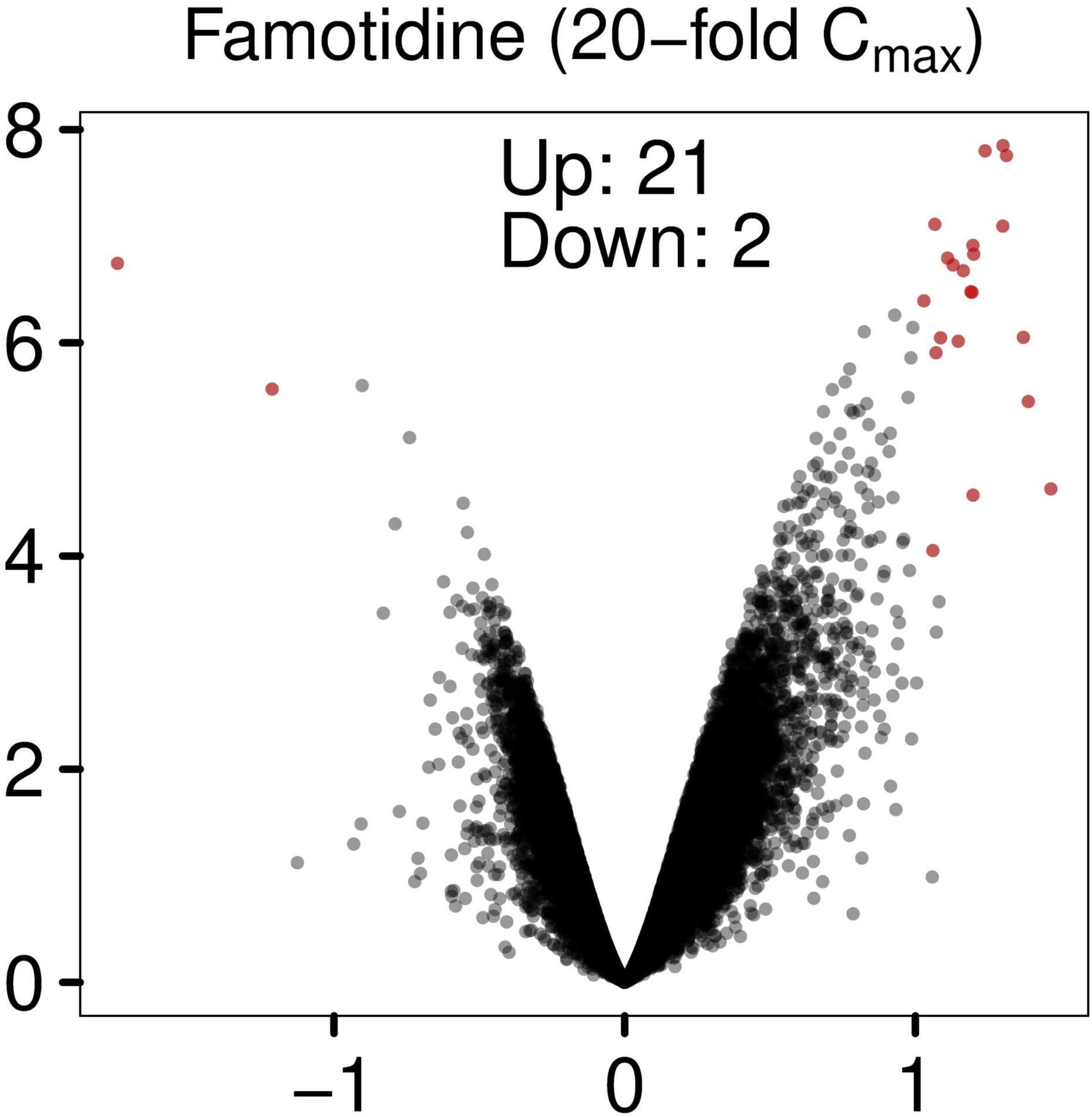

Famotidine (20−fold C_max)
Up: 21
Down: 2
8
6
4
2
0
−1
0
1

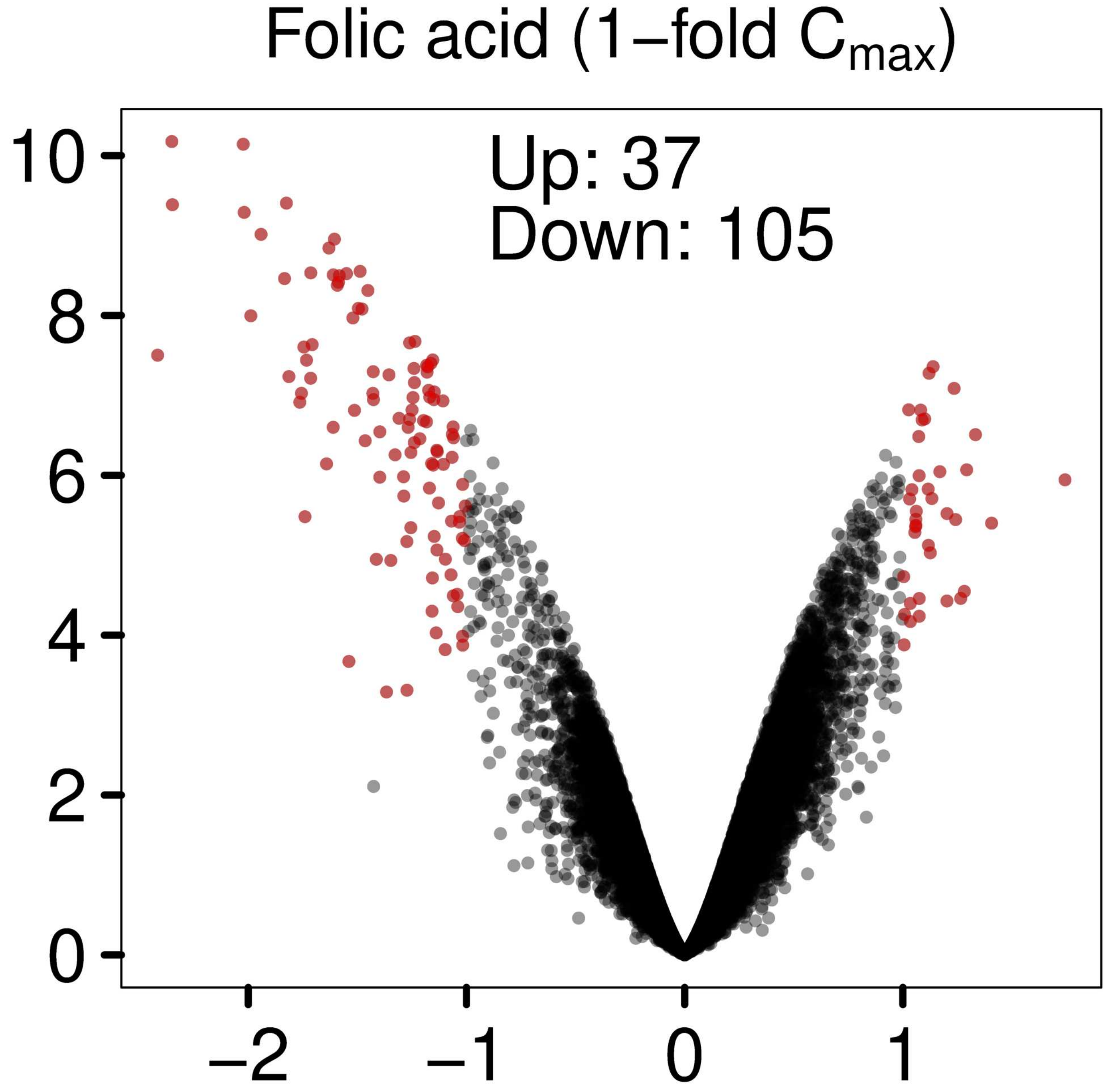

S43

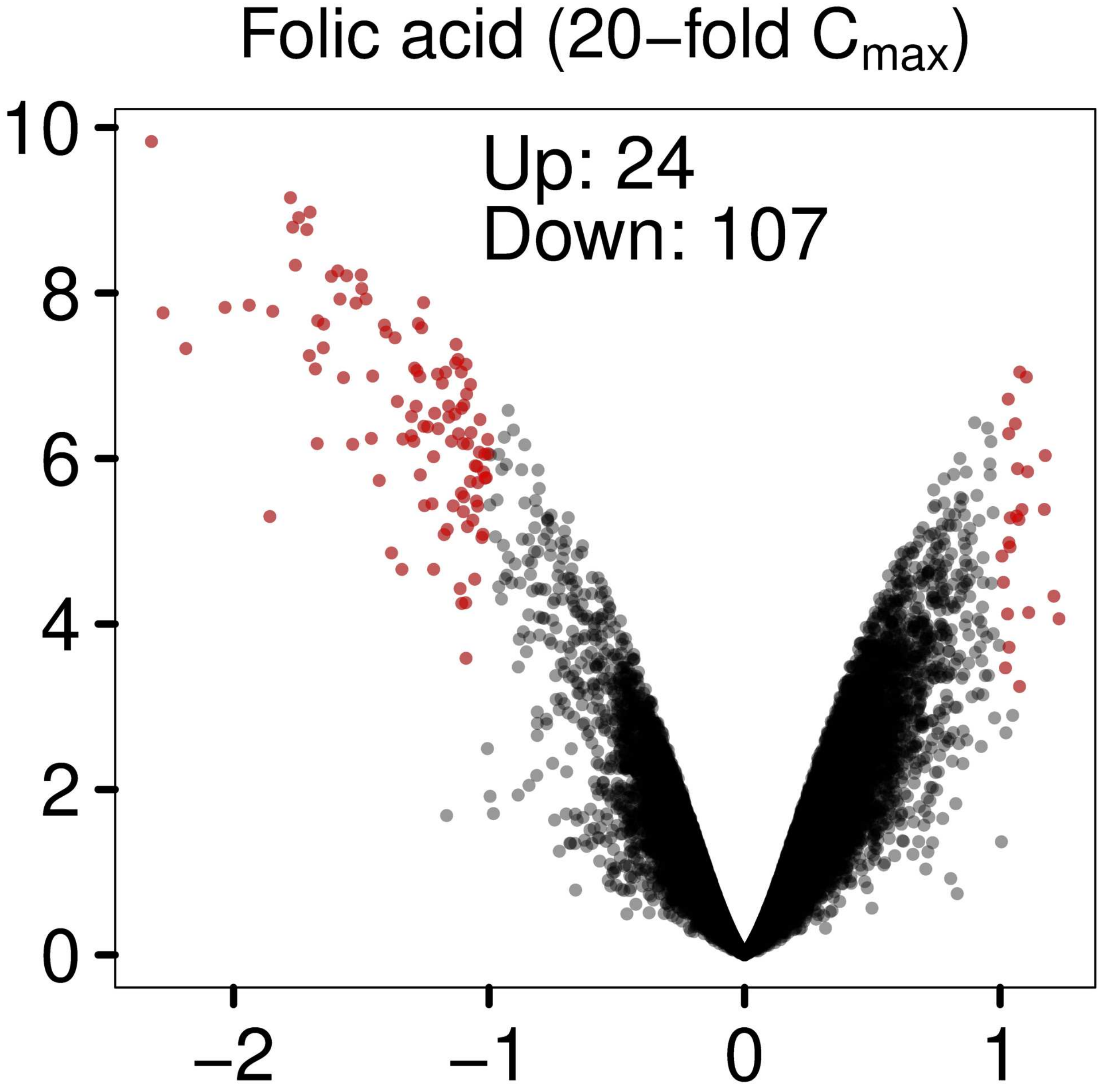

Folic acid (20–fold C_{max})
Up: 24
Down: 107
10
8
6
4
2
0
–2
–1
0
1

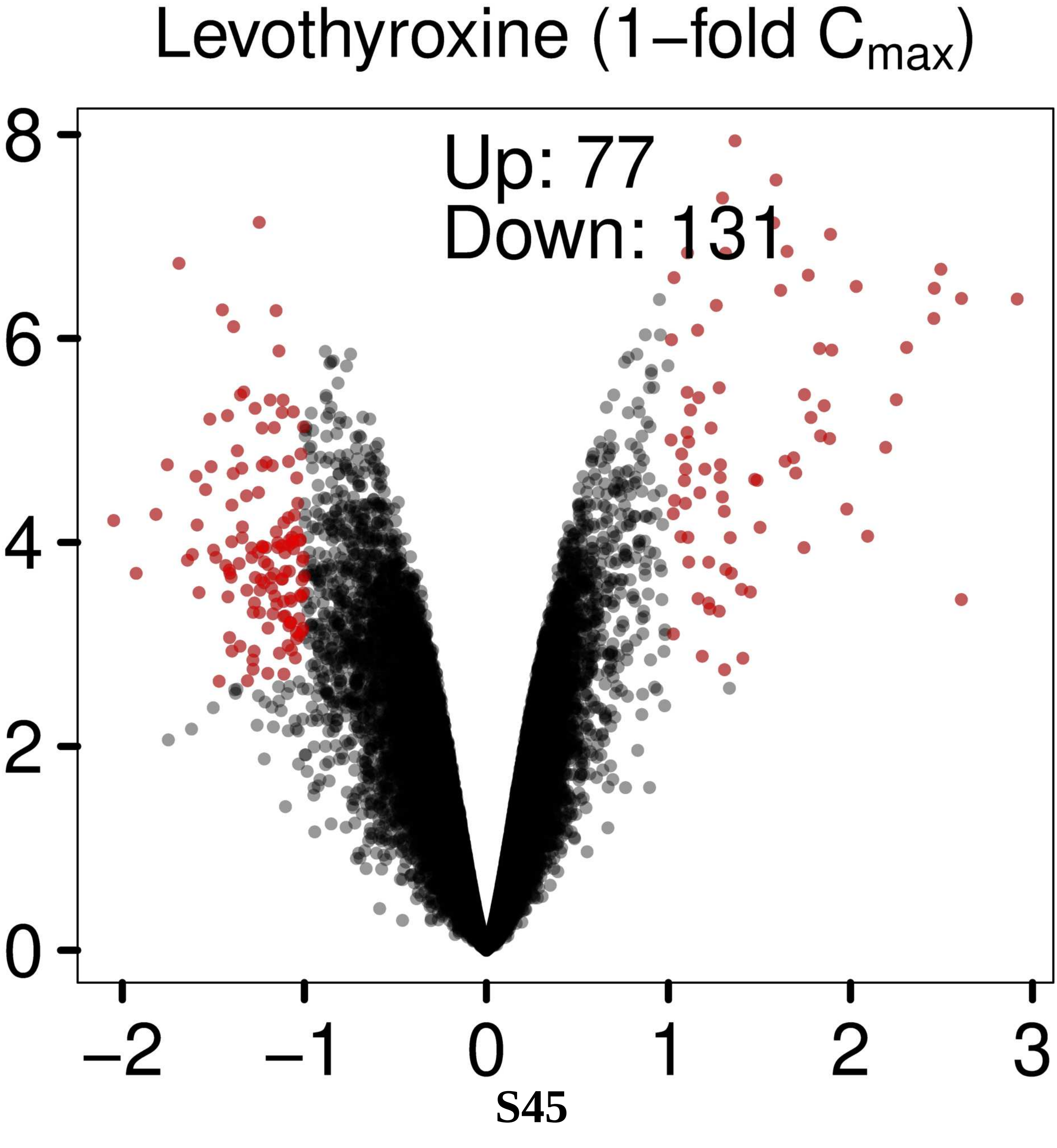

Levothyroxine (1−fold C_{max})
Up: 77
Down: 131
8
6
4
2
0
−2
−1
0
1
2
3

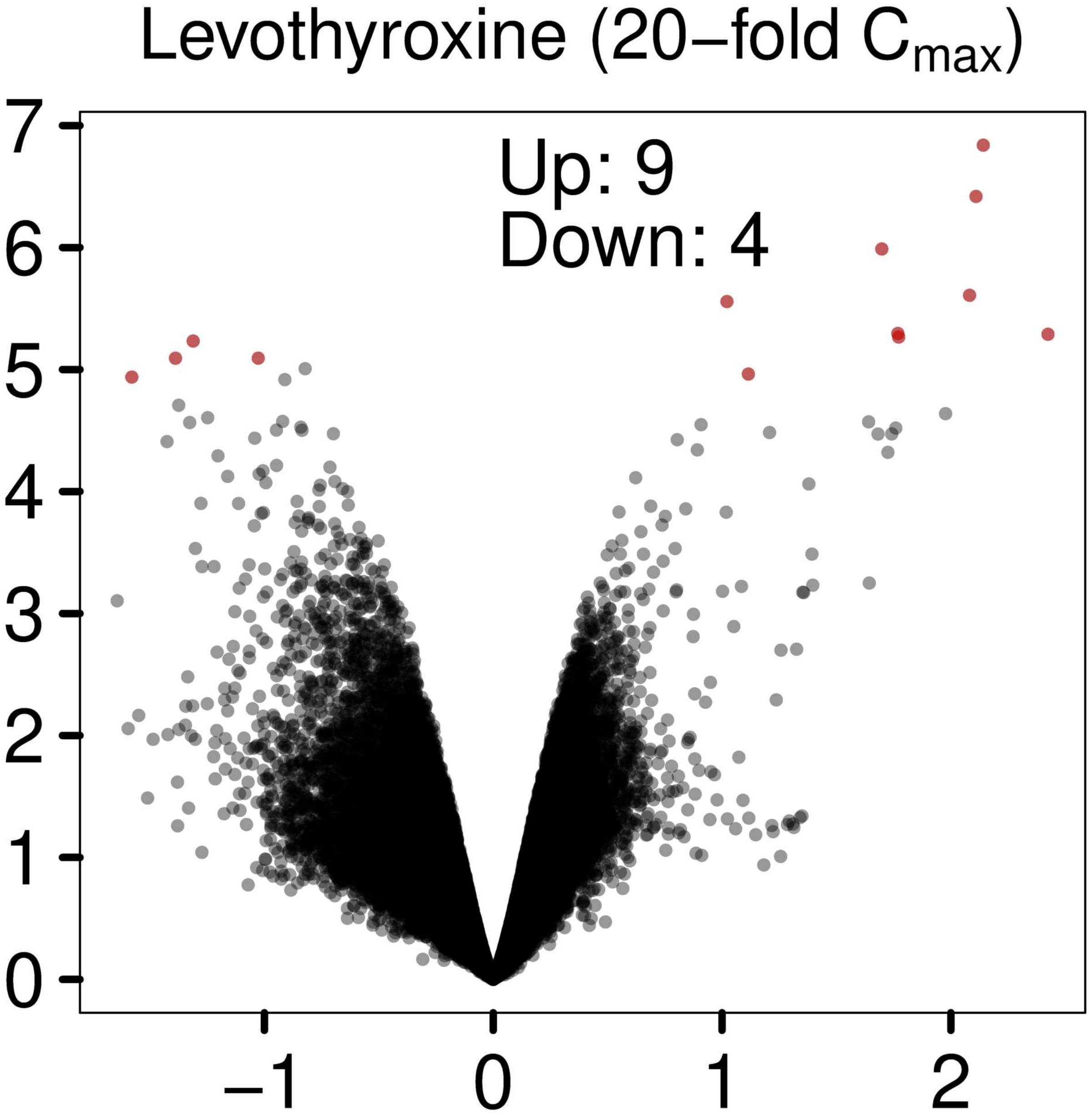

Levothyroxine (20−fold C_{max})
Up: 9
Down: 4
7
6
5
4
3
2
1
0
−1
0
1
2

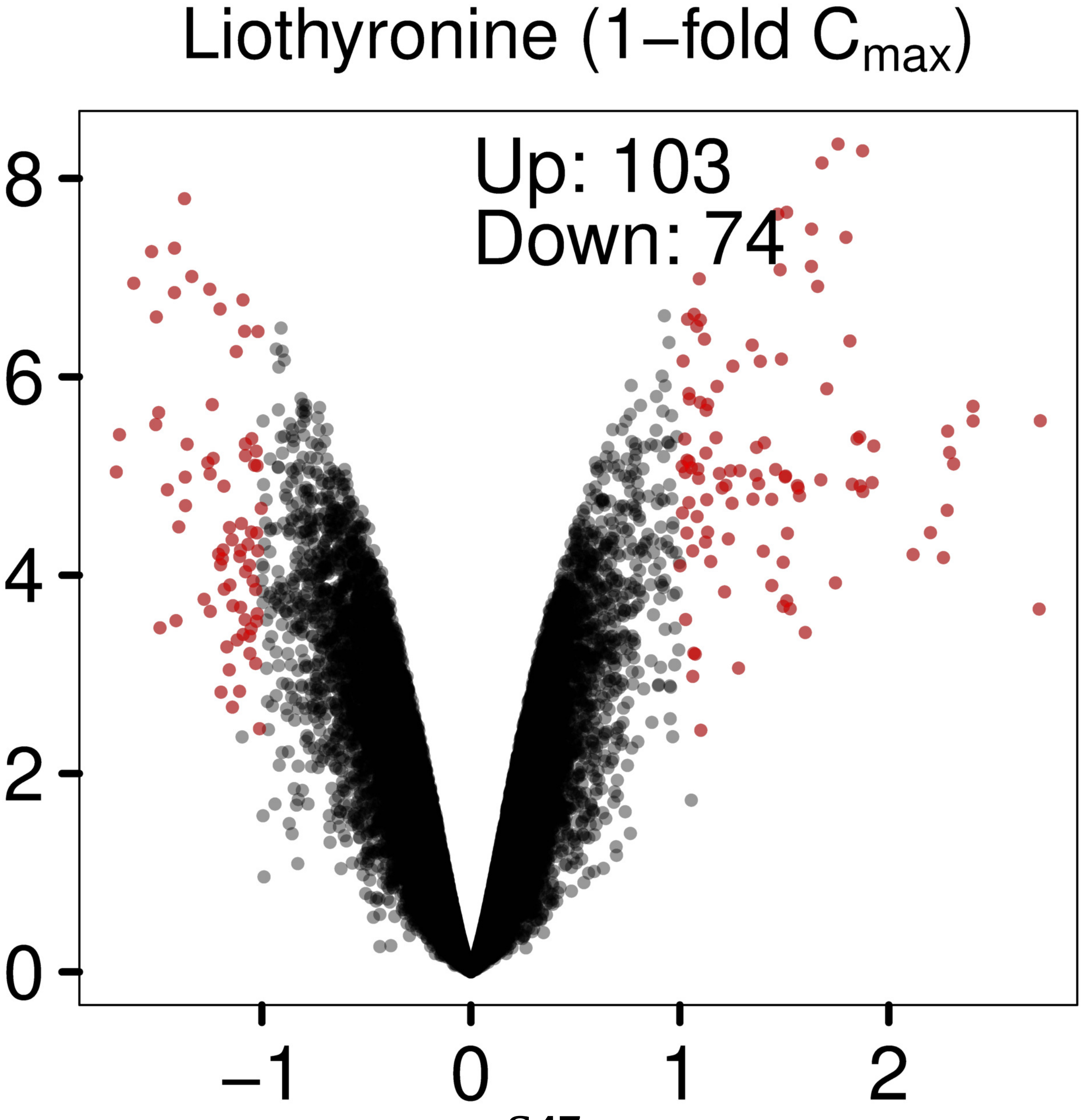
Liothyronine (1−fold C$_{max}$)
Up: 103
Down: 74
8
6
4
2
0
−1
0
1
2

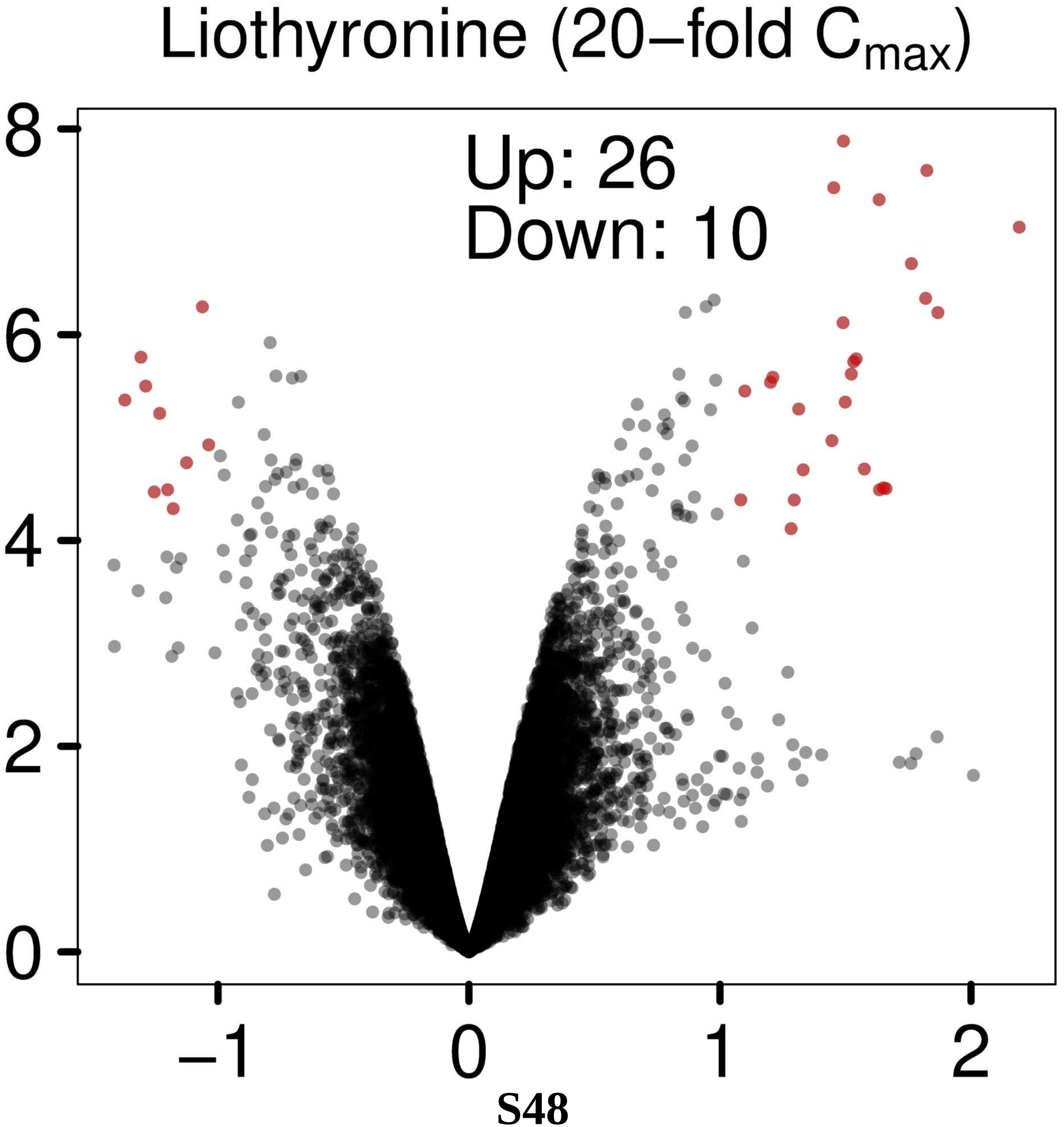

Liothyronine (20–fold C max)
Up: 26
Down: 10
8
6
4
2
0
–1
0
1
2

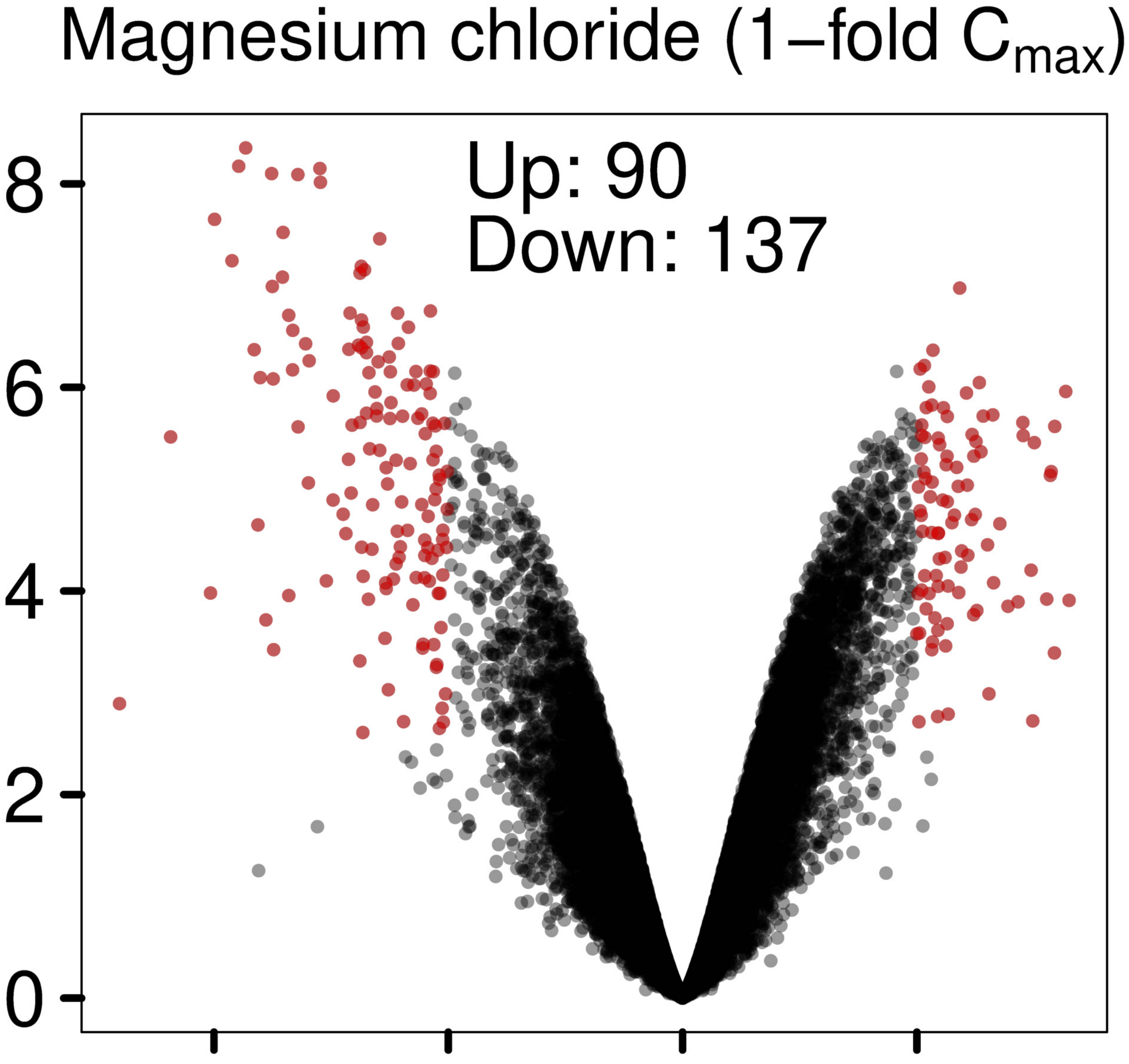

S49

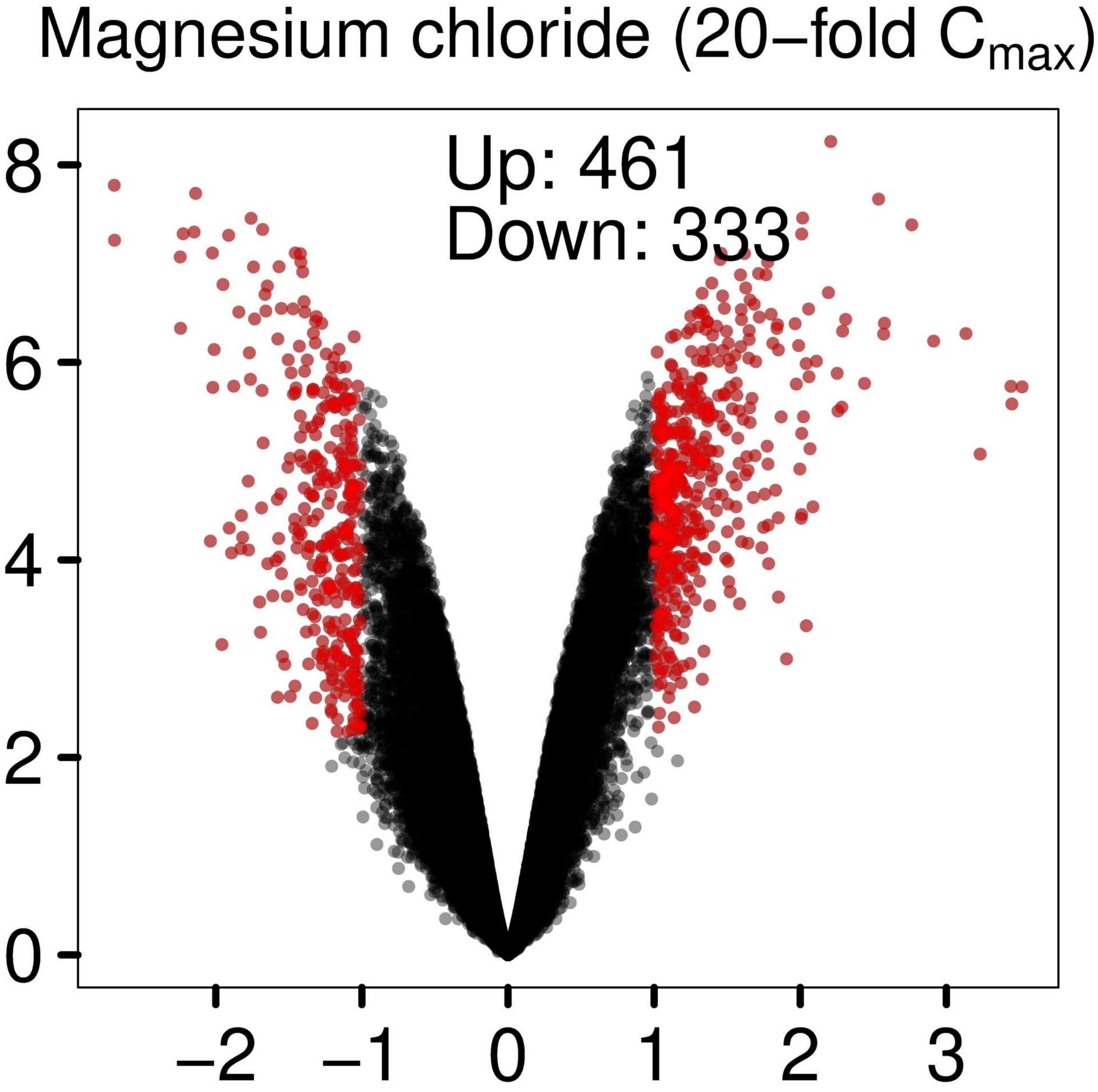
Magnesium chloride (20–fold C_{max})
Up: 461
Down: 333
8
6
4
2
0
–2
–1
0
1
2
3

S50

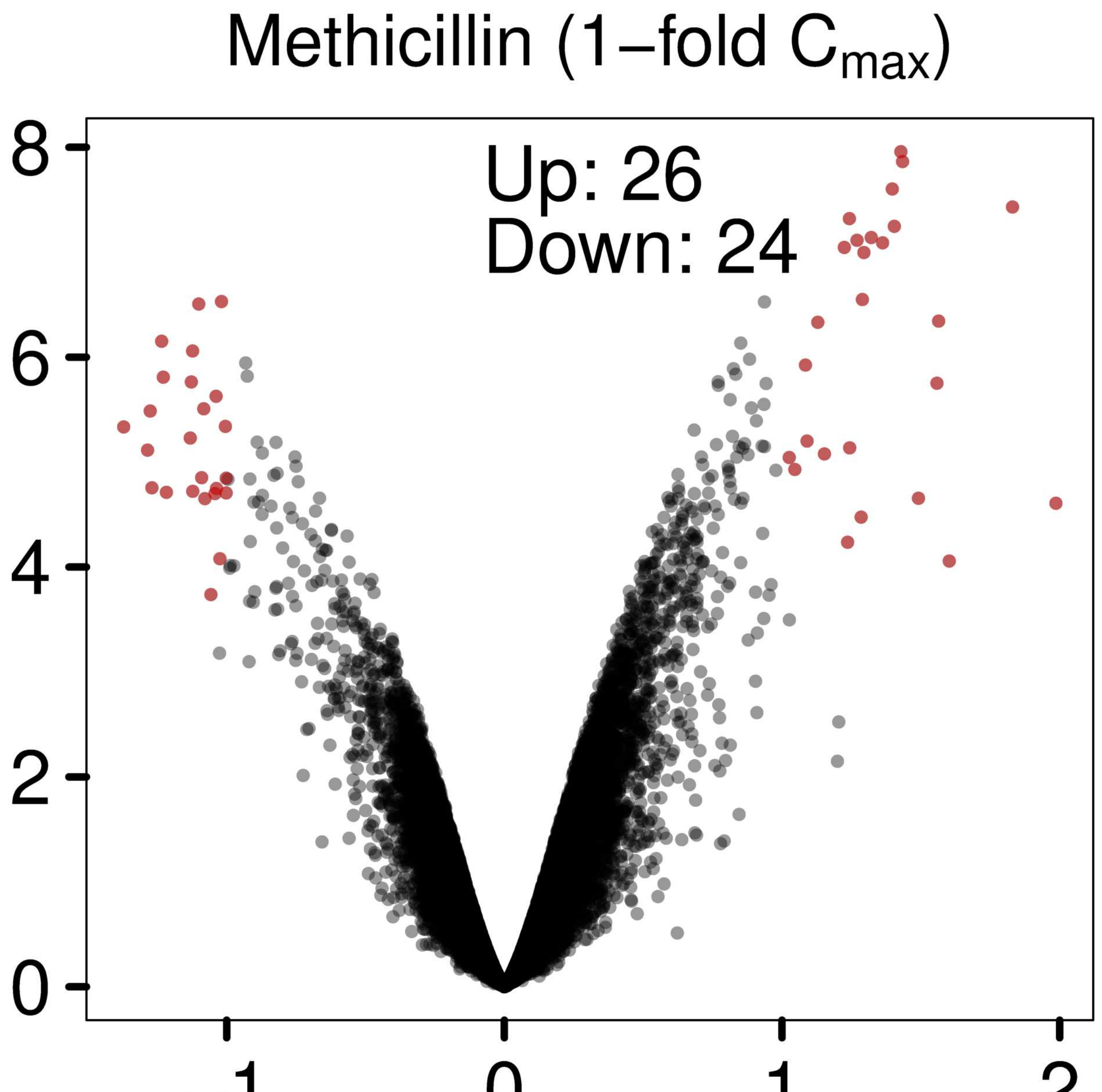
Methicillin (1–fold C max)
Up: 26
Down: 24
8
6
4
2
0
–1
0
1
2

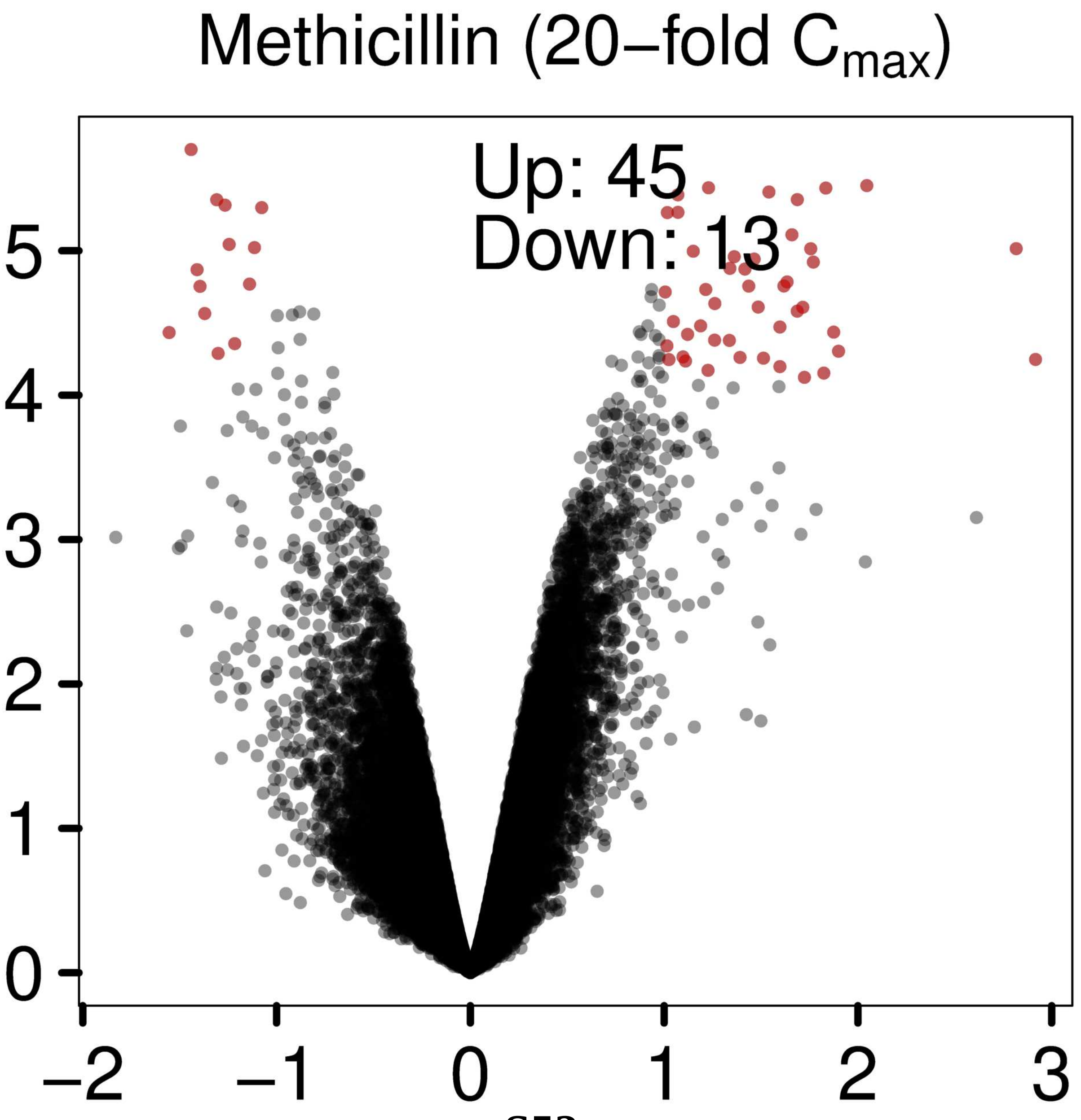
Methicillin (20−fold C_{max})
Up: 45
Down: 13
5
4
3
2
1
0
−2
−1
0
1
2
3

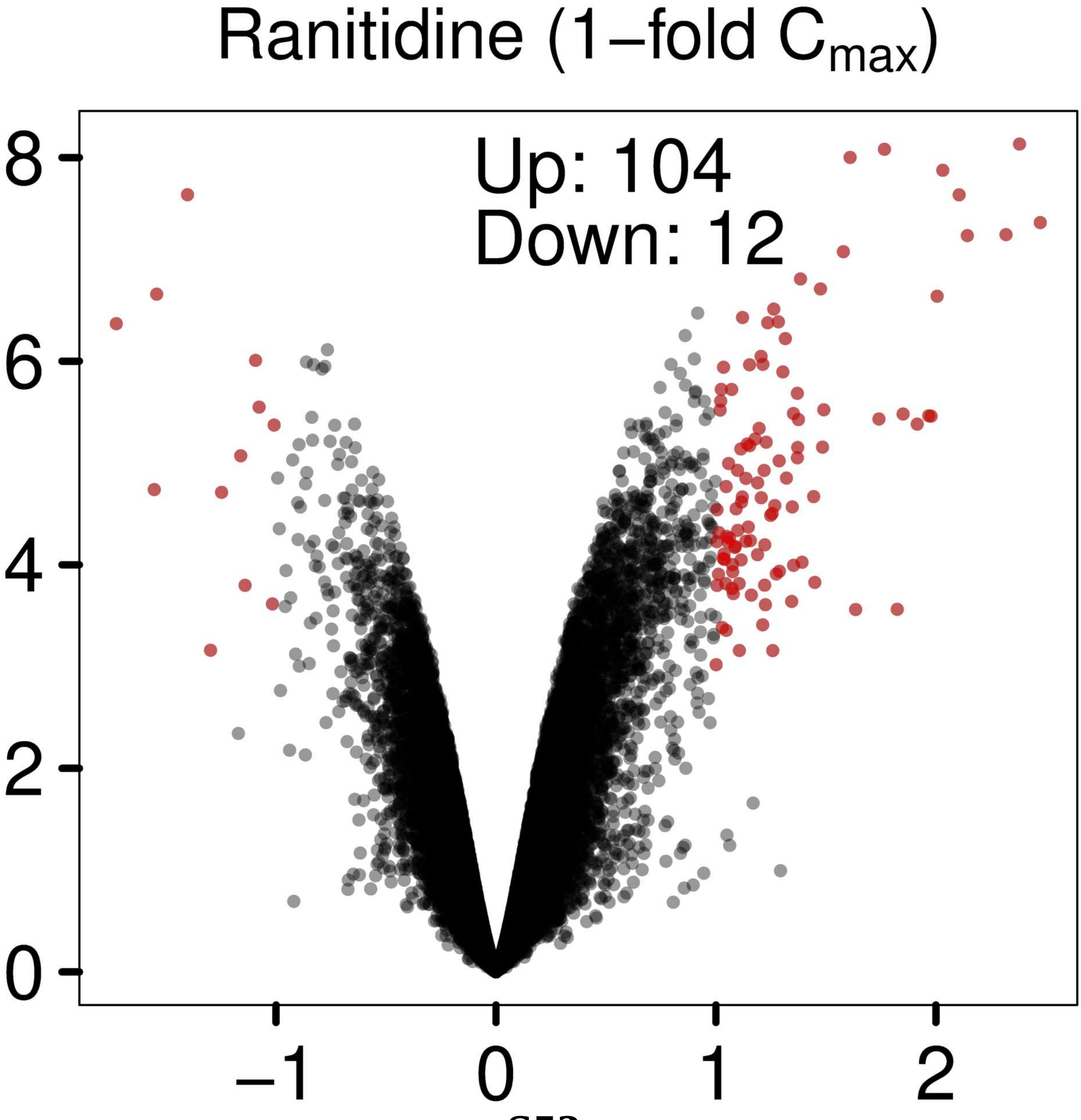
Ranitidine (1–fold C_{max})
Up: 104
Down: 12
8
6
4
2
0
–1
0
1
2

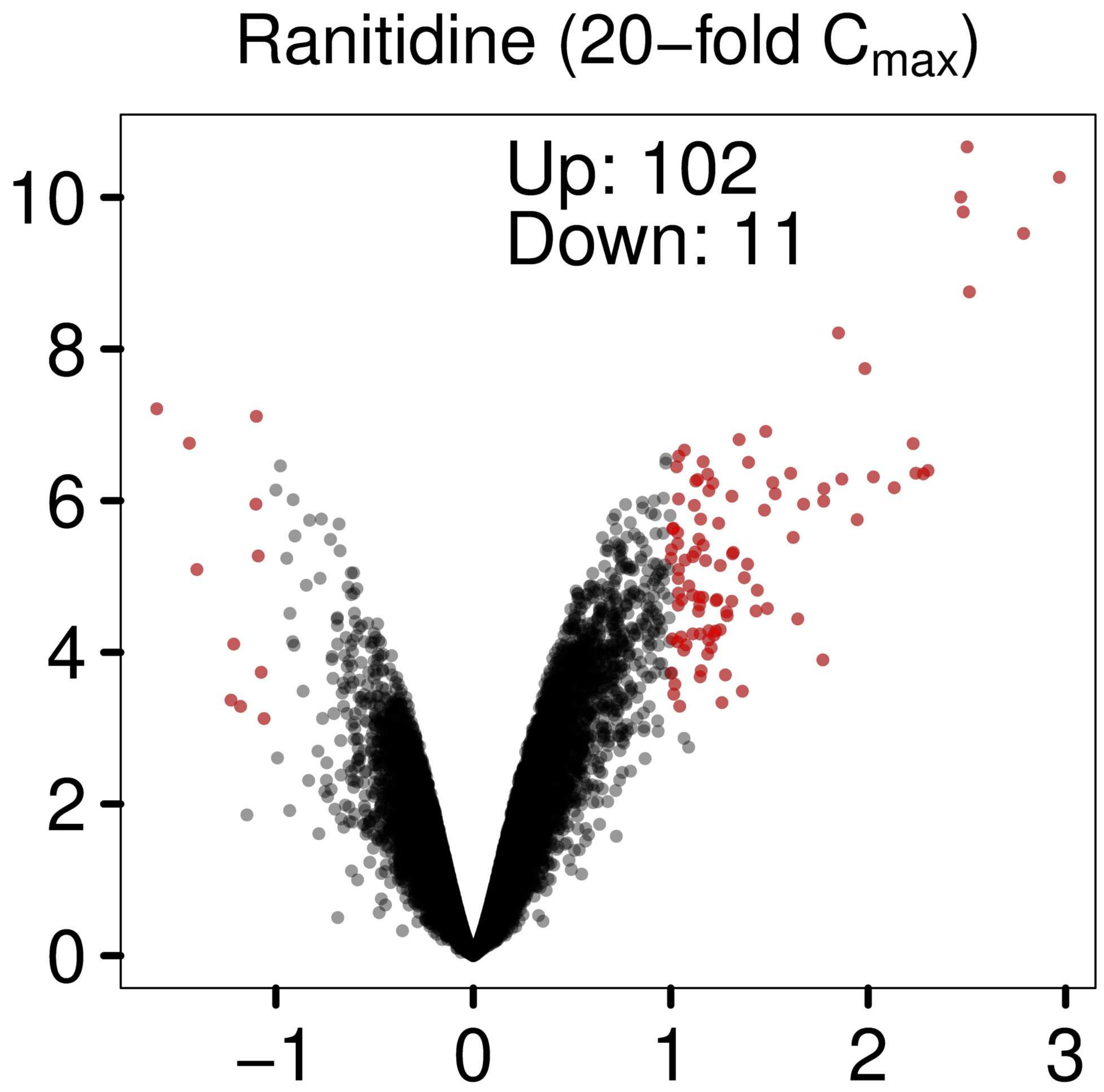
Ranitidine (20−fold C_{max})
Up: 102
Down: 11
0
2
4
6
8
10
−1
0
1
2
3

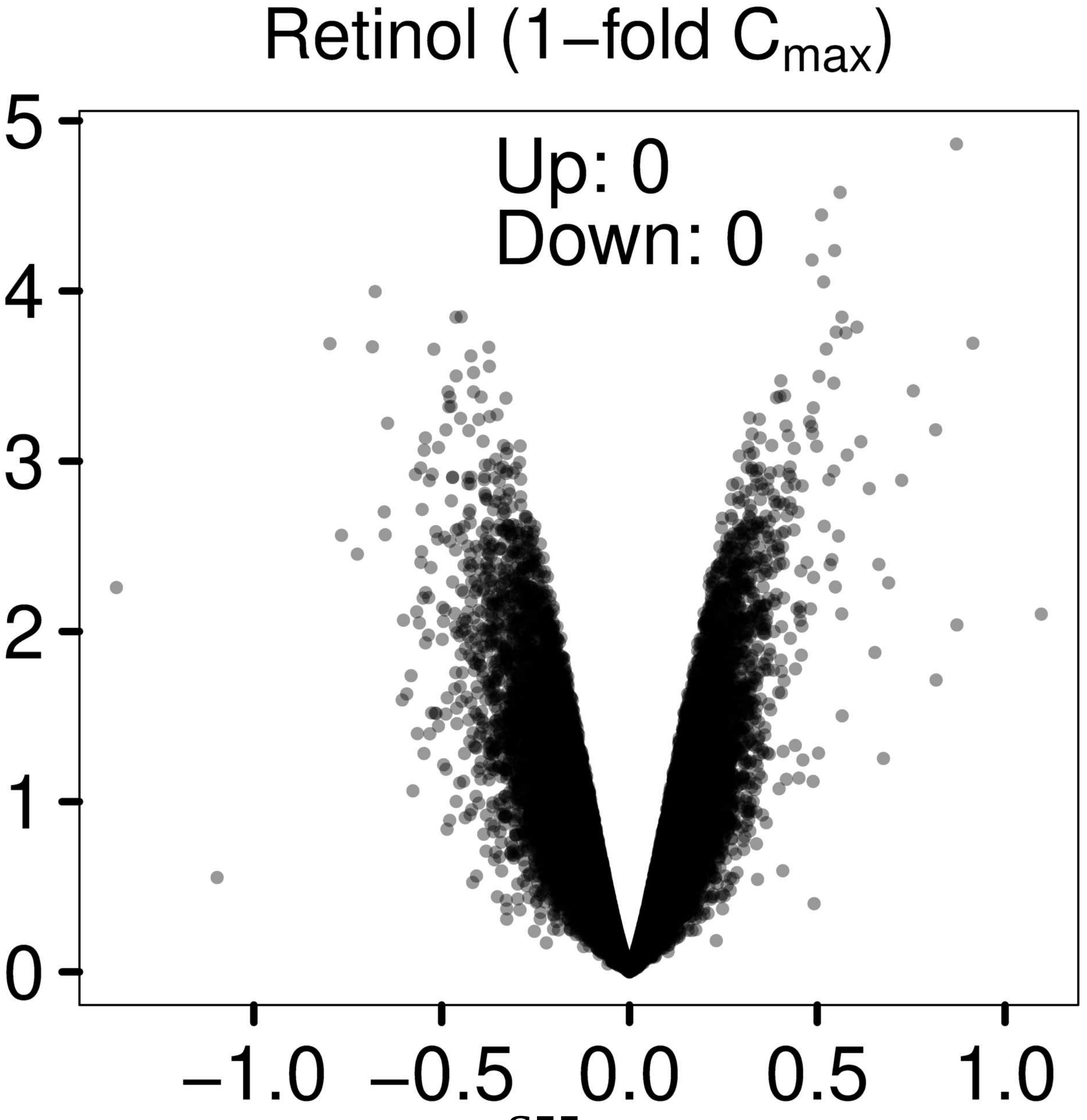
Retinol (1–fold C$_{max}$)
Up: 0
Down: 0
5
4
3
2
1
0
−1.0
−0.5
0.0
0.5
1.0

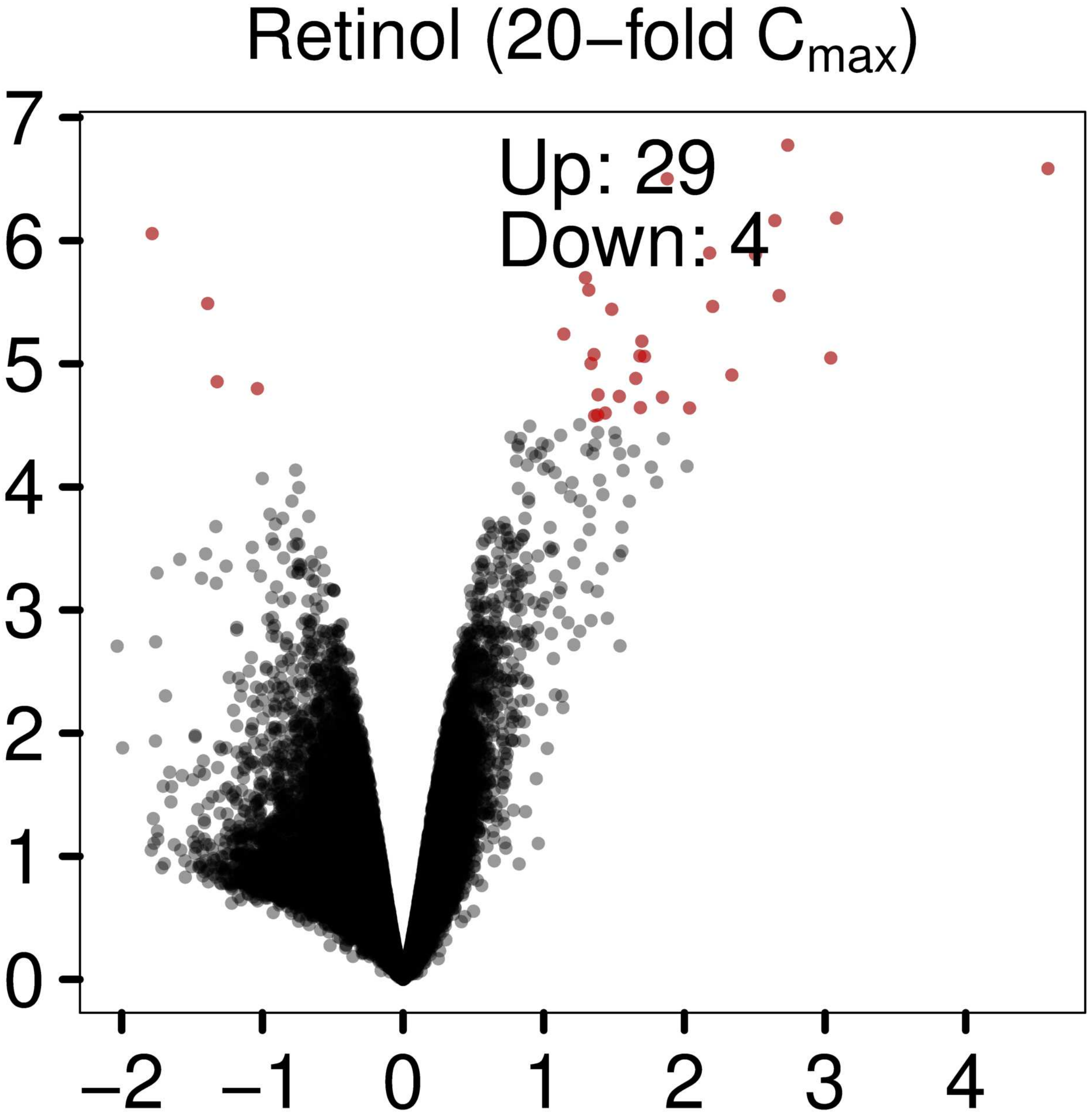
Retinol (20–fold C$_{max}$)
Up: 29
Down: 4
7
6
5
4
3
2
1
0
–2
–1
0
1
2
3
4

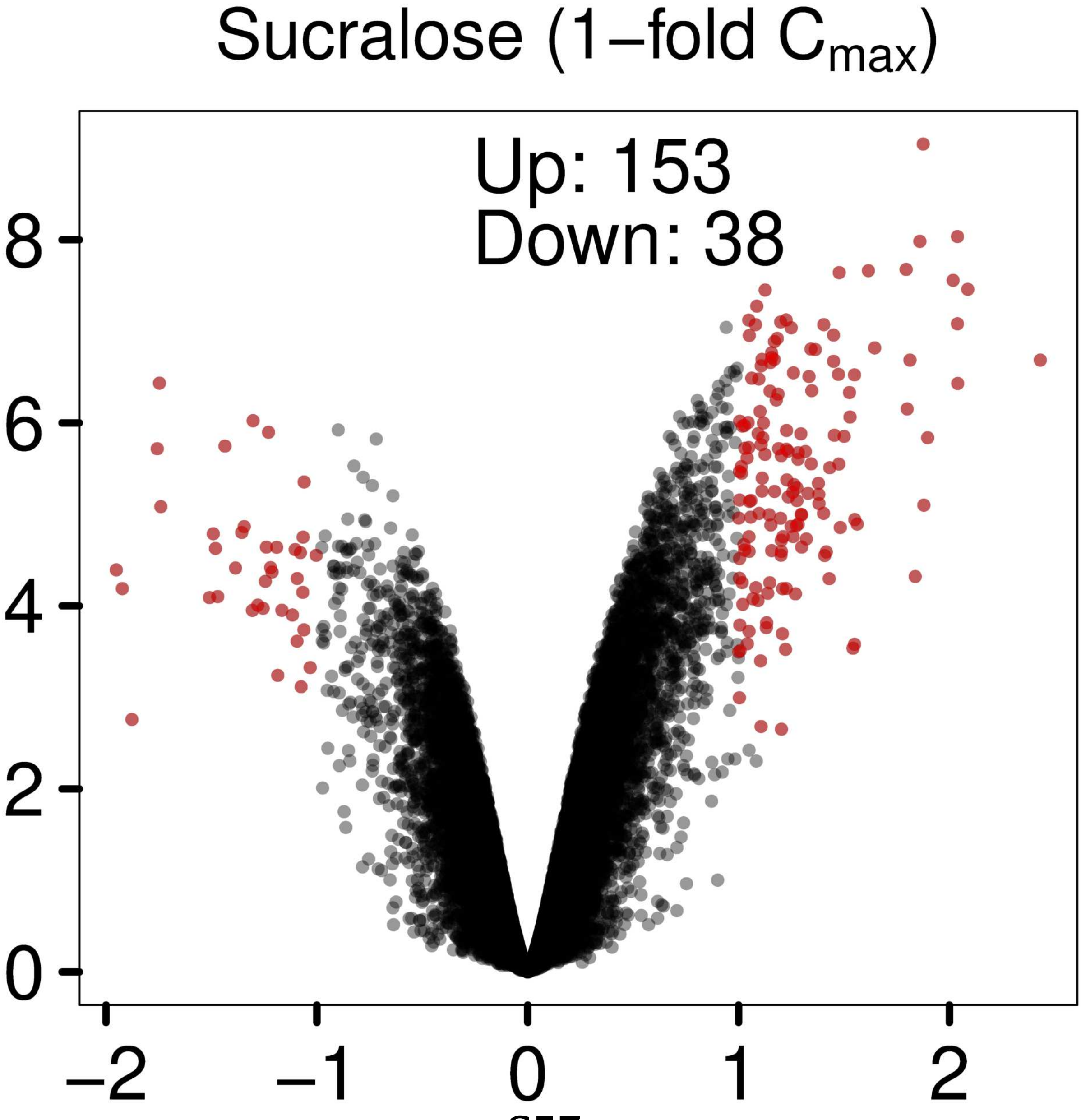
Sucralose (1–fold C_{max})
Up: 153
Down: 38
8
6
4
2
0
–2
–1
0
1
2

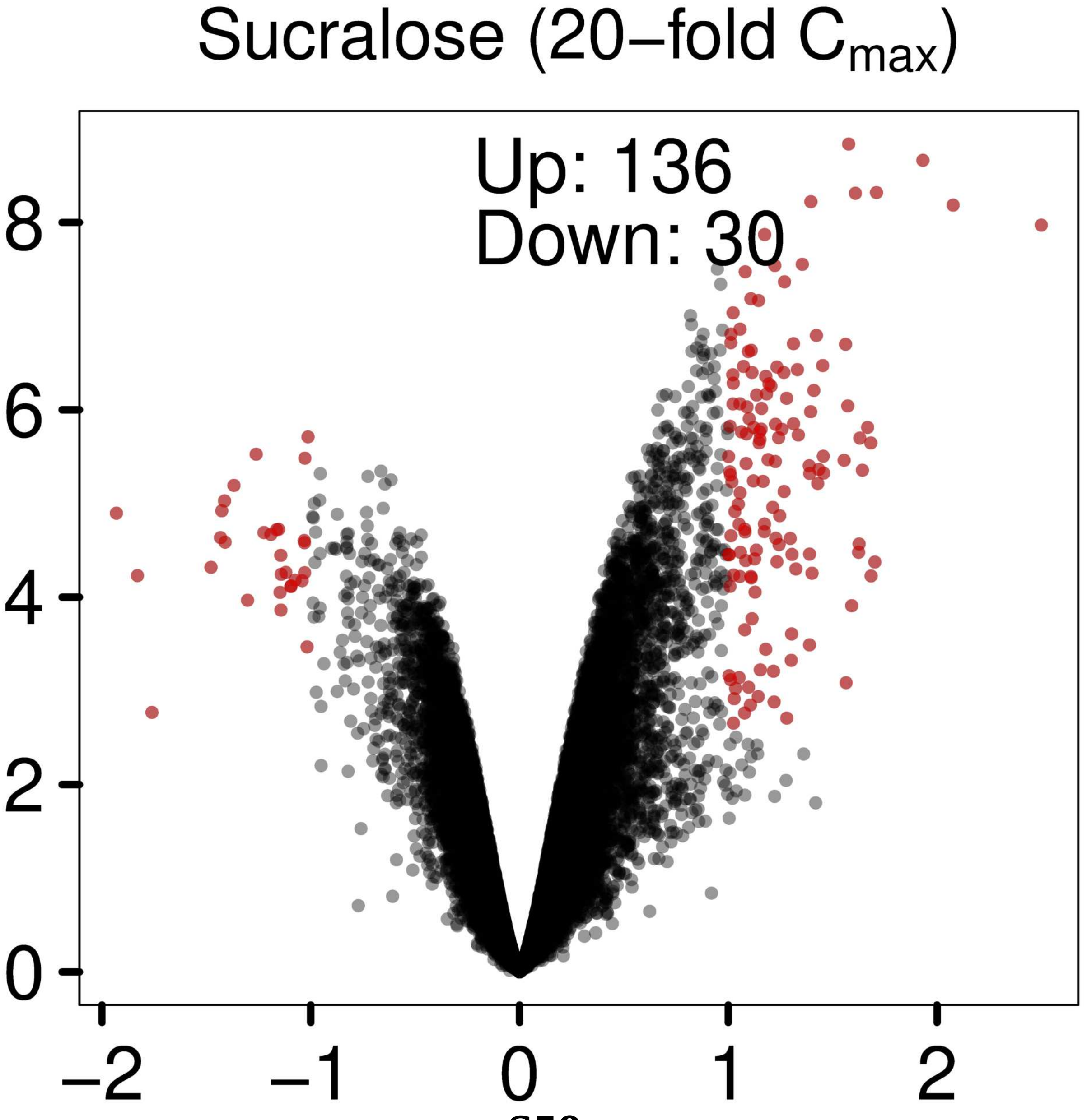
Sucralose (20−fold C_{max})
Up: 136
Down: 30
0
2
4
6
8
−2
−1
0
1
2

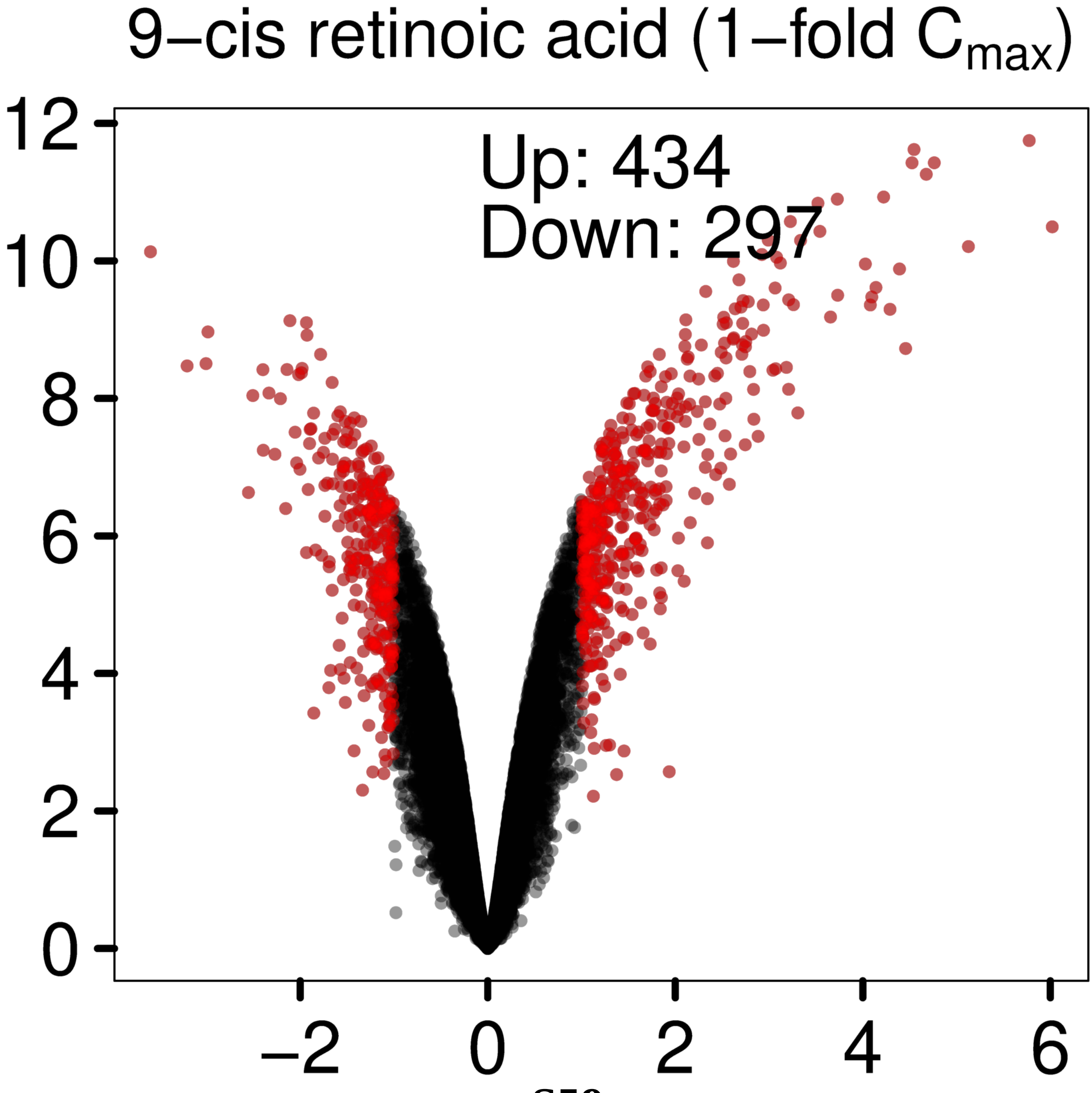
9−cis retinoic acid (1−fold C_{max})
Up: 434
Down: 297
12
10
8
6
4
2
0
−2
0
2
4
6

9−cis retinoic acid (20−fold C_{max})

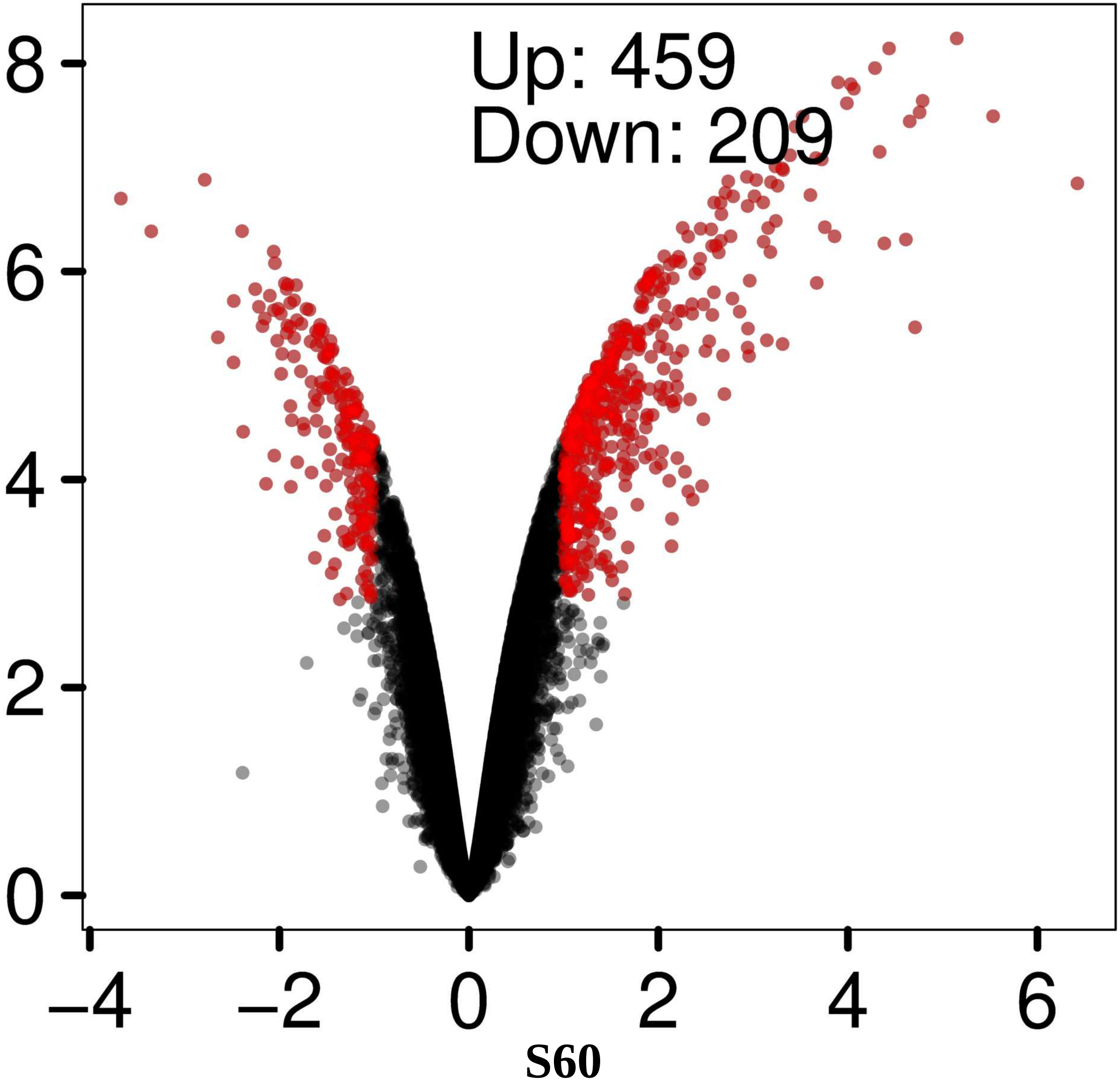

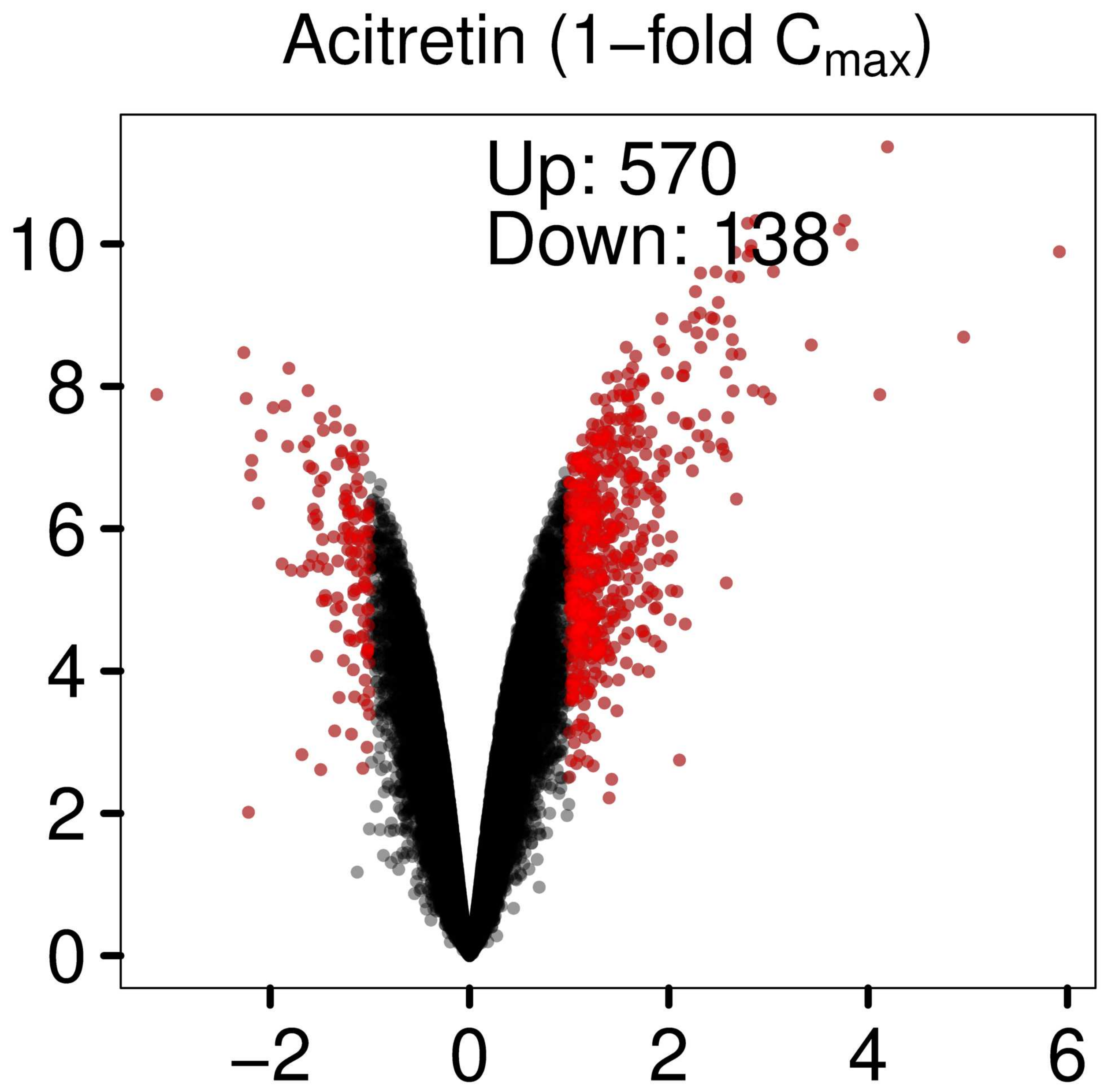

Acitretin (1−fold C_{max})
Up: 570
Down: 138
10
8
6
4
2
0
−2
0
2
4
6

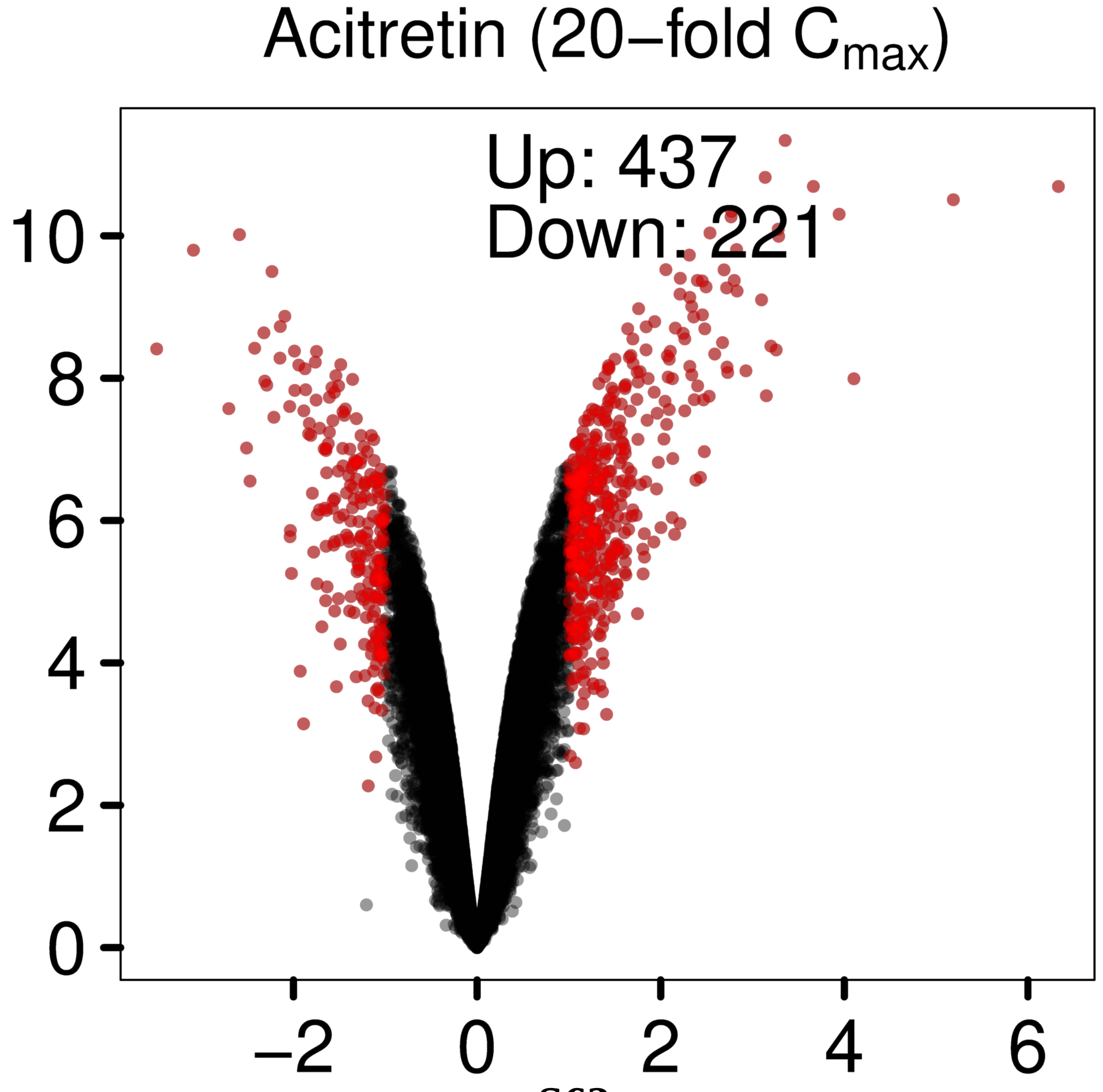
Acitretin (20–fold C_{max})
Up: 437
Down: 221
10
8
6
4
2
0
−2
0
2
4
6

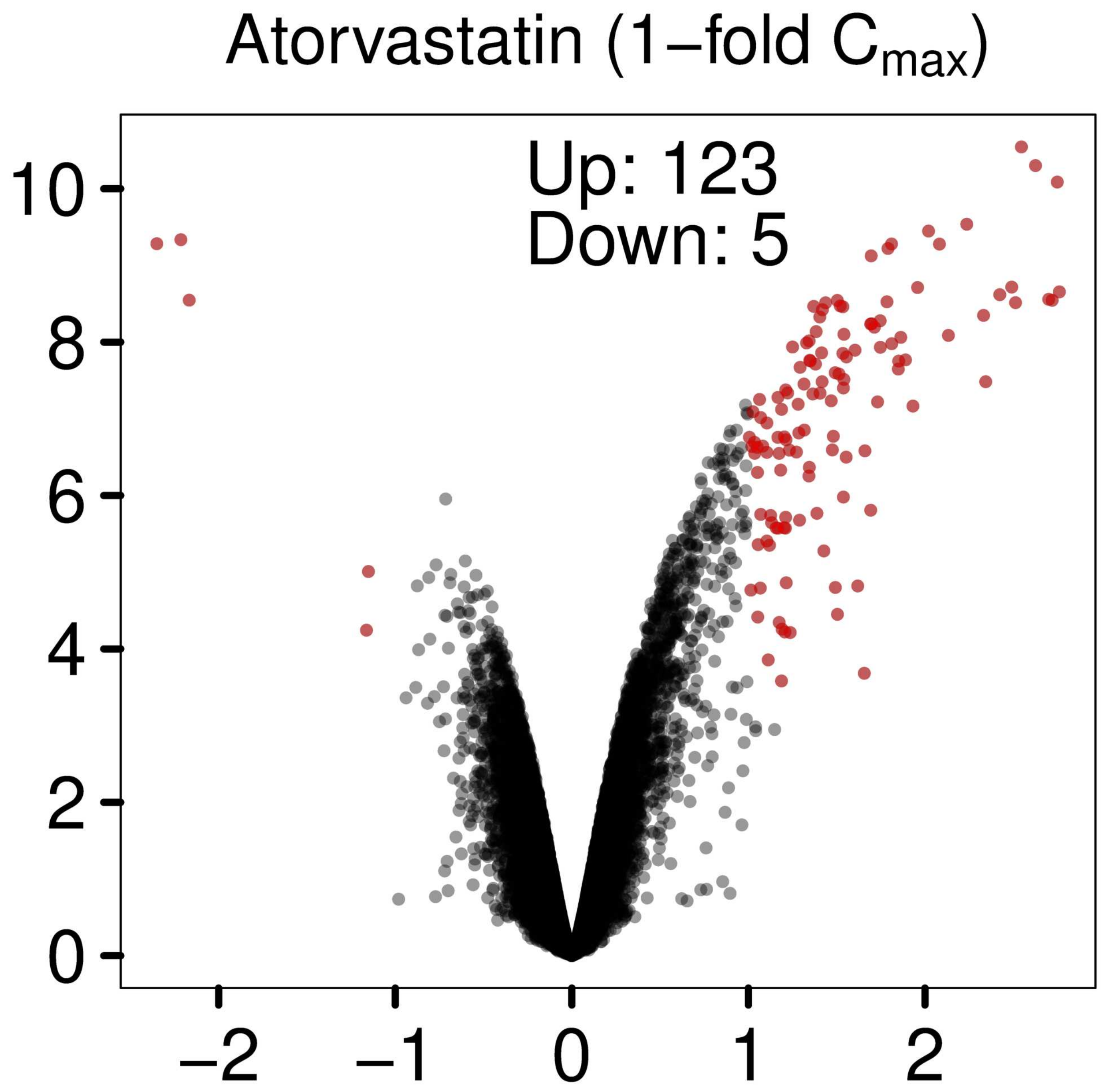
Atorvastatin (1–fold C_{max})
Up: 123
Down: 5
10
8
6
4
2
0
−2
−1
0
1
2

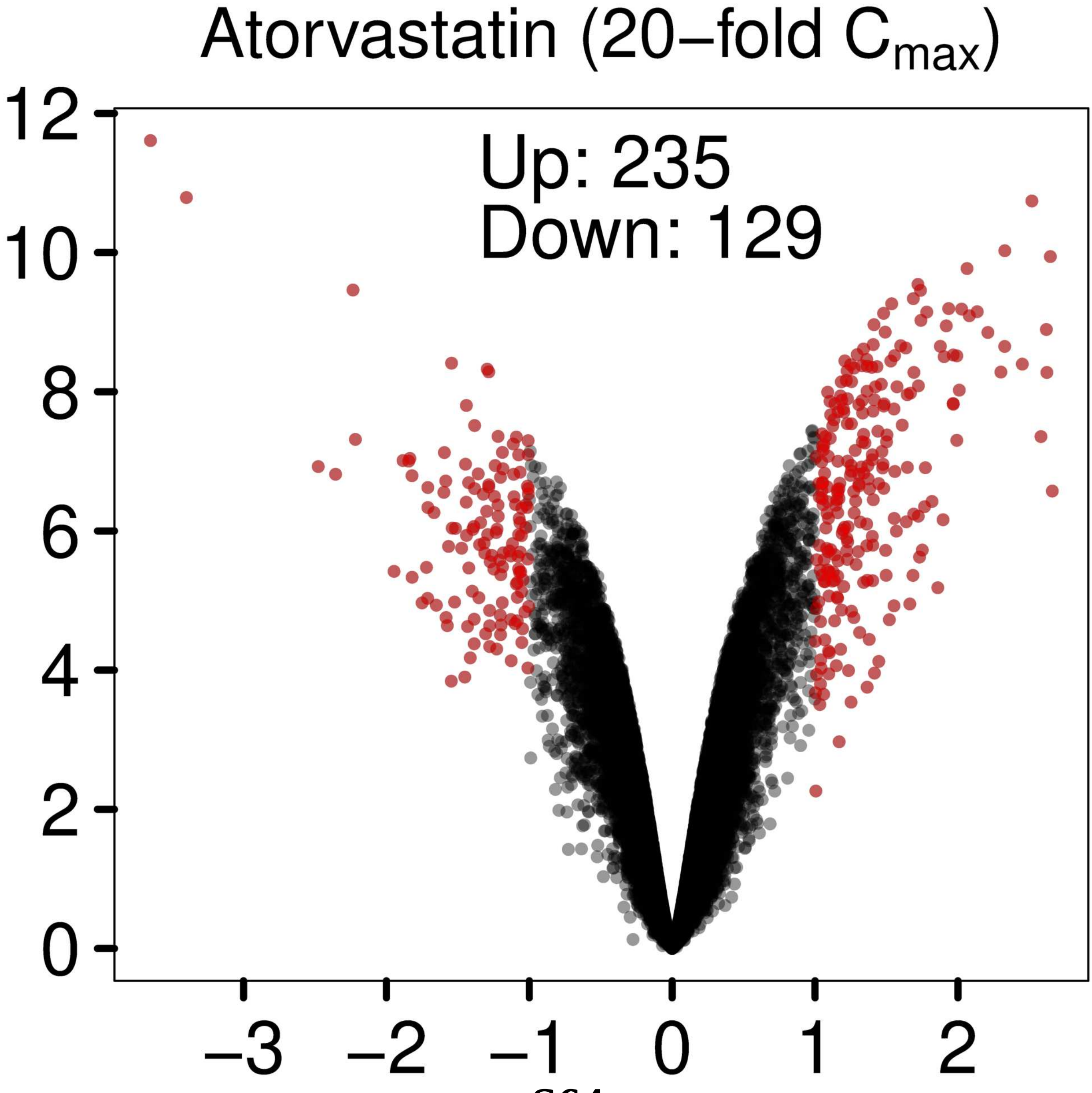
Atorvastatin (20−fold C_{max})
Up: 235
Down: 129
12
10
8
6
4
2
0
−3
−2
−1
0
1
2

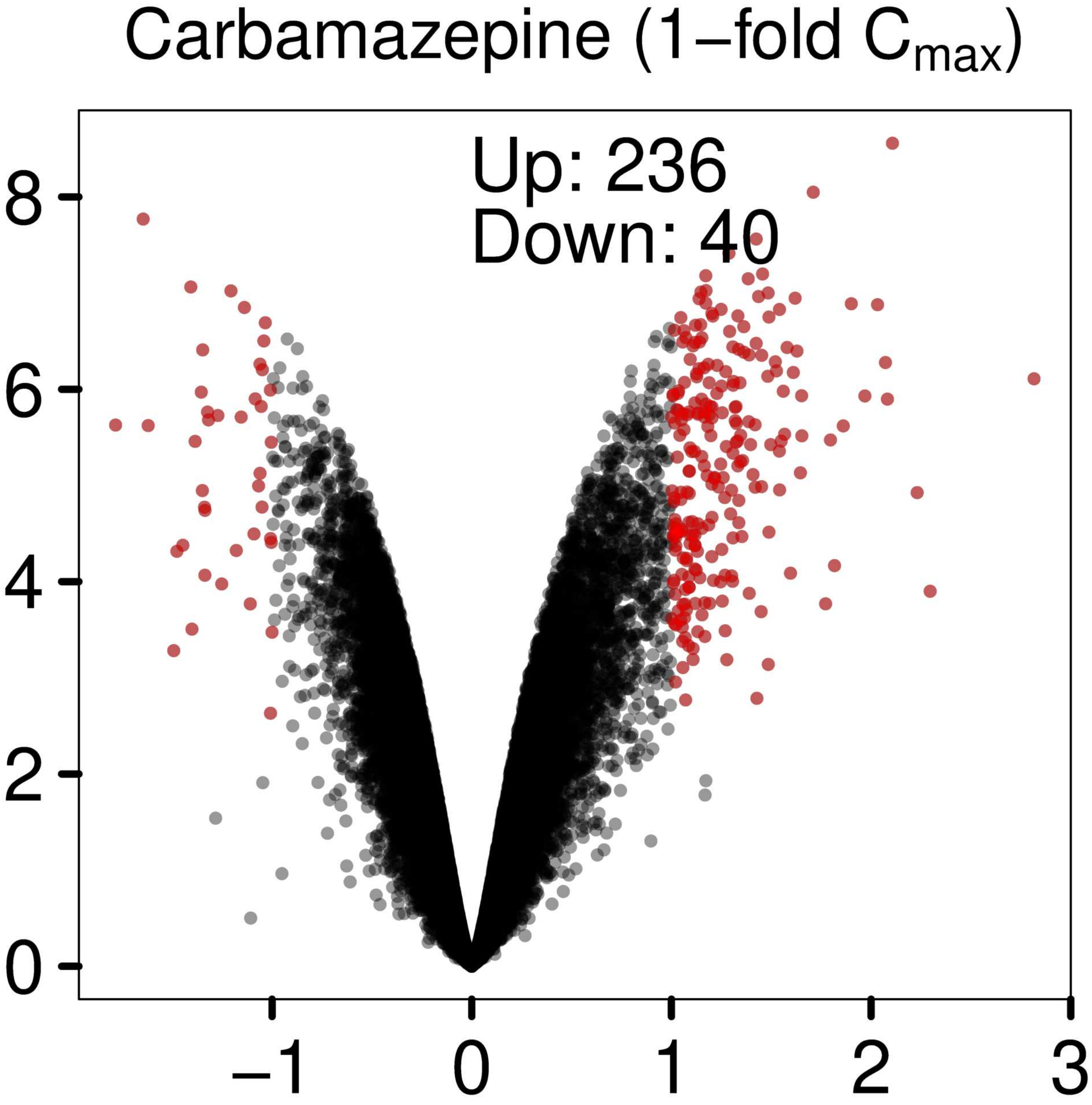
Carbamazepine (1−fold C_{max})
Up: 236
Down: 40
8
6
4
2
0
−1
0
1
2
3

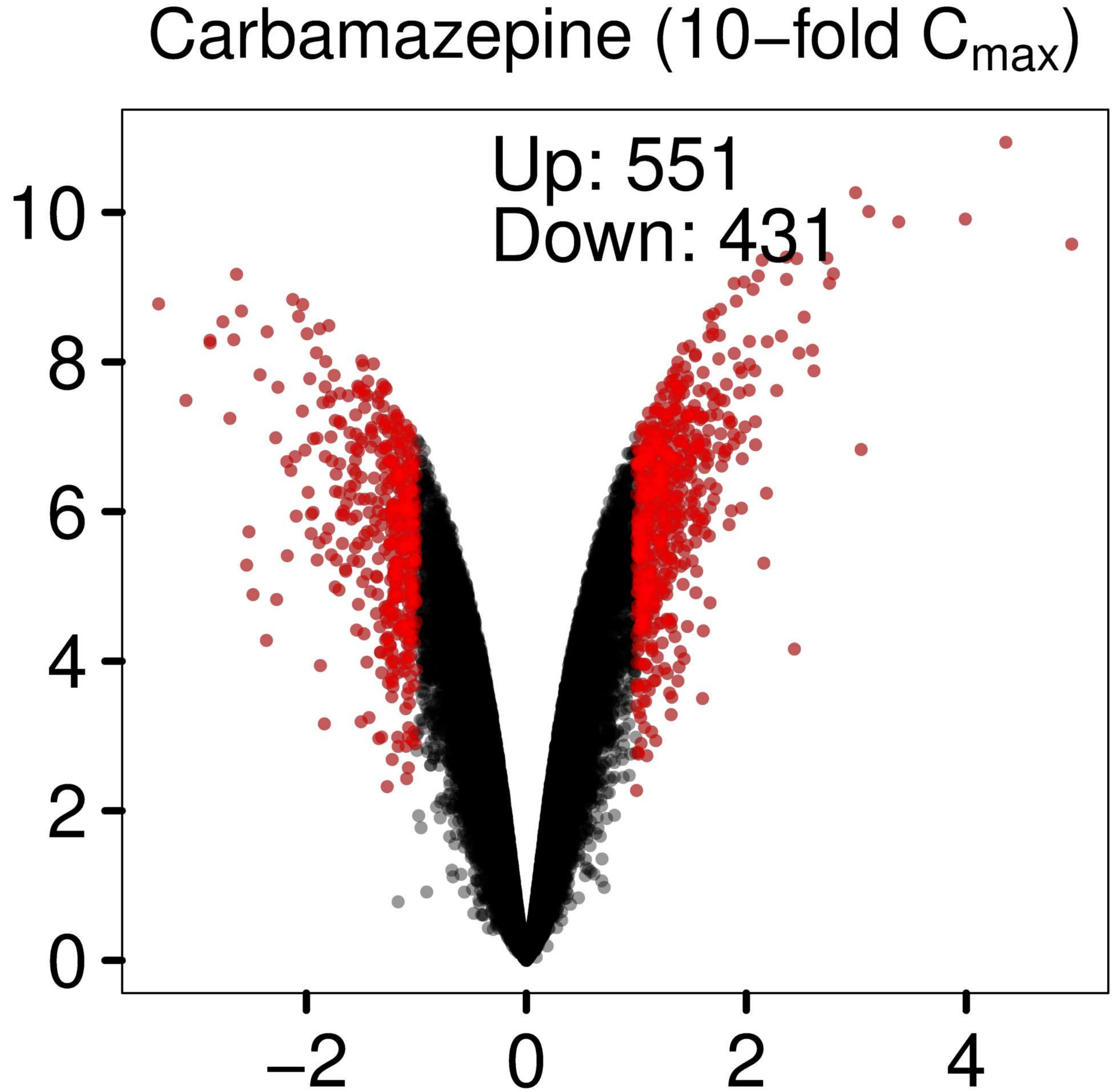
Carbamazepine (10–fold C_{max})
Up: 551
Down: 431
10
8
6
4
2
0
–2
0
2
4

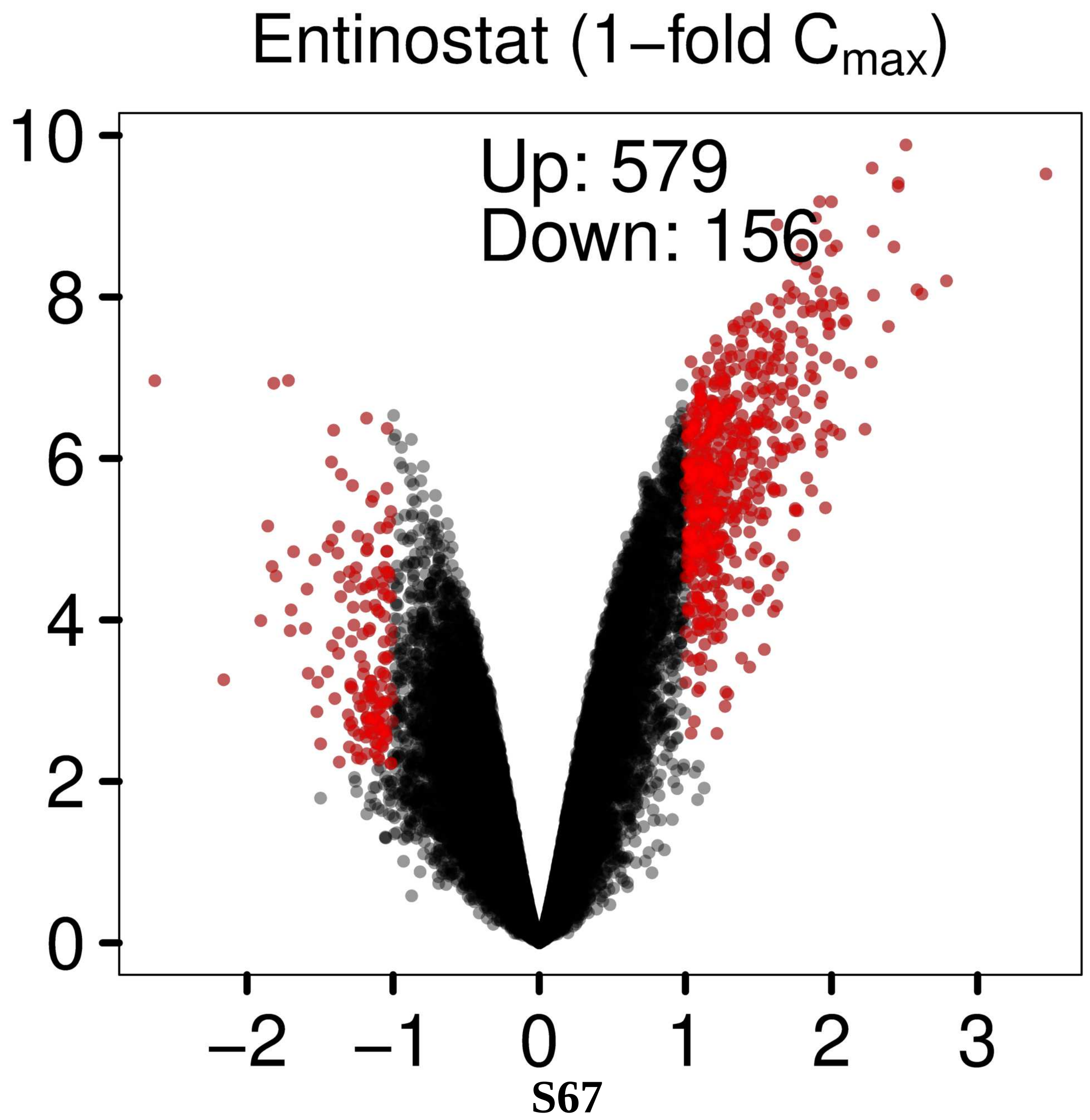

Entinostat (1–fold C_{max})
Up: 579
Down: 156
10
8
6
4
2
0
–2
–1
0
1
2
3

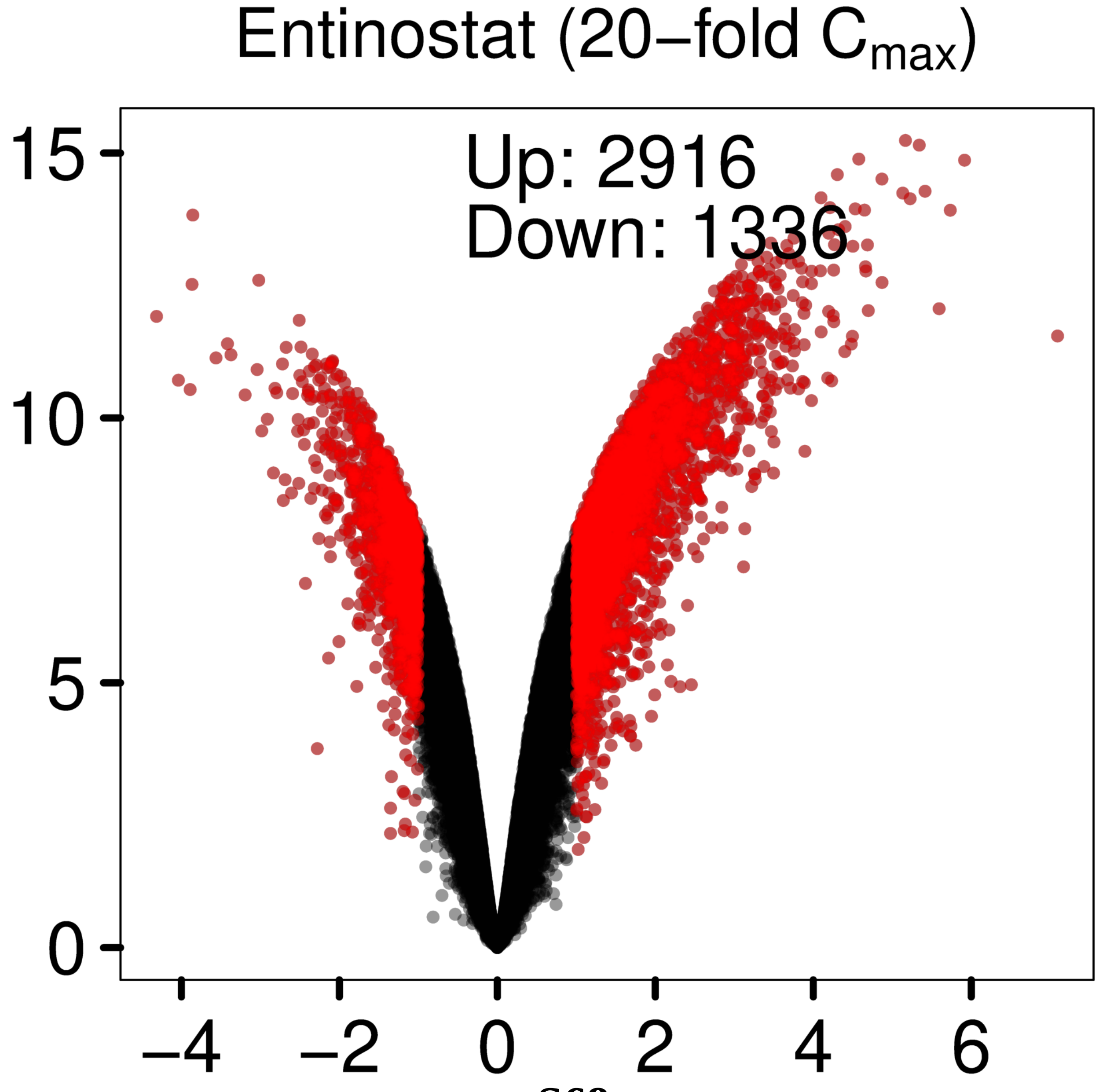
Entinostat (20−fold C$_{max}$)
Up: 2916
Down: 1336
15
10
5
0
−4
−2
0
2
4
6

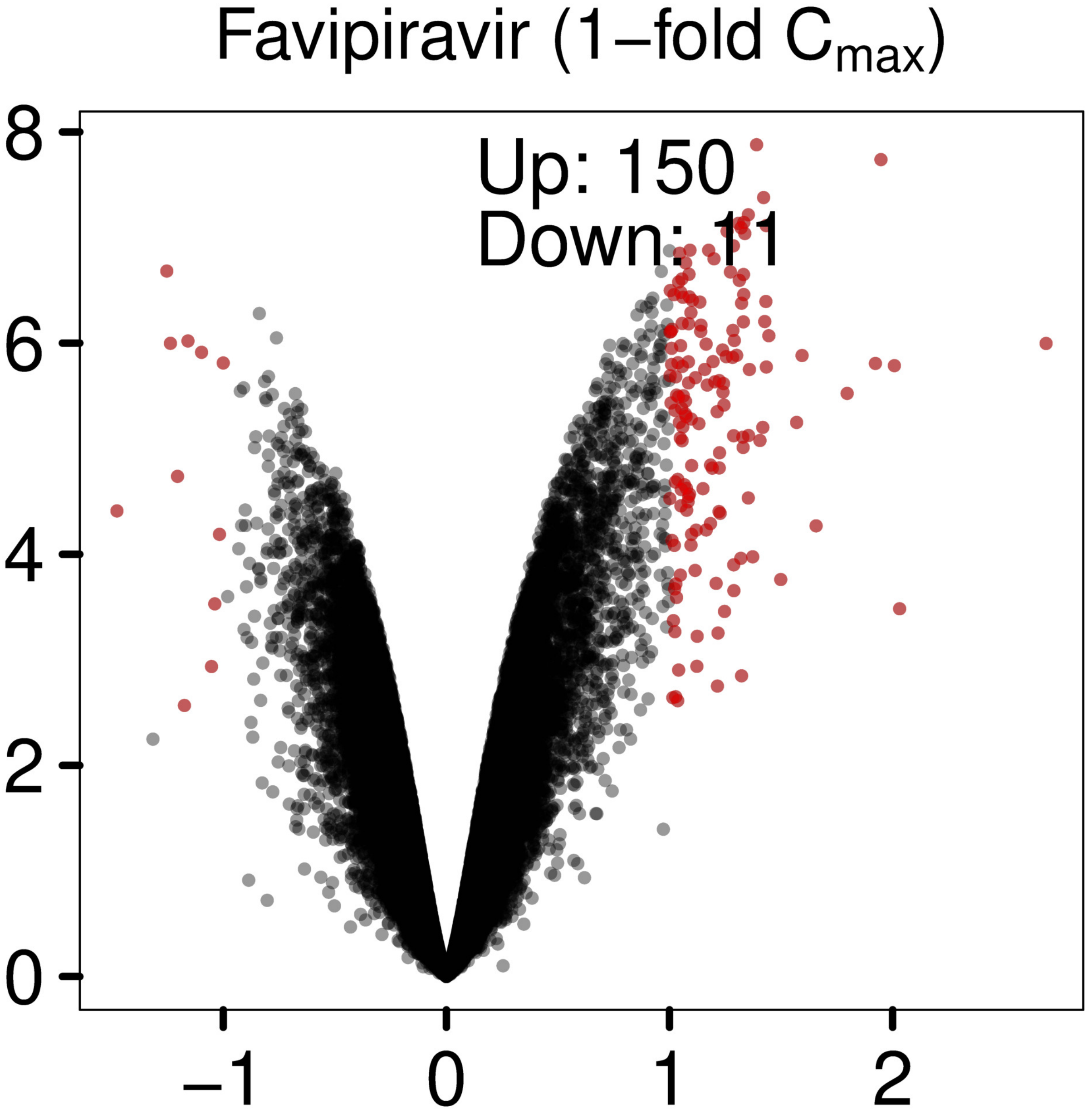
Favipiravir (1–fold C_{max})
Up: 150
Down: 11
8
6
4
2
0
–1
0
1
2

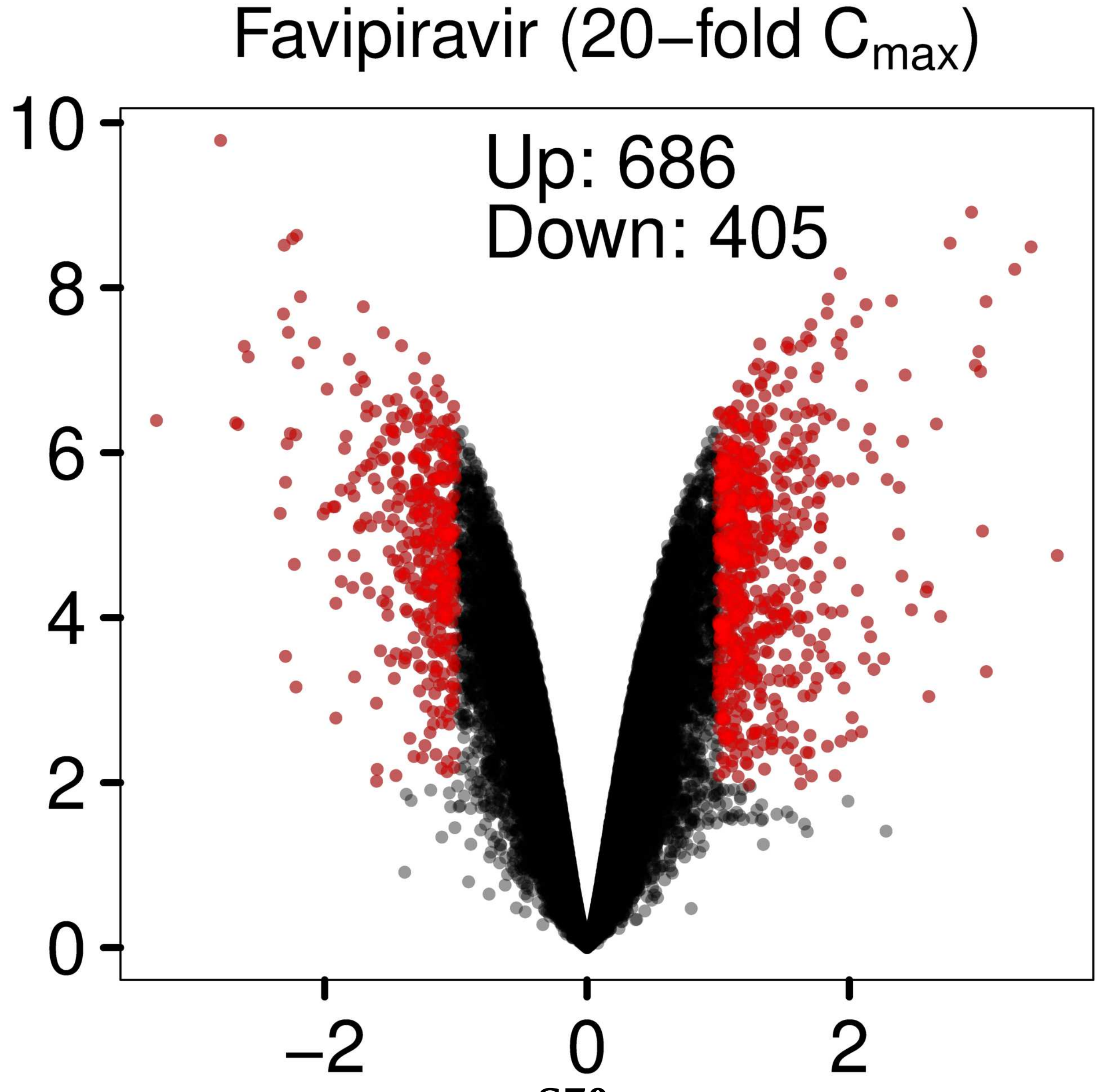
Favipiravir (20−fold C max)
Up: 686
Down: 405
10
8
6
4
2
0
−2
0
2

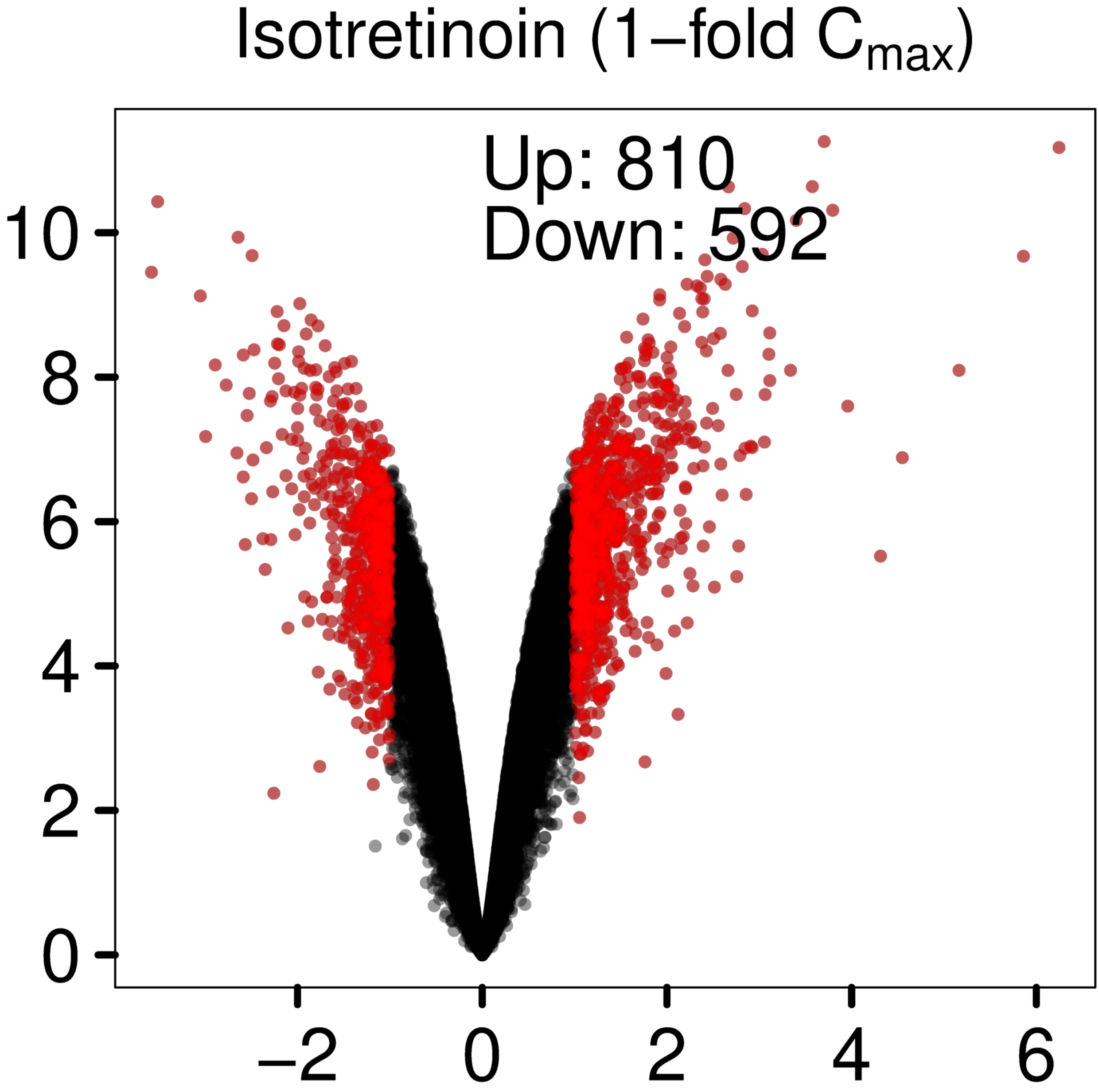
Isotretinoin (1–fold C$_{max}$)
Up: 810
Down: 592
10
8
6
4
2
0
–2
0
2
4
6

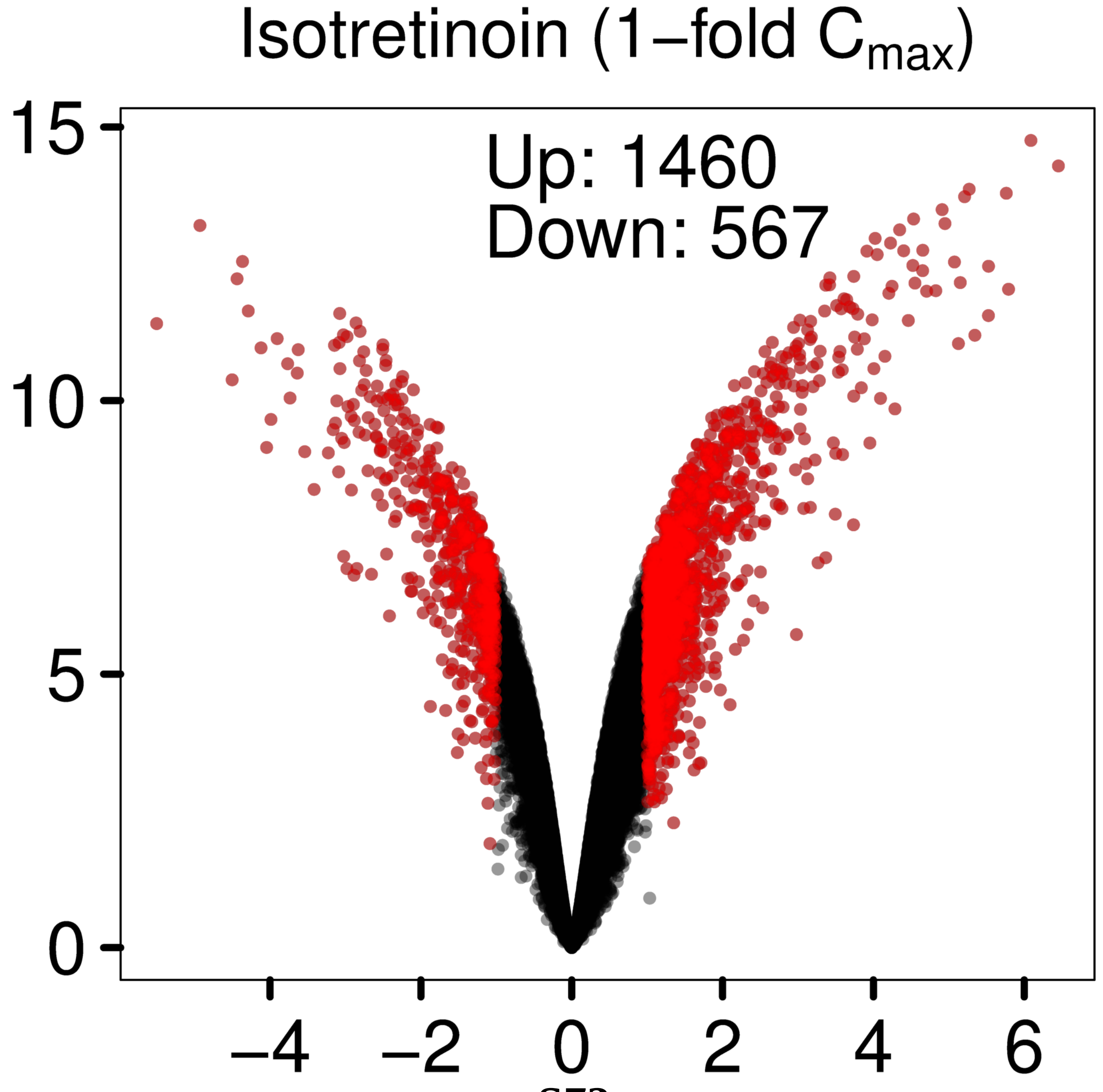
Isotretinoin (1–fold C$_{max}$)
Up: 1460
Down: 567
15
10
5
0
−4
−2
0
2
4
6

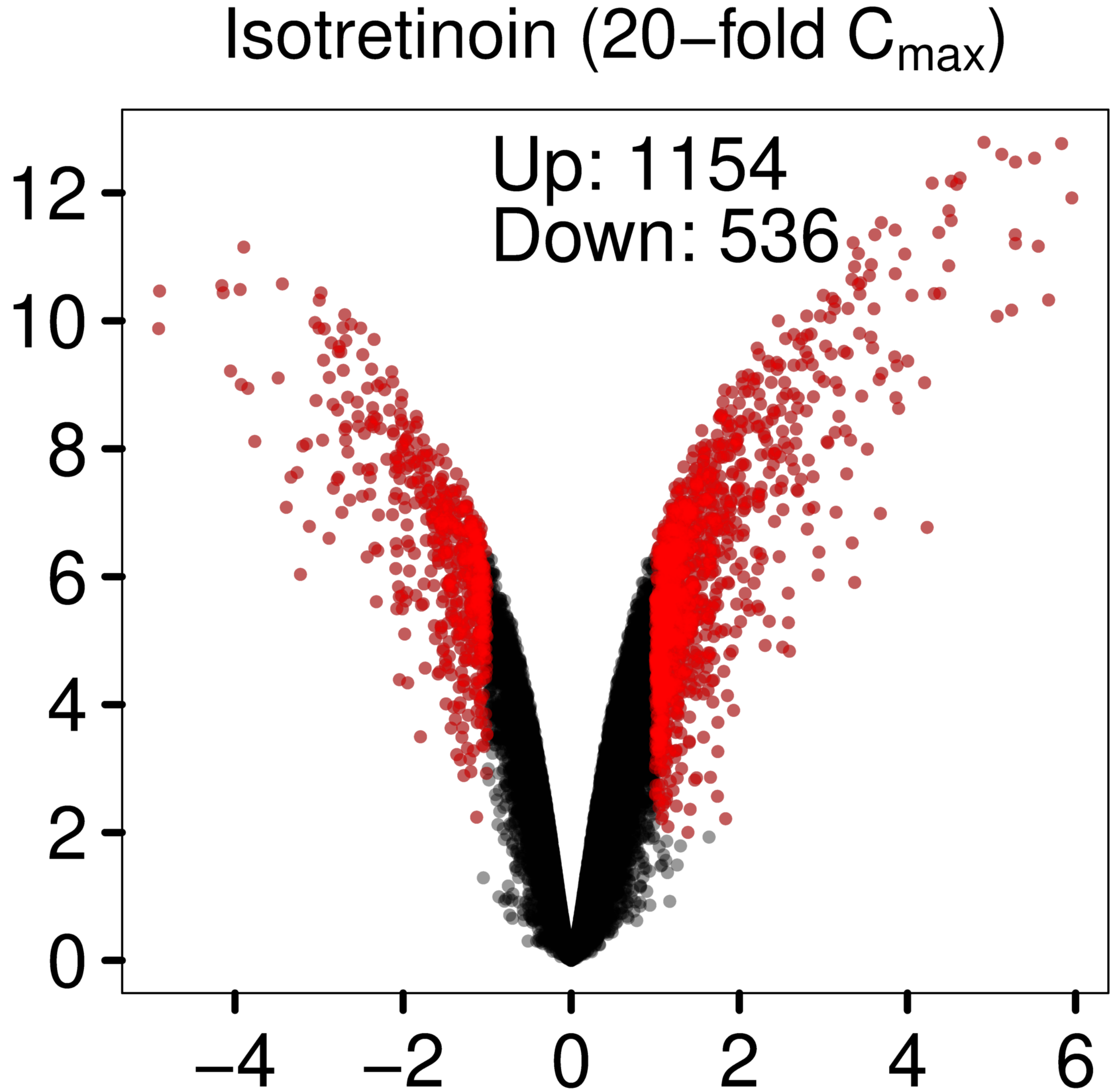
Isotretinoin (20–fold C$_{max}$)
Up: 1154
Down: 536
12
10
8
6
4
2
0
–4
–2
0
2
4
6

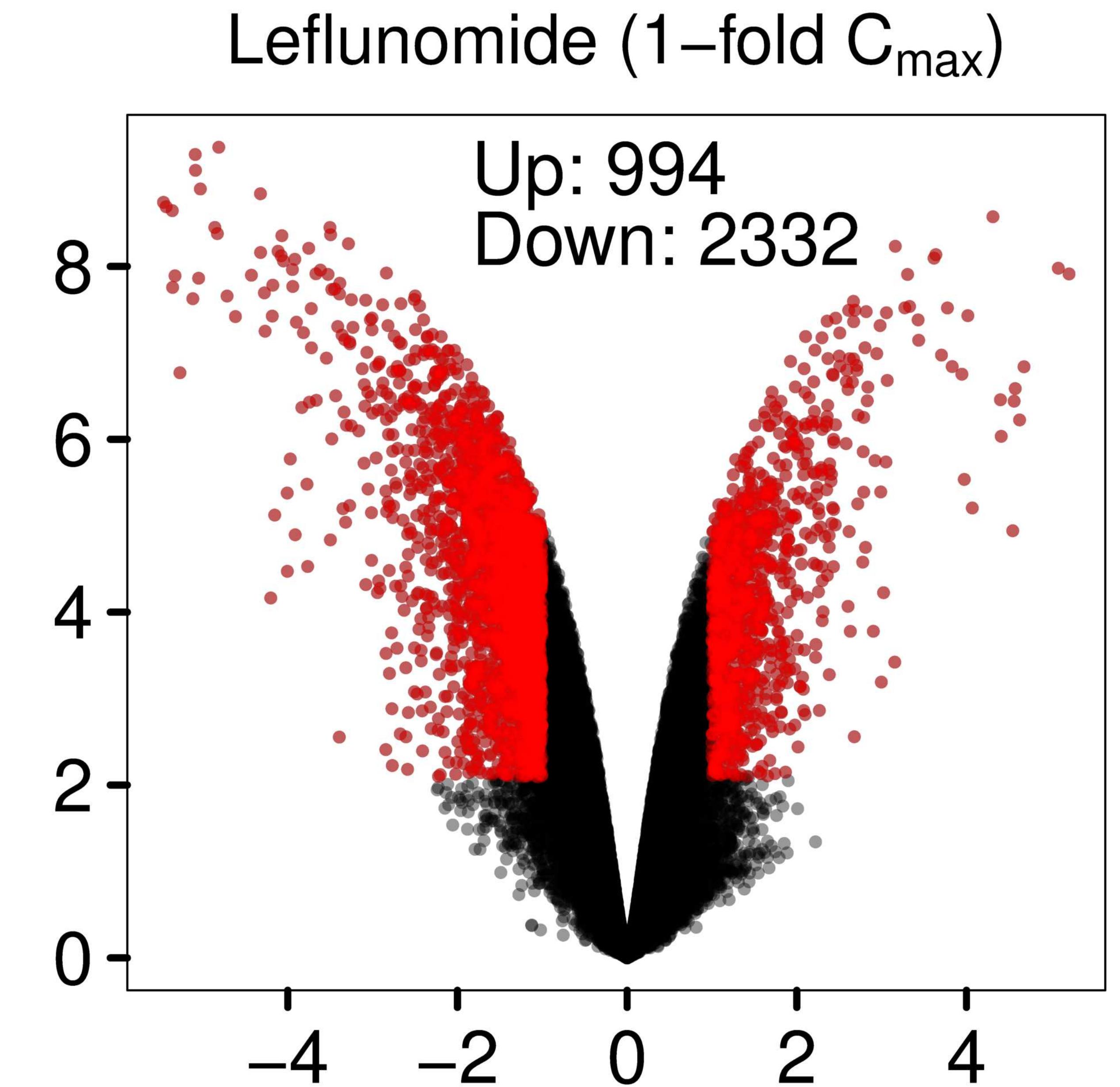
Leflunomide (1−fold C_{max})
Up: 994
Down: 2332
8
6
4
2
0
−4
−2
0
2
4

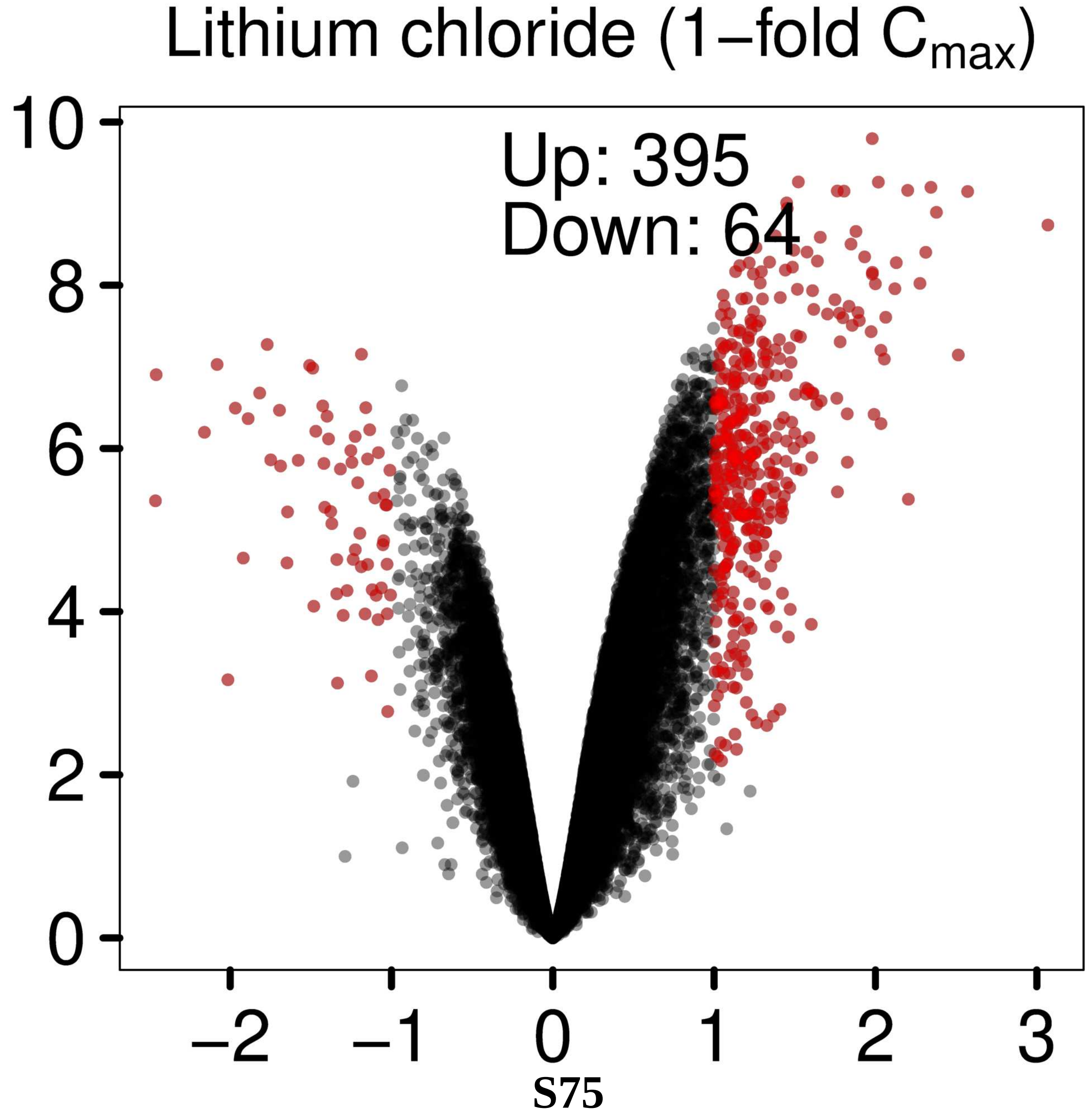

Lithium chloride (1−fold C$_{max}$)
Up: 395
Down: 64
10
8
6
4
2
0
−2
−1
0
1
2
3

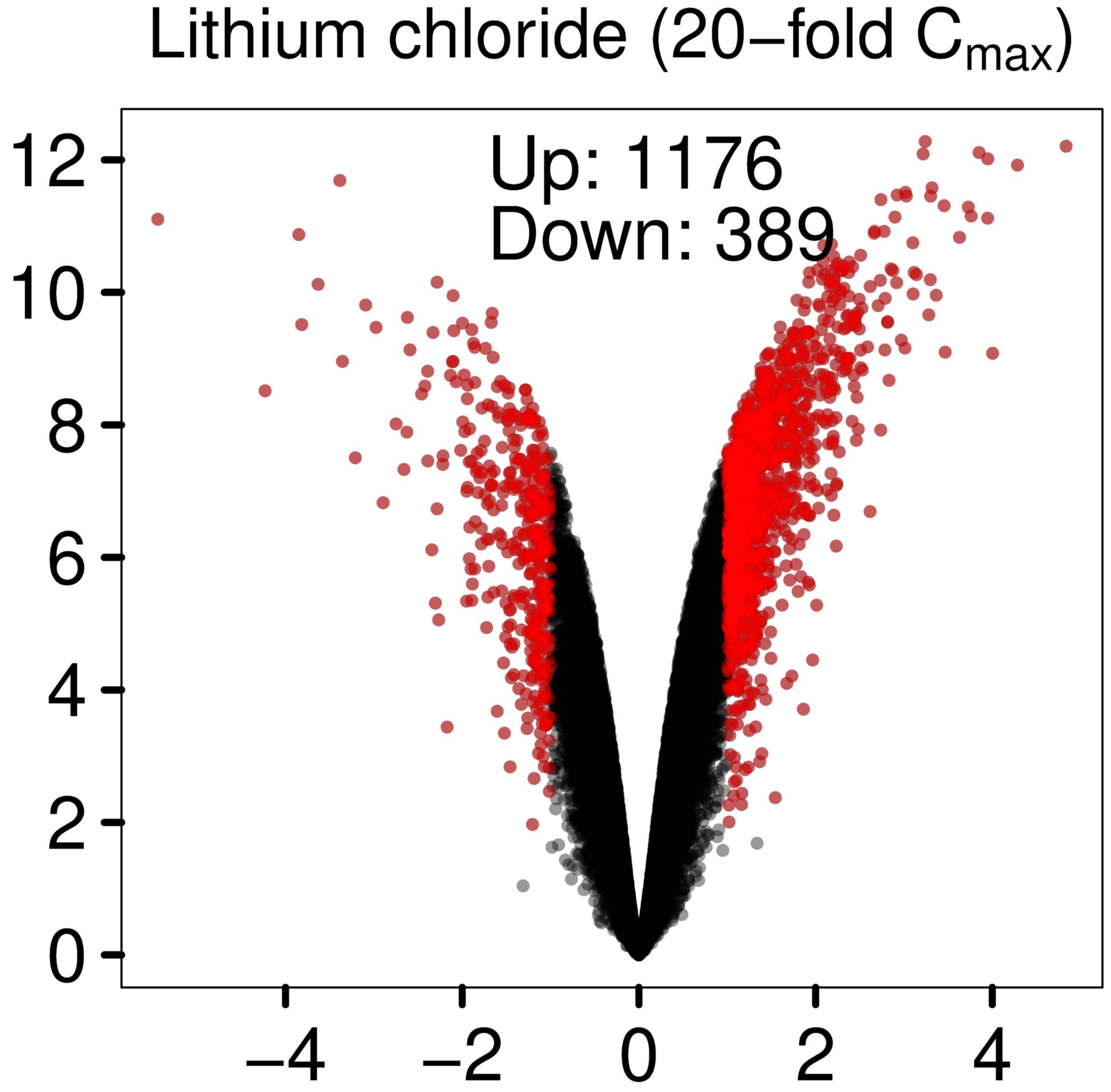
Lithium chloride (20−fold C_{max})
Up: 1176
Down: 389
12
10
8
6
4
2
0
−4
−2
0
2
4

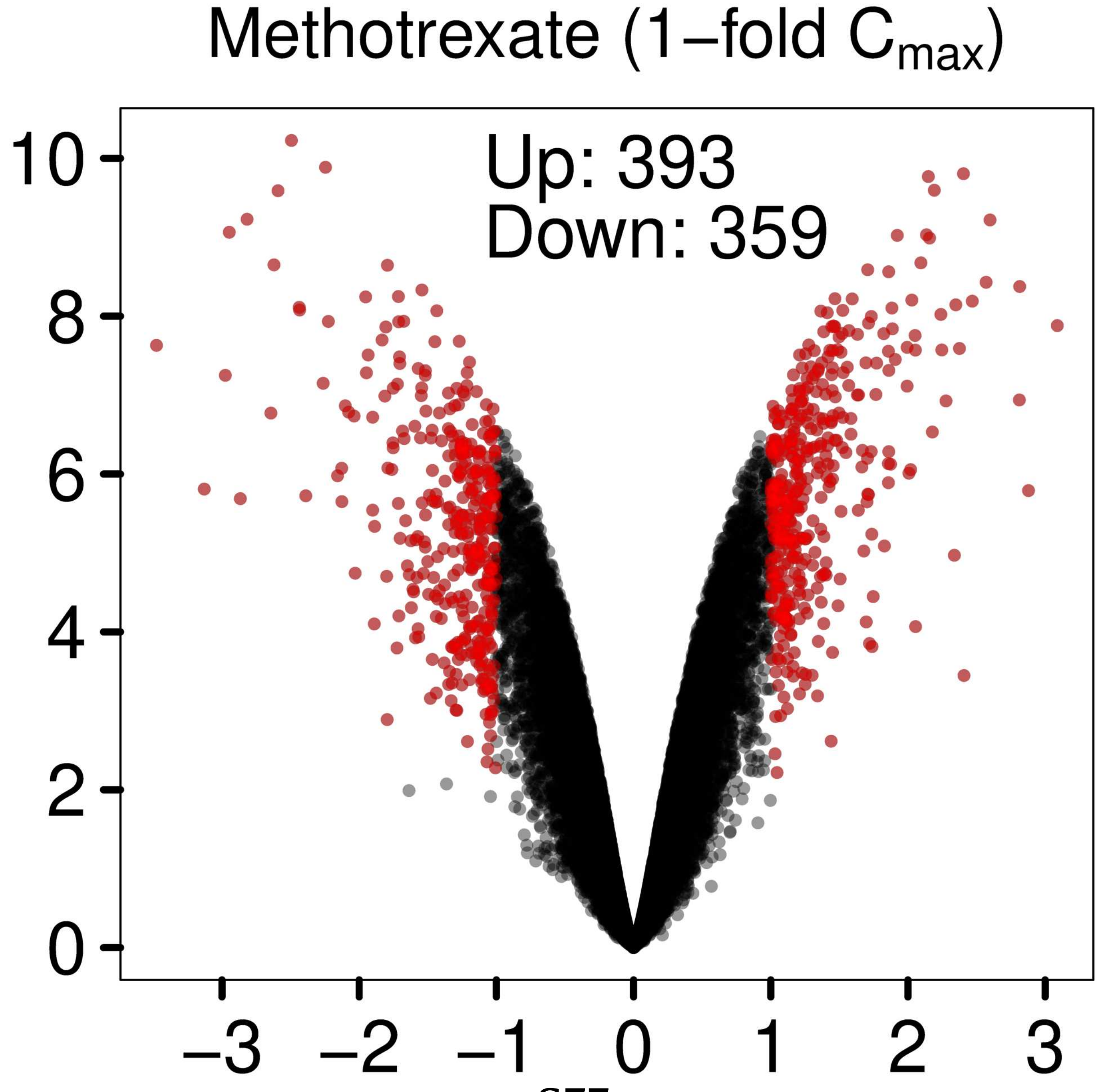
Methotrexate (1–fold C_{max})
Up: 393
Down: 359
10
8
6
4
2
0
−3
−2
−1
0
1
2
3

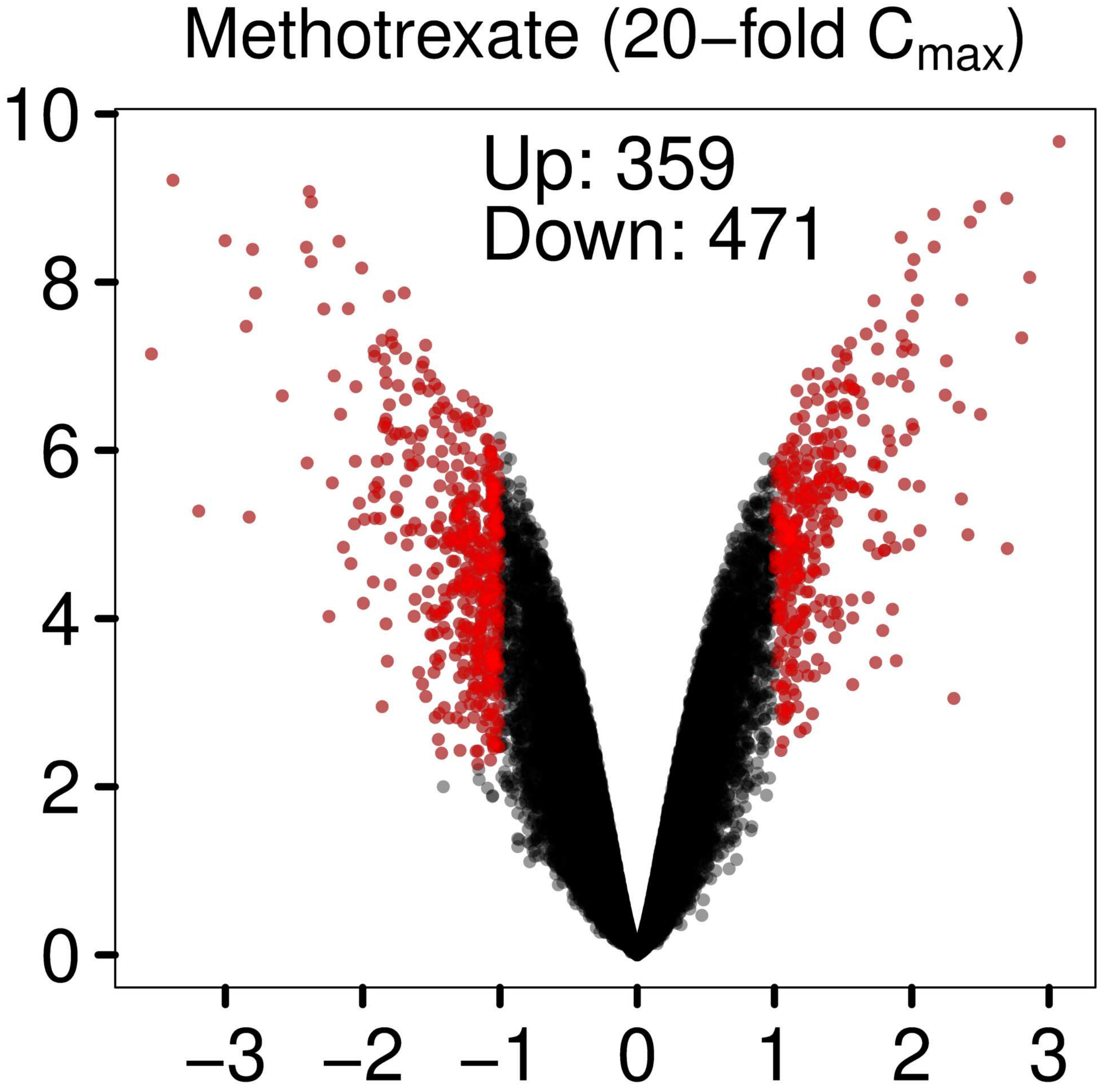
Methotrexate (20−fold C$_{max}$)
Up: 359
Down: 471
10
8
6
4
2
0
−3
−2
−1
0
1
2
3

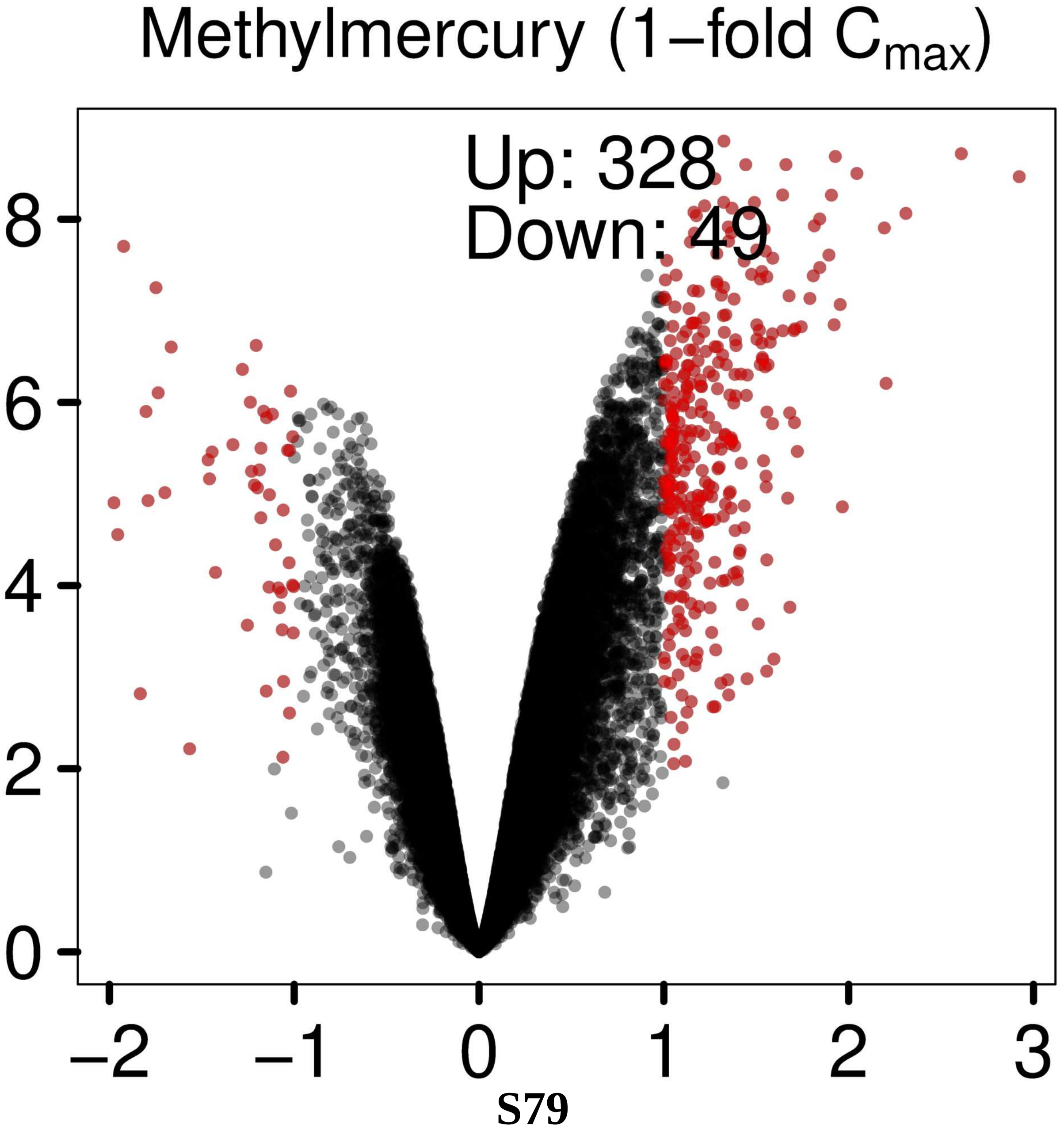
Methylmercury (1−fold C_{max})
Up: 328
Down: 49
8
6
4
2
0
−2
−1
0
1
2
3

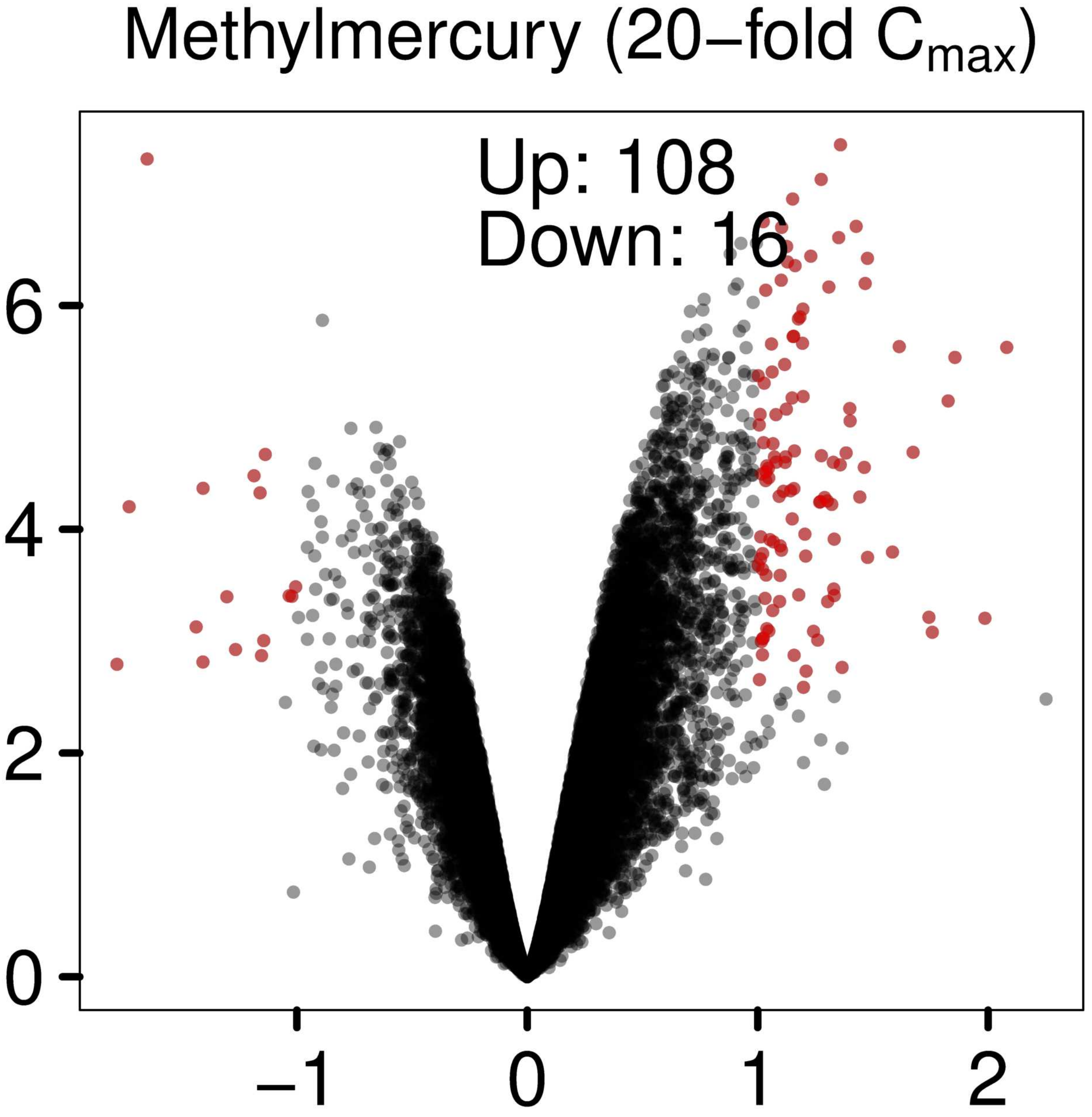
Methylmercury (20−fold C_{max})
Up: 108
Down: 16
6
4
2
0
−1
0
1
2

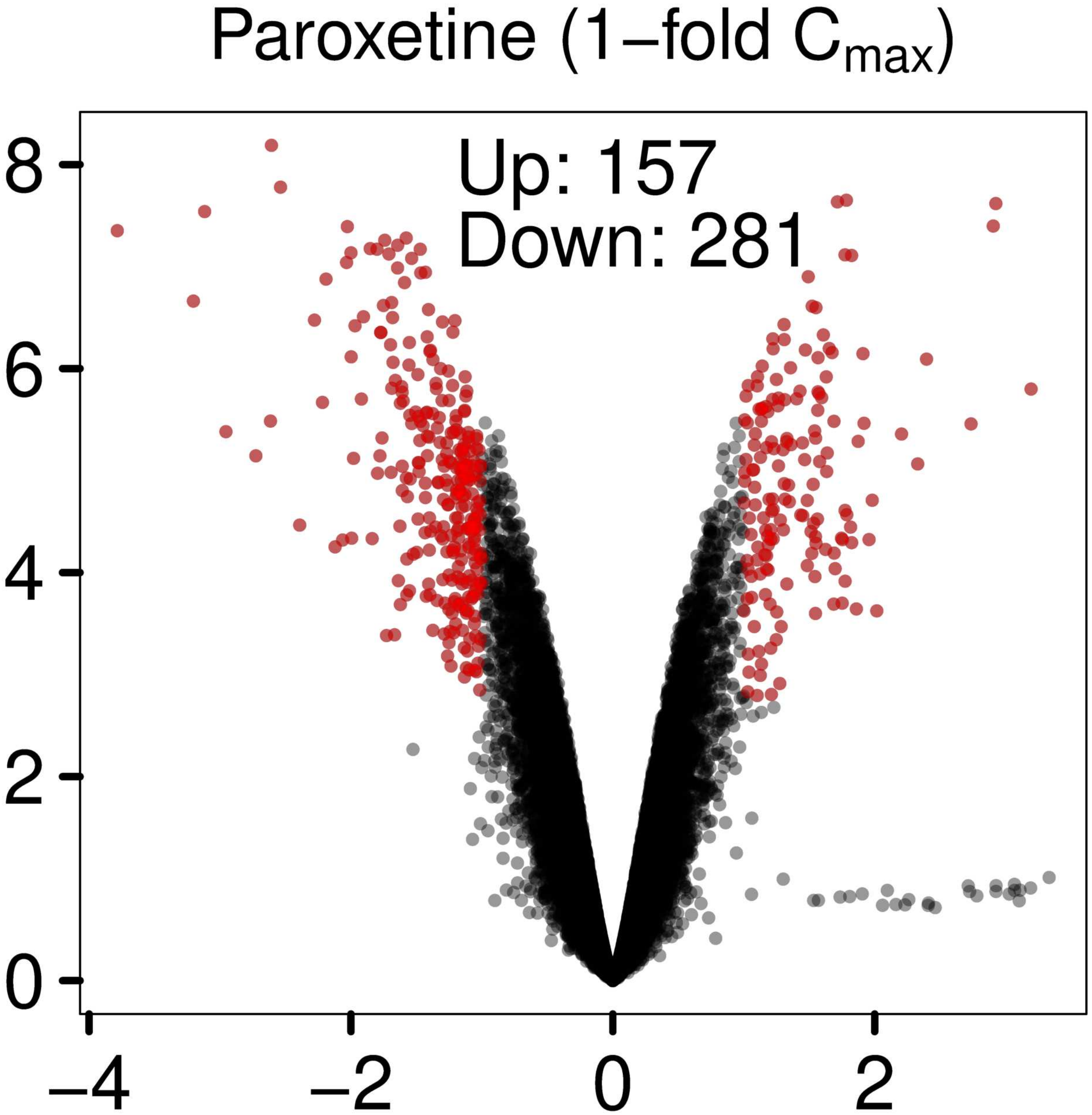
Paroxetine (1−fold C_{max})
Up: 157
Down: 281
8
6
4
2
0
−4
−2
0
2

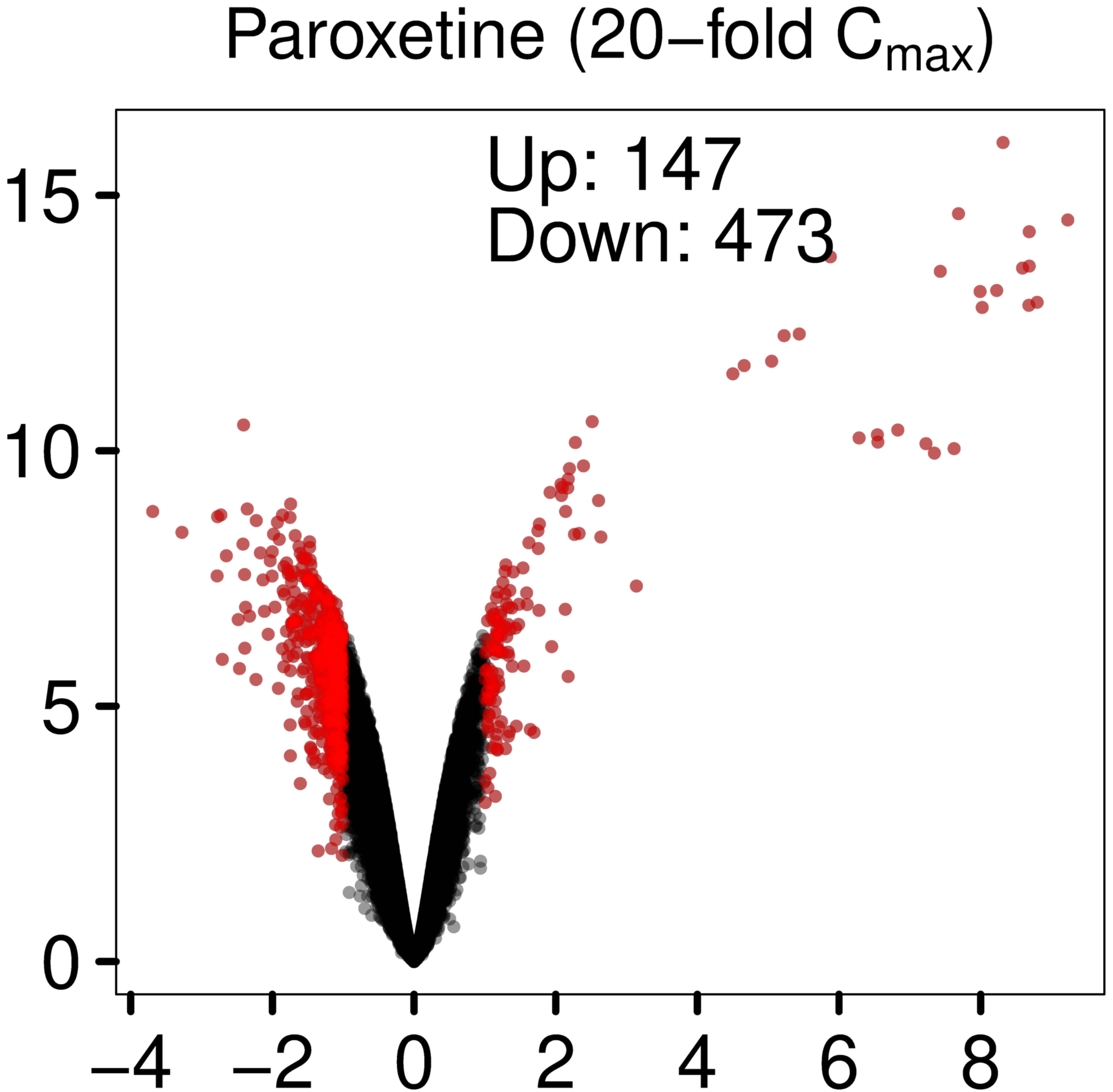
Paroxetine (20−fold C_{max})
Up: 147
Down: 473
15
10
5
0
−4
−2
0
2
4
6
8

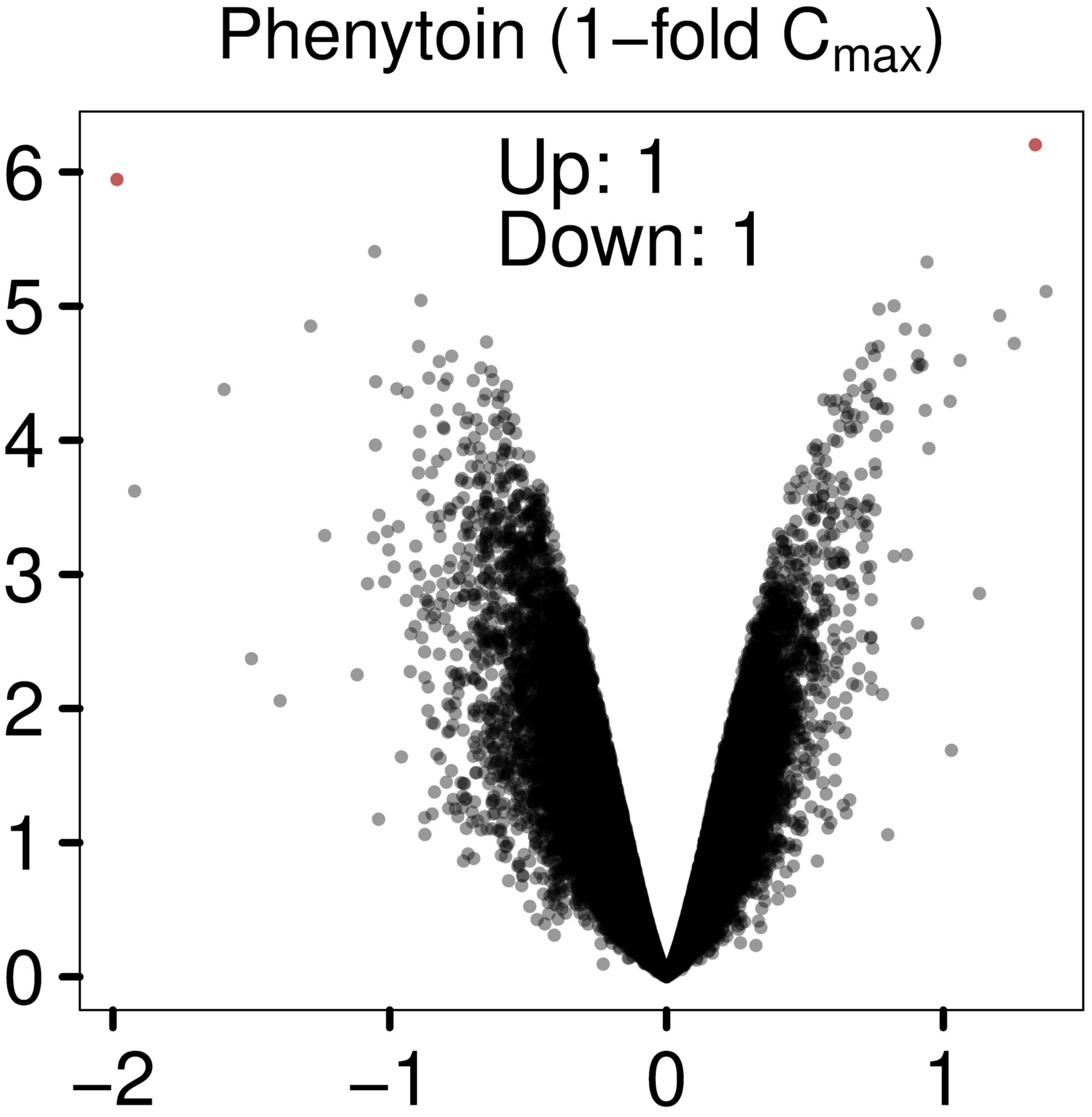

S83

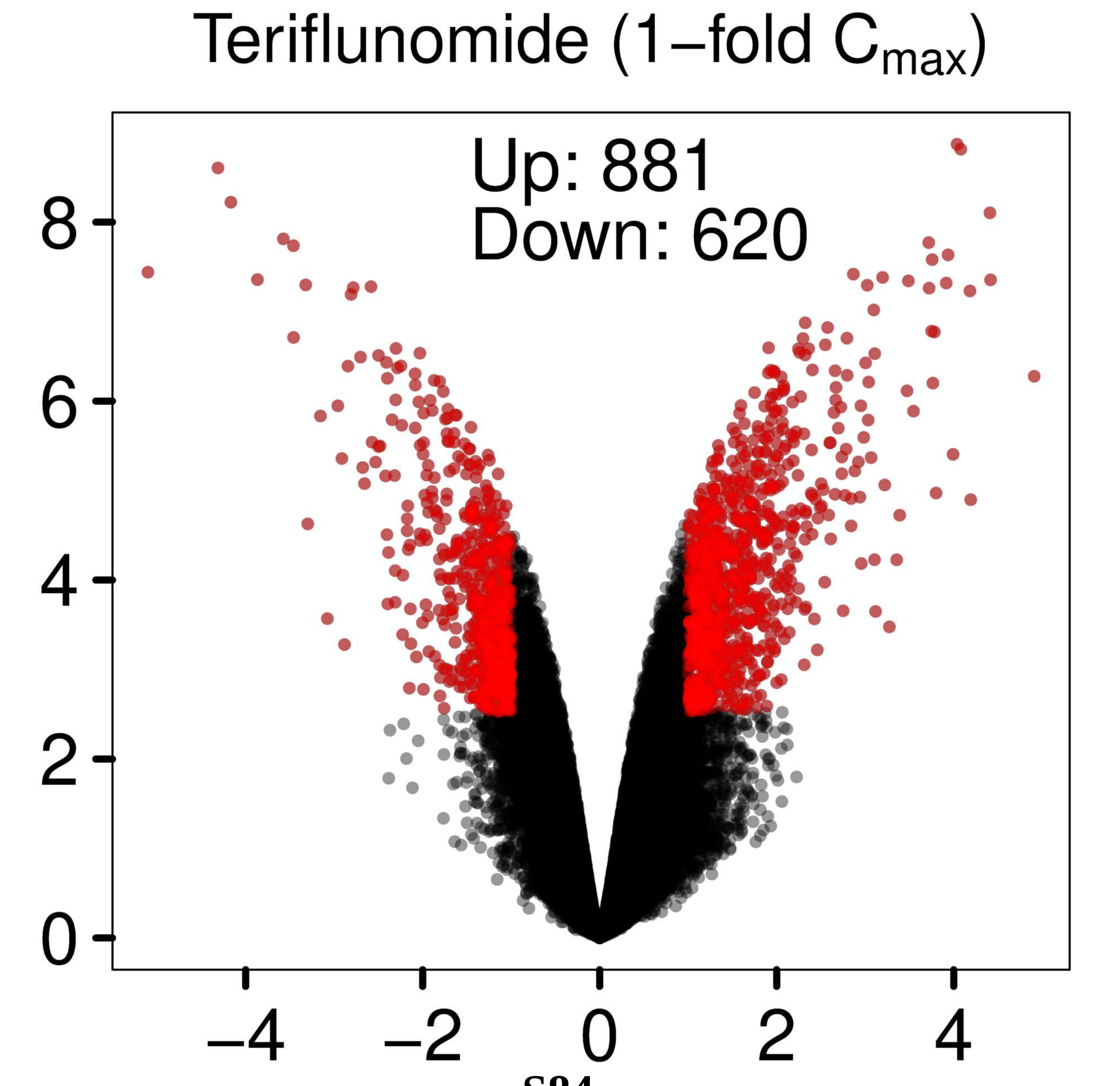
Teriflunomide (1–fold C_{max})
Up: 881
Down: 620
8
6
4
2
0
–4
–2
0
2
4

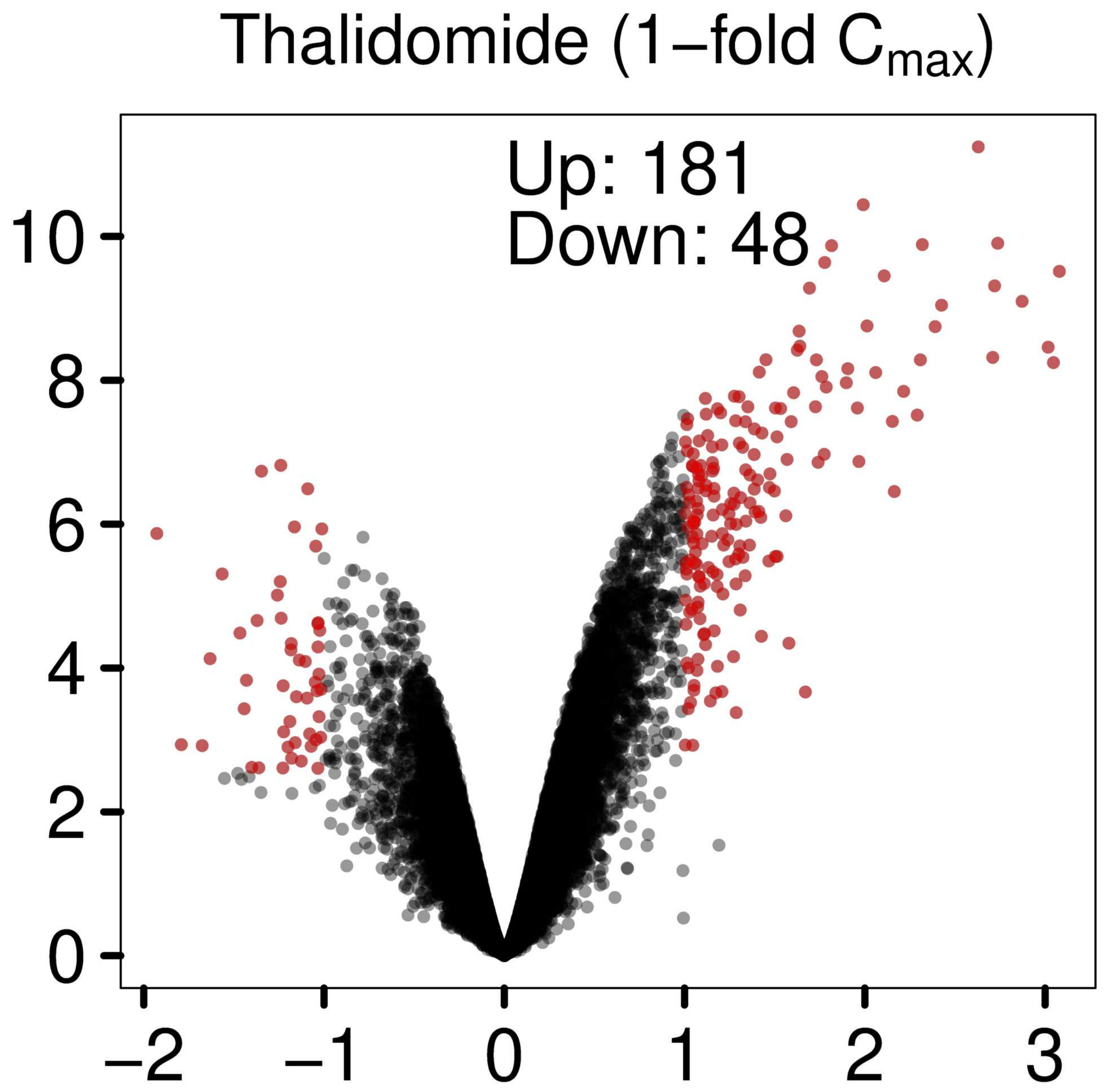
Thalidomide (1−fold C_{max})
Up: 181
Down: 48
10
8
6
4
2
0
−2
−1
0
1
2
3

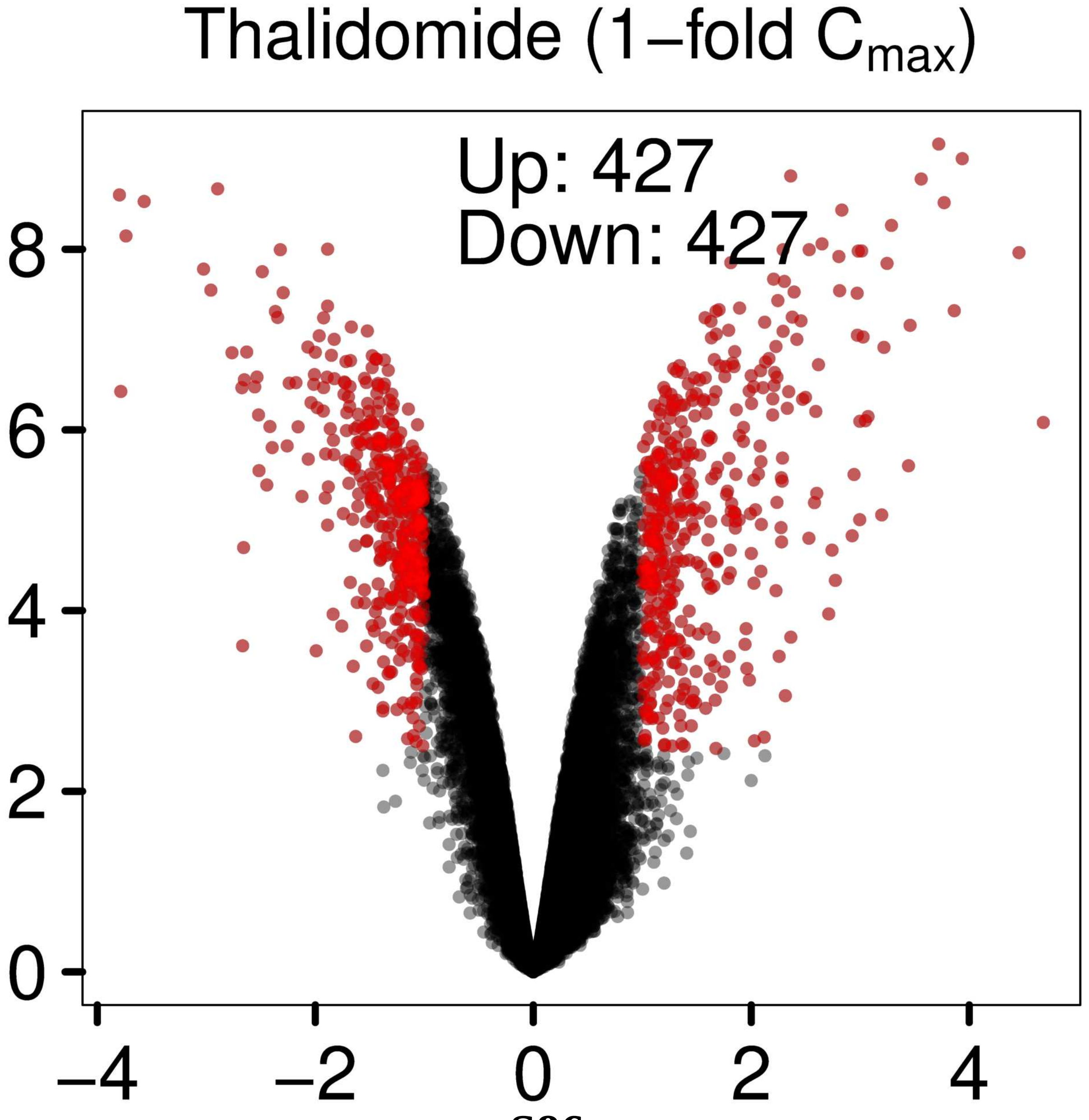

S86

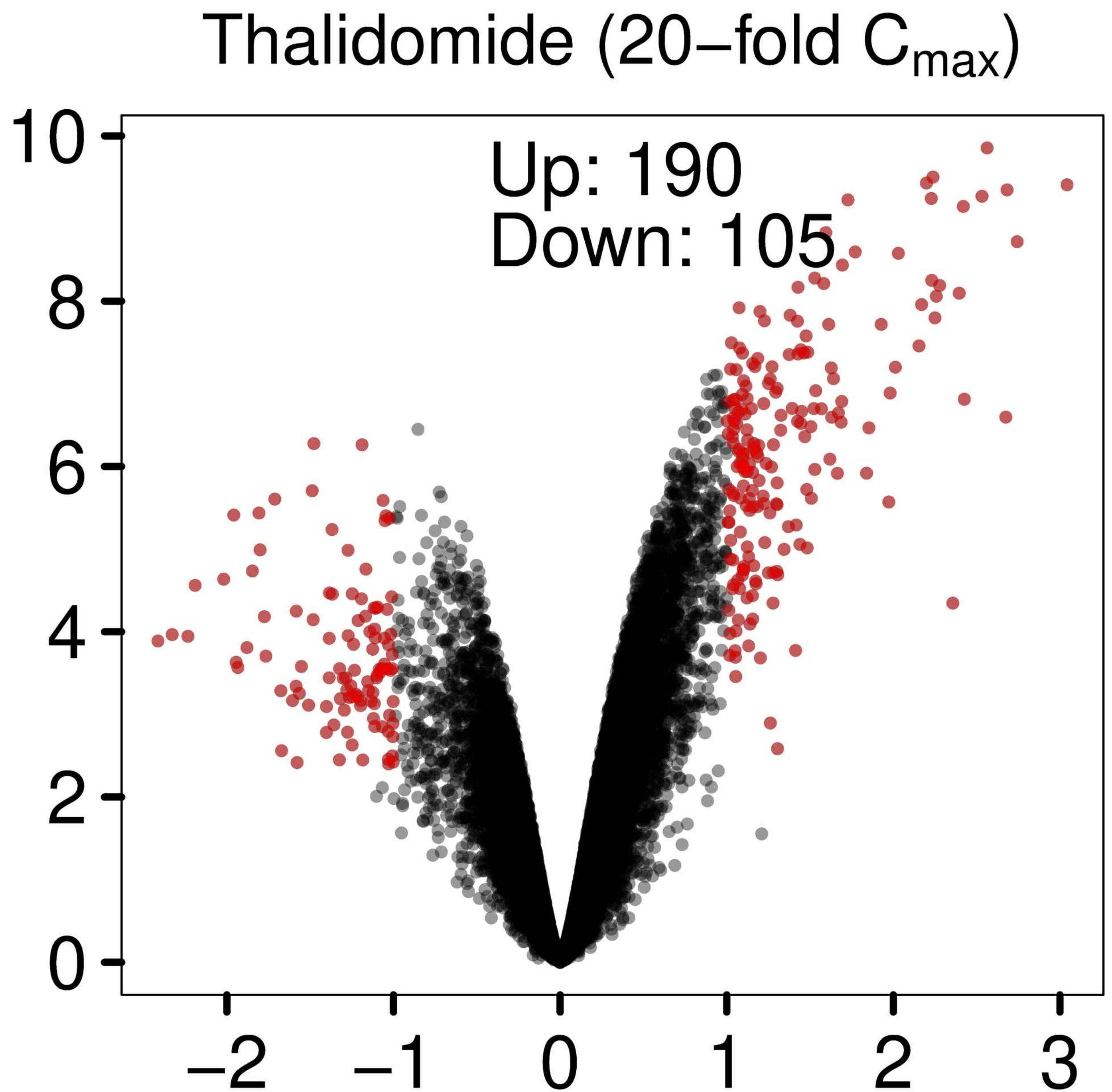
Thalidomide (20–fold C_{max})
Up: 190
Down: 105
10
8
6
4
2
0
−2
−1
0
1
2
3

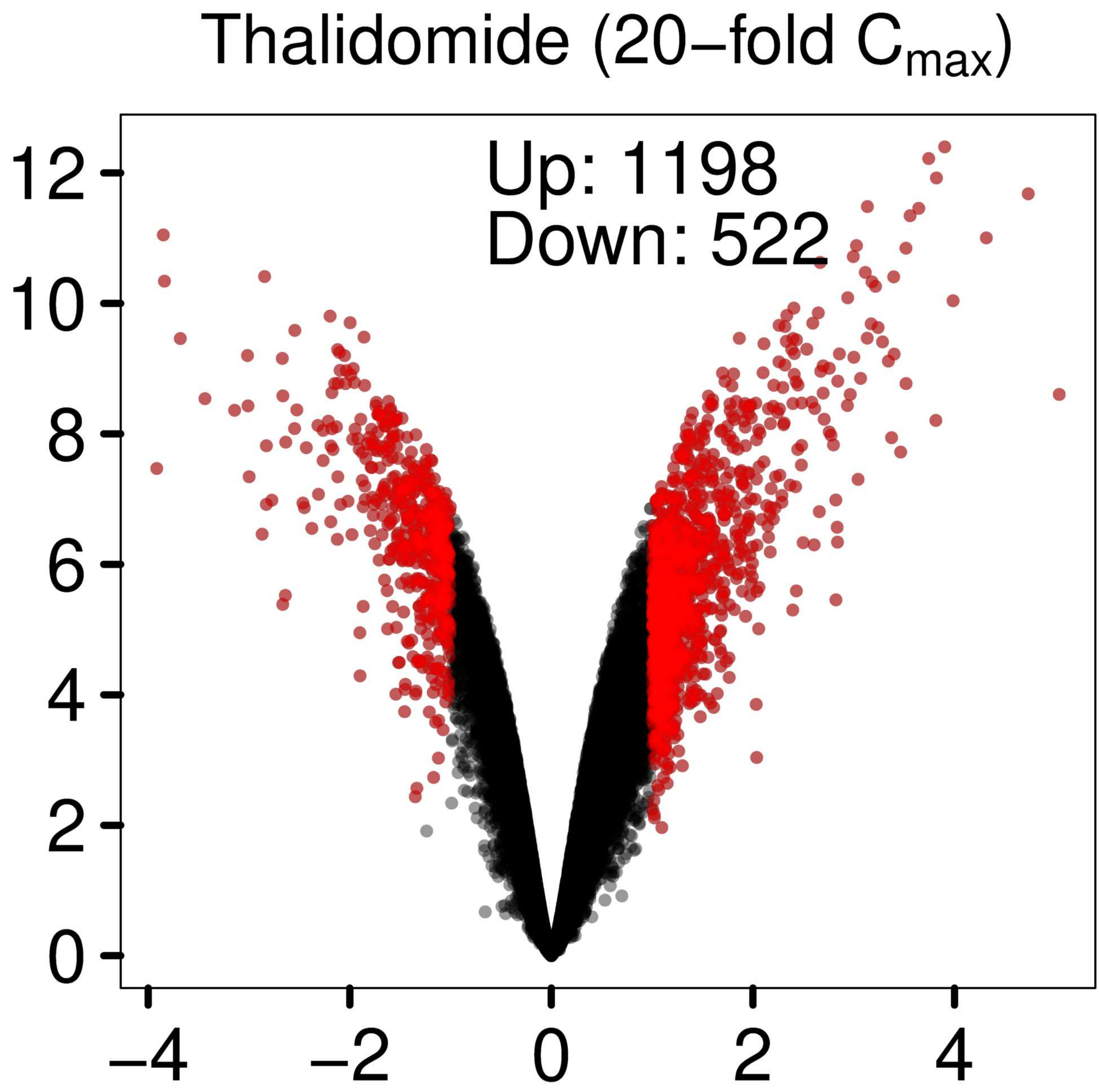

Thalidomide (20–fold C_{max})
Up: 1198
Down: 522
12
10
8
6
4
2
0
–4
–2
0
2
4

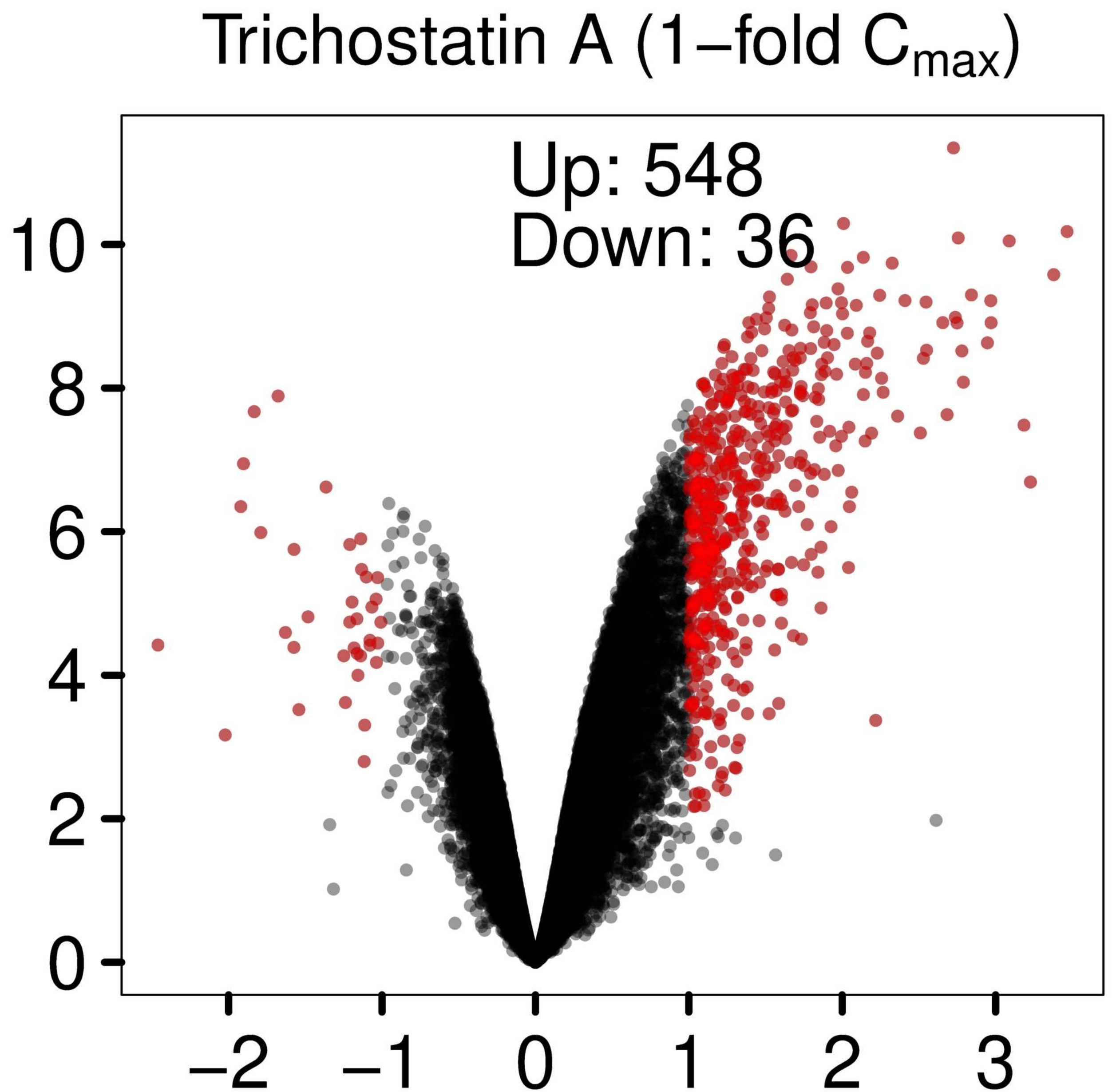
Trichostatin A (1–fold C$_{max}$)
Up: 548
Down: 36
0
2
4
6
8
10
–2
–1
0
1
2
3

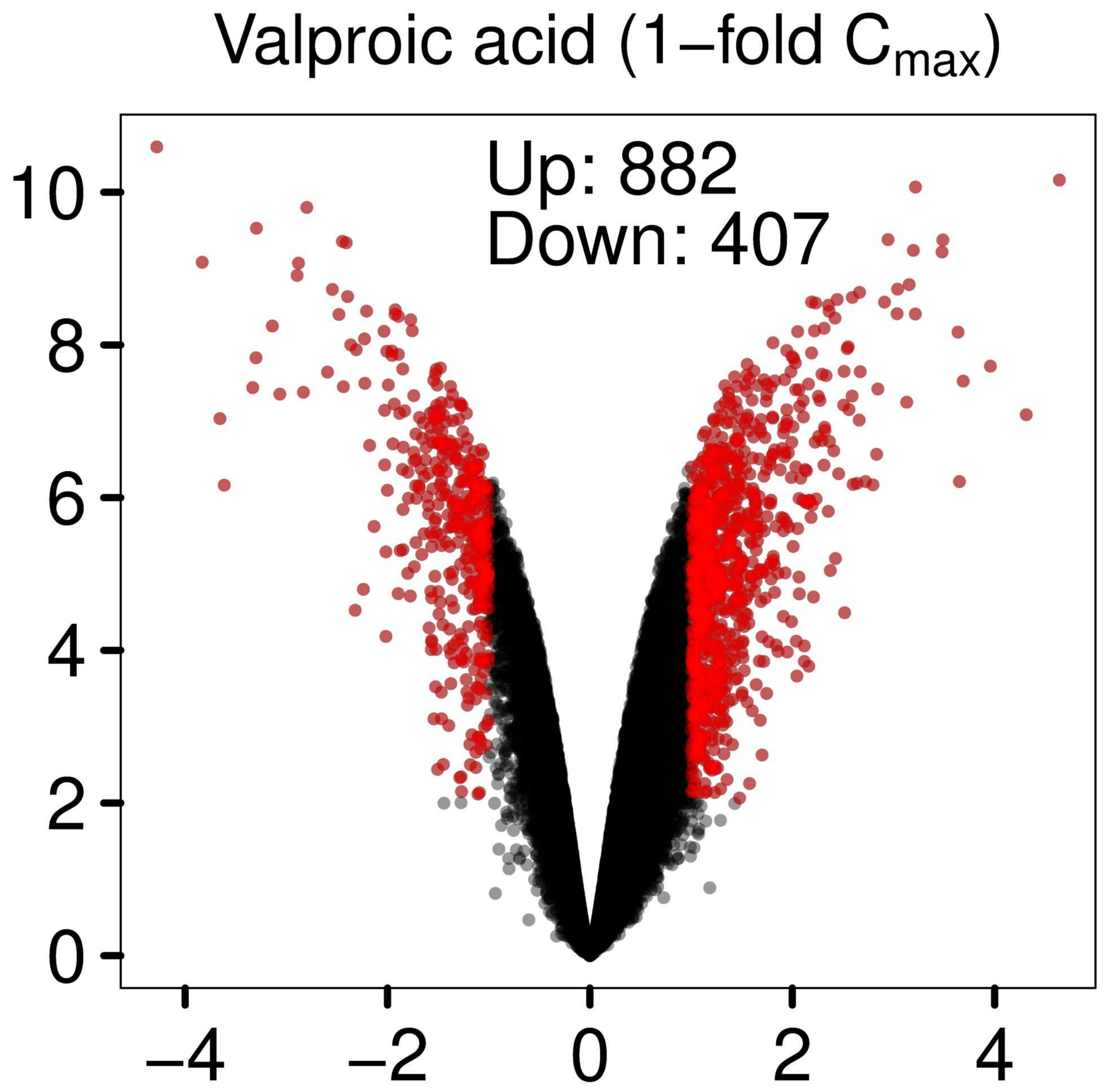

Valproic acid (1−fold C_{max})
Up: 882
Down: 407
10
8
6
4
2
0
−4
−2
0
2
4

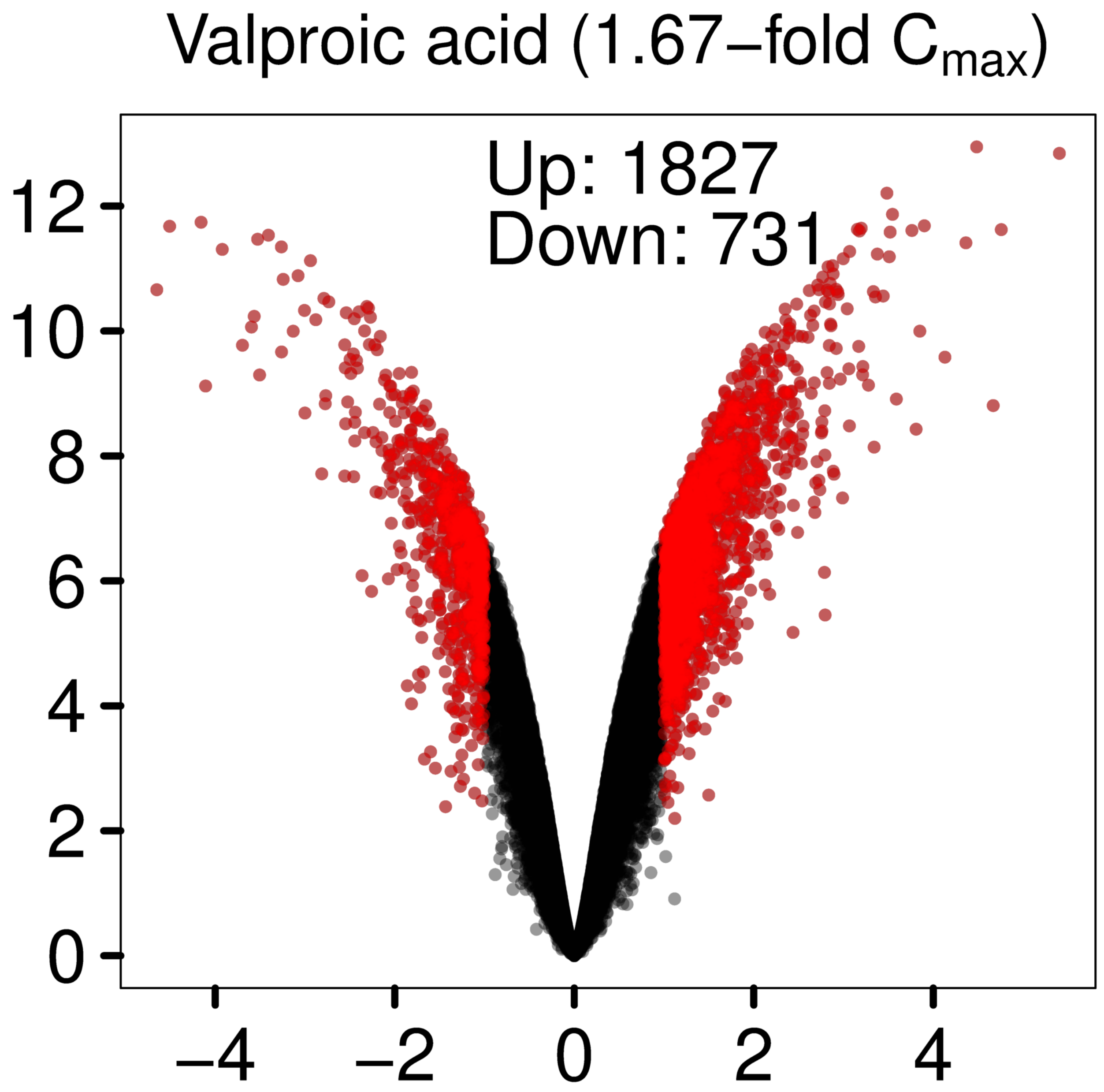
Valproic acid (1.67–fold C_{max})
Up: 1827
Down: 731
12
10
8
6
4
2
0
−4
−2
0
2
4

S91

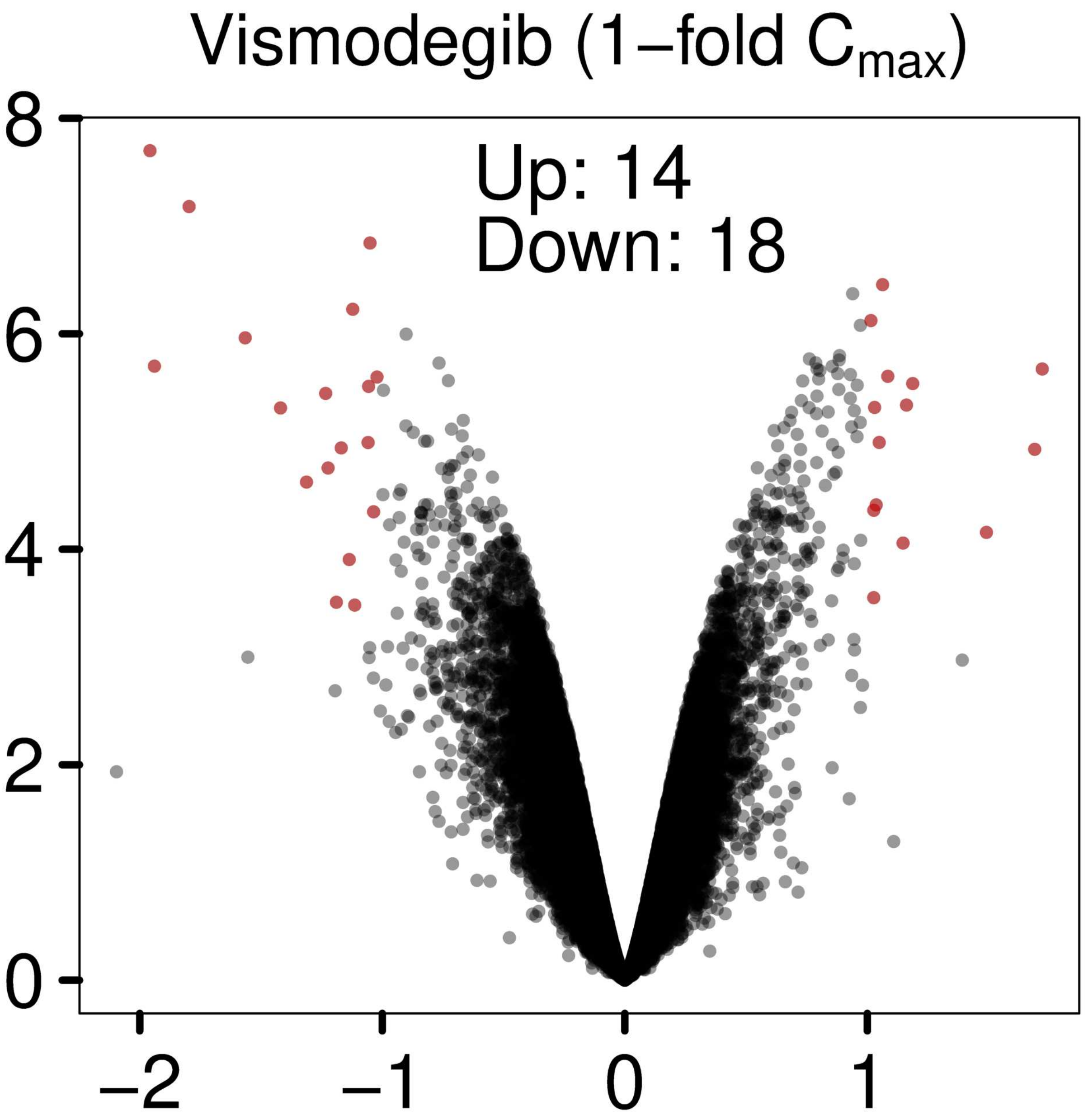
Vismodegib (1−fold C_{max})
Up: 14
Down: 18
8
6
4
2
0
−2
−1
0
1
